# Effects of exercise on cognition and Alzheimer's biomarkers in a randomized controlled trial of adults with mild cognitive impairment: The EXERT study

**DOI:** 10.1002/alz.14586

**Published:** 2025-04-24

**Authors:** Laura D. Baker, Judy A. Pa, Jeffrey A. Katula, Vahan Aslanyan, David P. Salmon, Diane M. Jacobs, Elizabeth A. Chmelo, Heather Hodge, Rosemary Morrison, Genevieve Matthews, James Brewer, Youngkyoo Jung, Robert A. Rissman, Curtis Taylor, Gabriel C. Léger, Karen Messer, A. Carol Evans, Ozioma C. Okonkwo, Aladdin H. Shadyab, Jingjing Zou, Sheila Jin, Ronald G. Thomas, Jin Zhang, Andrea Z. La Croix, Carl W. Cotman, Howard H. Feldman, for the EXERT Study Group

**Affiliations:** ^1^ Department of Internal Medicine‐Geriatrics Wake Forest University School of Medicine Winston Salem North Carolina USA; ^2^ Departments of Social Science & Health Policy, and Epidemiology, Division of Public Health Sciences Wake Forest University Health Sciences Winston Salem North Carolina USA; ^3^ Alzheimer's Disease Cooperative Study University of California La Jolla California USA; ^4^ Department of Neurosciences University of California La Jolla California USA; ^5^ Department of Health and Exercise Science Wake Forest University Winston Salem North Carolina USA; ^6^ Department of Population and Public Health Sciences, Keck School of Medicine University of Southern California Los Angeles California USA; ^7^ YMCA of the USA Chicago Illinois USA; ^8^ Department of Radiology, Medical Physics University of California Davis Health Davis California USA; ^9^ Department of Physiology and Neurosciences, Keck School of Medicine University of Southern California Los Angeles California USA; ^10^ Herbert Wertheim School of Public Health and Human Longevity Science University of California San Diego La Jolla California USA; ^11^ Moors Cancer Center University of California San Diego La Jolla California USA; ^12^ Department of Medicine, Division of Geriatrics and Gerontology University of Wisconsin‐Madison Madison Wisconsin USA; ^13^ Department of Medicine, Division of Geriatrics, Gerontology, and Palliative Care University of California San Diego La Jolla California USA; ^14^ Shiley‐Marcos Alzheimer's Disease Research Center University of California San Diego La Jolla California USA; ^15^ Department of Neurobiology and Behavior; Institute for Memory Impairments and Neurological Disorders University of California Irvine California USA

**Keywords:** Alzheimer's, brain MRI, clinical trial, cognition, exercise, mild cognitive impairment, multisite, nonpharmacological intervention

## Abstract

**INTRODUCTION:**

The EXERT study (Exercise in Adults with Mild Memory Problems) was a Phase 3, multicenter, randomized controlled trial that examined effects of exercise on cognition and other measures of brain health in sedentary older adults with amnestic mild cognitive impairment (MCI).

**METHODS:**

Participants were randomized to moderate‐high intensity aerobic training (AX) or low‐intensity stretching/balance/range of motion (SBR) for 18 months. Exercise was supervised for the first 12 months. Assessments were administered at baseline and every 6 months. The primary outcome was a global cognitive composite.

**RESULTS:**

A total of 296 participants were enrolled, and intervention adherence was high (supervised session attendance: AX = 81%, SBR = 87%). Intervention effects on cognition did not differ for AX and SBR (regression = –0.078, standard error [SE] = 0.074; *p *= 0.3). Notably, there was no 12 month  cognition decline for either group, and mean 12 month  hippocampal volume loss for both groups was low at 0.51%.

**DISCUSSION:**

Exercise intensity did not differentially affect cognitive trajectory. Intervention delivery was successful (high adherence) and cognition remained stable over 12 months  for both MCI groups, an association that warrants further study.

**Highlights:**

- Exercise in Adults with Mild Memory Problems (EXERT) was a large multisite randomized controlled trial of moderate‐high intensity aerobic training versus lower‐intensity flexibility and balance exercise in sedentary older adults with amnestic mild cognitive impairment (MCI).
- A sensitive and validated measure of global cognitive function, the Alzheimer's Disease Assessment Scale‐Cognition supplemented with tests of executive function (ADAS‐Cog‐Exec), was used to assess intervention efficacy with 12 months of supervised exercise.
- There was no intervention group difference on the 12‐month cognitive trajectory of the ADAS‐Cog‐Exec.
- Intervention delivery was successful (high adherence), and cognition remained stable over 12 months for both exercise groups.
- Regular supported moderate‐high or lower‐intensity exercise may stall decline in adults with amnestic MCI, but further investigation is needed.

## 1 BACKGROUND

An urgent need exists to find effective treatments for Alzheimer's disease (AD) that can arrest or reverse the disease at its earliest stage, as it affects more than 46 million people worldwide now and >131 million by 2050.^1^ Amnestic mild cognitive impairment (MCI), characterized by mild memory loss often accompanied by mild executive dysfunction, is the typical clinical phenotype of early‐stage AD, and a prodrome of clinical dementia. The potential to preserve, or even enhance, cognition in adults at high risk of cognitive decline due to neurodegenerative disease has important implications, not only for the affected individual but also for the support system that bears the social and financial burdens of long‐term caregiving. However, prior studies testing interventions to prevent or arrest cognitive decline in MCI using a variety of pharmaceutical approaches have been largely negative.^2^, ^3^ Two U.S. Food and Drug Administration (FDA)–approved anti‐amyloid monoclonal antibodies are now available to slow AD by a few months.^4^, ^5^ However, these treatments can have life‐threatening adverse side effects and come at a cost of over $25,000 per year. Moreover, a large proportion of individuals with MCI either will not have access to or will not qualify to receive these treatments. Thus there is a pressing need to identify efficacious and cost‐effective strategies to protect cognition against decline that are readily accessible to a large number of people. A seminal 2020 Lancet Commission report^6^ that quantified risk of dementia due to various early and late life exposures and lifestyle behaviors has been instrumental in pushing risk modification forward as a high priority in dementia prevention. The long prodromal period associated with MCI provides an ideal opportunity for lifestyle modifications—such as increased physical activity—to protect and enhance cognition when pathologic processes that cause decline can still be prevented or delayed.

Although not without controversy,^7^ the majority of cross‐sectional and longitudinal epidemiologic studies to date^8^, ^9^, ^10^, ^11^, ^12^, ^13^, ^14^ or published reviews^15^ indicate reduced risk of cognitive decline with increased physical activity, even among the oldest old.^16^ A 2018 meta‐analysis of 1145 older adults with MCI across 119 studies, 89% of which were randomized controlled trials (RCTs), reported a 0.47 standard deviation (SD) benefit of exercise on cognition, which was more robust for aerobic exercise (0.65 SD).^17^

Meta‐analyses of RCTs testing the effects of physical exercise on cognition in cognitively healthy adults or individuals with MCI have reported mixed results.^18^, ^19^, ^20^, ^21^, ^22^ Although some reports support some cognitive benefit in MCI,^18^, ^21^, ^22^, ^23^ others fail to show a consistent positive signal.^19^, ^20^ Key issues identified in these reviews include small sample sizes; variability in participant characteristics (e.g., how MCI/dementia is defined), study protocols (e.g., home‐based unsupervised vs facility‐based supervised exercise; differences in total amount of weekly exercise), adherence monitoring (e.g., self‐report or objective), and outcomes (e.g., scores on individual tests vs composite scores); and the need for more biomarker data to advance mechanistic investigations. The 2020 Lancet Commission report identified increased physical exercise as a target behavior to reduce an estimated 2% of population attributable risk for dementia.^6^ In 2023, a collaborative international guideline for the prevention and management of MCI and dementia^24^ stated that although physical activity may be considered for primary prevention of dementia, there is uncertainty about the impact on disease progression for individuals with MCI, and there is a pressing need for adequately powered RCTs to address this gap.

EXERT (Therapeutic Effects of Exercise in Adults with Amnestic Mild Cognitive Impairment) was a Phase 3 multisite RCT designed to provide high statistical power, rigor in intervention delivery and adherence, inclusion of individuals most likely to benefit from the intervention, and reliable and validated outcomes that are harmonized with other large studies to permit data sharing and expanded analyses. The purpose of the study was to assess the effects of a 12‐month supervised program of moderate‐high intensity aerobic exercise versus low‐intensity stretching/balance/range of motion exercise on global cognitive function, executive function, and episodic memory, and on other measures of brain health in sedentary adults with amnestic MCI. Our primary hypothesis, based on previous studies^25^, ^26^, ^27^, ^28^, ^29^, ^30^, ^31^, ^32^, ^33^ and comprehensive reviews,^34^, ^35^ was that higher intensity exercise would be more efficacious than lower intensity exercise on all outcomes.

## 2 METHODS

The recruitment strategies, enrollment timeline, and cohort characteristics are published^36^; the protocol is provided as a Supplement. The study was conducted in accordance with The Code of Ethics of the World Medical Association and was approved by the institutional review boards of the University of California, San Diego, Wake Forest University School of Medicine, and the 13 participating clinical sites.

**RESEARCH IN CONTEXT**

1. **Systematic review**: The literature was reviewed using PubMed and Medline. There are mixed reports from randomized controlled trials (RCTs) regarding the efficacy of regular exercise on cognitive function in adults with mild cognitive impairment (MCI).
2. **Interpretation**: Exercise in Adults with Mild Memory Problems (EXERT) was a large multicenter RCT of moderate‐high intensity aerobic training versus lower‐intensity flexibility and balance exercise in 296 adults with amnestic MCI. Exercise intensity did not differentially affect 12‐month cognitive trajectory. However, neither intervention group showed a decline in cognition as might be expected for adults with amnestic MCI.
3. **Future directions**: EXERT findings suggest that regular, supported, moderate‐high or low‐intensity exercise may stall decline in adults with amnestic MCI, but further investigation is warranted.

### 2.1 Study design overview

**FIGURE 1 alz14586-fig-0001:**
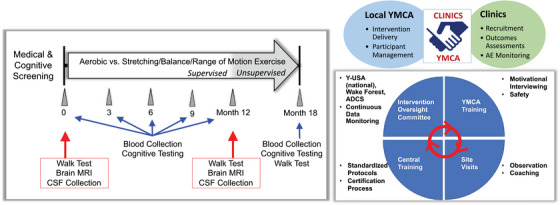
Study design and intervention oversight.

EXERT was a large multisite RCT examining the effects of moderate‐high intensity (aerobic) exercise (AX) versus low‐intensity stretching, balance, range of motion exercise (SBR) on cognitive trajectory in a planned sample of 300 sedentary older adults with amnestic MCI (clinicaltrials.gov: NCT02814526). Participants were randomized to the AX group or SBR group and completed their assigned interventions at a local YMCA with a study‐certified trainer for 12 months, and without supervision (independently) for an additional 6 months. Centralized training and intervention oversight, with regular site contact, supported intervention fidelity. Outcomes assessments were completed at baseline (pre‐randomization) and at months 6, 12, and 18, with the primary endpoint pre‐specified as change during the supervised phase of the intervention (see Figure 1). The primary outcome was the ADAS‐ Cog‐ Exec (Alzheimer's Disease Assessment Scale‐Cognition‐Executive).^37^ The Alzheimer's Disease Cooperative Study (ADCS) at the University of California, San Diego, coordinated the trial, in partnership with Wake Forest University School of Medicine and the YMCA of the USA (Y‐USA) for intervention delivery oversight.

### 2.2 Participants

Targeted enrollment was 300 older adults (65–89 years of age) with amnestic MCI at 13 clinical sites across the United States. Sedentary status was assessed during an initial screening visit (typically by telephone) using a modified Telephone Assessment of Physical Activity (TAPA) questionnaire^38^ to identify adults who were not already regular exercisers and thus largely sedentary. Light physical activity (e.g., leisure walking and light house or yard work) and more strenuous but brief and infrequent activity (e.g., <20 min/day for no more than 2 days/week) were not exclusionary (see Supplement for EXERT‐modified TAPA). Amnestic MCI was identified by global CDR = 0.05 with memory box score of ≥0.5^10^; Mini‐Mental State Examination (MMSE) ≥24 for participants with 13+ years education or ≥22 for participants with ≤12 years education; modified Hachinski score ≤4^11^; and confirmation of diagnostic impression by the study clinician based on test scores and clinical ratings. Other key eligibility criteria included willing and able to safely participate, to be randomized to either intervention, and to travel to the YMCA four times per week; no significant neurologic disease (other than MCI); no history of significant psychiatric disease within the previous 12 months; no history of significant cardiovascular events (e.g., myocardial infarction, coronary artery angioplasty, bypass grafting, stent) within the previous 6 months; no alcohol/substance abuse within the previous 2 years; hemoglobin A1c ≤7; no insulin use for type 2 diabetes; no use of psychoactive medications within 60 days of screening (e.g., neuroleptics, chronic anxiolytics, sedative hypnotics; amitriptyline and benzodiazepines allowed if prescribed for sleep and use could be restricted to twice/week during trial and not before clinic visits); no past use of anti‐amyloid medications; and availability of a study partner. Eligibility was assessed based on self‐report during telephone screening and the clinician interview at the first clinic visit. Of note, eligibility criteria were modified from the initial protocol ≈1 year into recruitment to lower screen fail rate by including community‐dwelling adults without a previously established diagnosis of MCI. The initial inclusion criteria based on Logical Memory and the Auditory Verbal Learning Test were dropped.^36^ All screened individuals provided written informed consent.[Fig alz14586-fig-0001]

### 2.3 Randomization and masking

Eligible participants were randomized using a 1:1 schedule to either the AX or SBR group. Randomization was controlled by the ADCS Data Core using a computer‐generated sequential list of random allocations for the two treatment combinations. The random allocation sequence was created using SAS statistical software (version 9.4) and was stratified by site, apolipoprotein E (*APOE*) ε4 carrier status, and sex. The site principal investigator (PI) and examiners who administered the cognitive tests and the CDR were masked to group assignment. The site study clinician and YMCA liaison who communicated with the YMCA trainers about participant progress were unmasked to group assignment but masked to outcomes. In the Coordinating Center, Project Directors and ADCS Recruitment and Clinical Operations Cores were masked to intervention, whereas the ADCS Data Core, the Intervention Oversight Team (Wake Forest, Y‐USA), and the ADCS Safety Officer were not. Unmasked site personnel shared randomization assignment with participants, completed periodic calls to check on progress and assess satisfaction with the program, and reminded participants at the start of each clinic visit to “keep the secret” and refrain from sharing information about their assigned group with other personnel. Sites were trained on procedures to protect masking about intervention assignment, which were regularly reviewed during site audits and discussed in site meetings (Note: Unmasking of an examiner/data recorder occurred only once during CDR administration at a participant's final clinic assessment.) Masked personnel had restricted access to segments of the study database and were excluded from discussions that disclosed intervention‐related information.

### 2.4 Intervention

#### 2.4.1 AX and SBR intervention groups

Both groups completed their assigned interventions at local YMCAs located near clinical sites (adaptations during the coronavirus disease 2019 [COVID‐19] pandemic are described in Section 2.9) four times per week for 45 min per session. Frequency, duration, and intensity of the EXERT exercise programs were gradually increased over the first 6 weeks to build self‐efficacy and stamina, and to reduce the risk of injury. A study‐certified trainer provided supervision for the first eight sessions, and then for two of the four weekly sessions through month 12. In months 13–18, participants continued their assigned exercise programs, but without supervision. Prior to participant contact, trainers were centrally trained and certified on the study protocol, data collection, and adverse event (AE) recording and reporting, and received training about MCI/AD and daily challenges for individuals living with memory loss. AX and SBR participants were provided with a digital wrist‐worn heart rate (HR) monitor with a chest strap for HR tracking while exercising. Participants taking HR‐lowering medications were instructed to rely more heavily on other measures to monitor effort, such as breathing rate and difficulty talking while exercising. All participants were trained to use the Rating of Perceived Exertion (RPE) modified Borg scale (ratings range from 1 = very mild, to 10 = very, very hard) in the first 2 weeks of the program as an additional means of assessing exercise effort. Proper use of the RPE scale was assessed and retrained by the trainer at regular intervals and as needed throughout the study. Adherence was evaluated and tracked using HR monitoring, RPE, and trainer and participant records of completed exercise sessions. Trainer records of supervised sessions included date/time, time in prescribed HR zone, HR logged at 5–10 min intervals, peak HR, RPE, peak RPE, exercise duration, trainer assessment of effort, and activity details (e.g., treadmill grade/speed; body parts stretched), which were entered into the ADCS web‐based electronic data capture (EDC) system for regular central review. Participants also kept a log of exercise session details (duration, mean HR, maximum RPE, activity type), which was reviewed by the trainer during supervised sessions. The infrastructure to support fidelity of intervention delivery included centralized training and oversight and regular contact with clinic and YMCA teams (Figure 1). YMCA study staff also met monthly with the clinic YMCA liaison and with the Intervention Oversight Team to review progress and challenges. The study adherence goal was for participants to complete ≥80% of their prescribed sessions (mean of 3.2 sessions/week) within their prescribed HR zone. Multiple strategies were used to facilitate adherence (e.g., motivational interviewing, make‐up sessions, group sessions, alternate exercise plans during travel, regular participant contact). When continuation of the intervention was deemed medically unsafe for a participant (e.g., after joint surgery or a fall), the intervention was temporarily paused. When the participant was cleared to restart the intervention, exercise was gradually re‐introduced over 2–4 weeks. For these individuals, clinic visits were pushed back when necessary to provide at least 6 weeks of full intensity exercise prior to an outcomes assessment.

#### 2.4.2 Aerobic training intervention

The AX intervention aligned with standard American College of Sports Medicine (ACSM) recommendations^39^ and protocols tested in smaller‐scale clinical trials.^26^, ^29^, ^33^, ^40^ The AX prescription targeted aerobic exercise for 45 min (includes warm‐up and cool‐down), four times per week, and was introduced to AX participants by slowly increasing frequency and duration over the first 6 weeks to build self‐efficacy and stamina, and to reduce risk of injury. Aerobic training targeted moderate‐high cardiorespiratory intensity (70%–80% of heart rate reserve [HRR]; HRR = 220 – age – resting HR; Target HR range = [HRR x 0.70] + resting HR –[HRR x 0.80] + resting HR) and was completed on a treadmill (most common), elliptical trainer, or stationary cycle.

#### 2.4.3 Stretching, balance, range of motion intervention

The SBR intervention included a rotating and varied routine of stretching exercises for large and small muscle groups, and activities to improve balance and range of motion. The intensity goal of SBR activities was to maintain HR at or below 35% HRR for the 45 min sessions, four times per week (Target HR < [HRR x 0.35] + resting HR). As with AX, exercise frequency and duration were increased gradually over 6 weeks to promote self‐efficacy and reduce injury risk.

### 2.5 Primary and secondary outcomes

#### 2.5.1 Primary outcome

The primary outcome was cognitive change between intervention arms as measured by the validated ADAS‐Cog‐Exec composite score,^37^ which was derived using an optimally weighted combination of subtest scores from the ADAS‐Cog13 (immediate and delayed Word Recall, Orientation) supplemented with tests of executive function (Trail‐Making Test Parts A and B, Digit Symbol Substitution Test, Word Fluency, ADAS‐Cog13 Number Cancellation), and select box scores of the CDR scale (Memory, Orientation, Judgment & Problem Solving). This pre‐specified outcome was developed to increase sensitivity and statistical power, particularly in light of prior evidence showing exercise benefits on executive function (reviewed^28^). Additional detail on the construction of composites is provided in the Statistical Analysis Plan (Supplement).

#### 2.5.2 Secondary outcomes

Secondary outcomes included episodic memory composite (ADAS‐Cog‐Exec immediate and delayed Word Recall, Cogstate Face‐Name Associative Memory Exam accuracy, CogState Behavioral Pattern Separation of Objects recall, CogState One Card Learning proportion correct); executive function composite (Trails B time, Digit Symbol Substitution Test, Word Fluency by Category [mean of animals, vegetables], Word Fluency by Letter [mean of F, L], ADAS‐Cog13 Number Cancellation, National Institutes of Health [NIH] Toolbox Flanker accuracy, NIH Toolbox Dimensional Change Card Sort accuracy, CogState One Back proportion correct); brain deformation and brain perfusion of hippocampus, prefrontal composite (superior frontal, causal‐middle frontal, rostral‐middle frontal, pars opercularis, pars triangularis), AD signature composite (combines regions showing cortical atrophy with progression to AD: parahippocampus, fusiform, inferior temporal, middle temporal, inferior parietal)^41^, ^42^, ^43^; cerebrospinal fluid (CSF) levels of amyloid beta 40 (Aβ40), Aβ42, total tau (t‐tau) and phosphorylated tau‐181 (p‐tau‐181); and plasma levels of Aβ40 and Aβ42.

#### 2.5.3 Exploratory outcomes

Exploratory outcomes consisted of ADAS‐Cog13 total score, CDR–Sum of Boxes (CDR‐SB), ADCS Activities of Daily Living for MCI (ADCS‐ADL‐MCI), EuroQol (EQ‐5D), Behavior Rating Inventory of Executive Function for Adults (BRIEF‐A), Geriatric Depression Scale (GDS), Neuropsychiatric Index (NPI), 36‐item Short Form Health Survey (SF‐36), Cognitive Change Index (CCI), whole brain gray matter, ventricular volume, and CSF levels of brain‐derived neurotrophic factor (BDNF).

### 2.6 Clinic visits

#### 2.6.1 Pre‐screening visit

Prior to the clinic screening visit, verbal informed consent for prescreening was obtained from interested candidates who then completed a telephone interview to assess medical eligibility, presence of memory concerns, sedentary status (using EXERT‐modified TAPA), willingness to follow the protocol, and availability of a study partner.

#### 2.6.2 Clinic screening visit

At the start of the screening visit, participants provided written informed consent. Screening procedures included medical history and medication review, physical and neurological examination, blood collection for clinical labs and biorepository storage, DNA banking and *APOE* genotyping, electrocardiography, cognitive assessment (MMSE and expanded mental status exam [3MSE], Story Recall, Auditory Verbal Learning Test), behavioral/functional assessment (CDR, CCI, GDS), 400 m Walk Test, and brain magnetic resonance imaging (MRI). Participants meeting all eligibility criteria were scheduled for the baseline assessment and provided with a wrist‐worn actigraph (ActiGraph Link) for a 10‐day recording period to be completed prior to the baseline visit (data will be presented in a separate paper).

#### 2.6.3 Baseline

Baseline procedures included confirmation of eligibility, measurement of vital signs and weight, fasted blood collection for labs and storage, AE query (for time period since last clinic visit), assessment of the primary cognitive outcome (ADAS‐Cog‐Exec), assessment of secondary cognitive outcomes via iPad (i.e., Cogstate, NIH Toolbox), behavioral/functional assessments, and completion of self‐assessment questionnaires by the study partner (SF‐36, health care utilization, EQ‐5D as it relates to participant). Details about each cognitive test are provided in the protocol (Supplement). Lumbar puncture was completed for participants who consented to this optional procedure. Upon completion of all baseline assessments, participants were randomized to either the SBR or AX group by the ADCS Data Core using a real‐time web‐based system. A trained and certified unmasked clinic staff member disclosed randomization assignment, and participants signed a behavioral contract indicating their willingness to complete the prescribed activities.

#### 2.6.4 Follow‐up visits (months 6, 12, 18)

Outcomes assessments completed at baseline (or screening) were also completed at follow‐up visits (see protocol). Brain MRI and lumbar puncture were repeated only during the month 12 follow‐up visit, as it marked the end of the primary supervised exercise observation period. For participants who asked to exit the study prior to the 12‐month assessment, all outcomes were collected in an early termination visit.

### 2.7 Specimen collection for analysis and banking

Plasma was extracted from blood collected after a 10‐h fast during the clinic visit at baseline, and months 6, 12, and 18. For participants consenting to the lumbar puncture, CSF was collected after an overnight fast (discarding the first 1–2 mL) and aliquoted into sterile microtubes. Whole blood, plasma, and CSF were shipped to the Rissman Lab Biomarker Cores at University of California San Diego and the University of Southern California for storage and analysis. Aβ40, Aβ42, and t‐tau in plasma and CSF were assayed using previously published methods.^44^, ^45^ Validated assay platforms from Mesoscale Discovery (Rockville, Maryland, USA) were used to detect Aβ isoforms in plasma and BDNF in CSF. Fujurebio, Inc (Malvern, Pennsylvania, USA) standard enzyme‐linked immunosorbent assay (ELISA) was used to detect Aβ isoforms and t‐tau, and chemiluminescent enzyme immunoassay to detect p‐tau181 in CSF. Internal standards were used to adjust for plate‐to‐plate variation and assess freezer storage effects.^44^ *APOE* genotyping was performed using real‐time polymerase chain reaction (PCR) restriction fragment length polymorphism analysis derived from whole blood genomic DNA using QIAamp DNA blood maxi kit (Qiagen, Venlo, The Netherlands). *APOE* genotyping was run using Applied Biosystems (Foster City, California, USA) TaqMan SNP Genotyping Assay on a Bio‐Rad (Hercules, California, USA) CFX96 as described previously.^45^

### 2.8 Brain imaging

Structural MRI procedures were overseen by the ADCS Imaging Core. Arterial spin labeling (ASL) MRI was overseen by the Imaging Core and Wake Forest University School of Medicine (WFUSM). All site scanners were certified following completion of qualifying protocols. MRI included a localizer scan, followed by a high‐resolution three‐dimensional (3D) T1 structural series (magnetization‐prepared rapid gradient echo [MPRAGE] or inversion recovery spoiled‐gradient echo [IR‐SPGR]), a T2‐weighted series (fluid‐attenuated inversion recovery [FLAIR]), a diffusion weighted scan, and a gradient recalled echo scan. Sites with access to approved 3.0 Tesla (3T) scanners also included a resting‐state ASL MRI series, appended to the MRI protocol. Standard rigorous procedures were used to ensure high data integrity. Baseline and follow‐up data sets for each participant underwent spatial rigid body co‐registration followed by nonlinear registration and neuroanatomic parcellation to quantify whole‐brain and subregional volumetric change on a participant‐by‐participant basis. Corrections were made for gradient nonlinearities and intensity non‐uniformity. A clinical read of the MRI confirmed eligibility. Baseline and month 12 T1 images were used to construct the composite imaging measures. Images were processed using the longitudinal stream of Freesurfer version 7.1.1.^46^, ^47^ Robust, inverse consistent registration was used to create an unbiased within‐subject template space and image. Common information from the within‐subject template was used for processing (e.g., skull stripping, Talairach transforms, atlas registration) and initializing spherical surface maps and parcellations. Regional thicknesses for prefrontal and AD signature meta‐regions and hippocampal volumes were calculated using the Desikan‐Killiany atlas.^48^

### 2.9 Adaptations during the COVID‐19 pandemic

In response to COVID‐19 pandemic social distancing mandates in the United States, all in‐person study activities were paused for varying durations depending on regional health impact starting on March 23, 2020. That is, the study clock was paused for outcomes assessments (i.e., scheduled appointments were postponed; virtual assessments not permitted) and for intervention completion (e.g., a participant in study month 8 as of March 2020 was still in study month 8 at time of study re‐start, regardless of site's pause duration). During the pause, study staff completed weekly telephone visits with participants for AE monitoring, to encourage continued safe EXERT exercise when possible, and to obtain physical activity information for the past week (i.e., when completed, what type, how long, RPE, HR), to provide support and continuity with the study, and to assist in problem‐solving about study‐ and pandemic‐related challenges. In‐person contact at the clinics was re‐started on a rolling timeline driven by local institutional review boards starting in June 2020, with the final re‐start at the New York sites in March 2021 (Figure S1). For most sites, clinics re‐opened for in‐person contact, often weeks or even months before the YMCA could once again receive members at the facility. In these instances, adaptations were made to permit re‐start of the intervention, which included: (1) YMCA trainer‐supervised exercise outdoors; (2) virtual YMCA trainer‐supervised sessions (iPads provided as needed) that followed EXERT exercise prescriptions to the best of trainer and participant ability (i.e., trainer worked with participant to achieve prescribed frequency, duration, and intensity goals using available resources at participant's home such as stationary cycle, light weights, dance, chair exercises); and (3) virtual supervised sessions with a “central” trainer (Intervention Oversight Team member) when a local YMCA trainer was not available.

### 2.10 Safety monitoring

AEs were assessed at every clinic visit and were queried by the clinic YMCA liaison on monthly telephone calls. During supervised exercise sessions, the trainer completed an AE checklist to query change in health or physical status in the previous week with questions to identify potential serious adverse events (SAEs), which were reviewed by the study clinician. Clinic YMCA liaisons also completed monthly telephone calls to query AEs and medication updates and to provide encouragement and address challenges to continued participation as needed. Reported AEs were assessed by the study clinician and followed as appropriate. An external Data and Safety Monitoring Board (DSMB) met regularly to review safety data, vital signs, enrollment progress, and participant status.

### 2.11 Power analysis

Power calculations were based on two‐sample *t*‐tests. Sample sizes were estimated using 12‐month ADAS‐Cog11 change scores from the ADCS MCI trial,^2^ targeting power = 80%, two‐sided alpha = 5%, and SD = 4.1. Calculations ranged over effect sizes from 1.0–2.0, and dropout rates from 10%–25%, which are similar to other reports, including multicenter trials of physical activity interventions in older cohorts.^26^, ^31^, ^33^, ^40^, ^49^, ^50^ The trial was powered to conservatively accommodate 20% attrition. Based on these considerations and an estimated effect size of 1.5, a total of 291 participants were targeted for enrollment.

### 2.12 Statistical analysis

#### 2.12.1 Populations

The modified intent‐to‐treat (mITT) population was used for the primary analysis. The mITT population included all eligible individuals who (1) began the exercise intervention and (2) completed at least one post‐baseline assessment in months 6 and 12. Intervention effects through the unsupervised extended follow‐up (months 13–18) will be reported at a later time. All consented randomized participants were grouped according to treatment assigned at randomization, regardless of adherence or any protocol violations. Treatment effects in other subgroups were also explored, including: (1) “pre‐pandemic” population: completed 12‐month assessment before COVID‐19 study pause on March, 23 2020, (2) “pandemic” population: completed 12‐month assessment after pandemic study pause, (3) “per‐protocol” population: completed 12‐month assessment for the primary analysis, attended at least 70% of the supervised exercise sessions, and completed a mean total supervised exercise time of at least 40 min.

#### 2.12.2 Primary analysis

The ADAS‐Cog‐Exec is a weighted change score from baseline resulting in one score per participant. This score was analyzed using linear regression to test differences between interventions groups in the mITT population, with type I error level set at 0.05. The linear regression model included terms for intervention assignment plus covariates consisting of site, *APOE* ε4 carrier status, and sex. Potential confounders (baseline Walk Test time, age, education, 3MSE baseline score, use of cholinesterase inhibitors) were considered as covariates if the following two conditions were satisfied: (1) imbalance at baseline (*p* < 0.1) and (2) association between covariate and response (*p* < 0.15). The primary analysis compared group changes from baseline to the mean score obtained across the month 6 and month 12 assessments. We made the a priori decision to use the mean value across two timepoints to stabilize scores in light of evidence showing cognitive benefit with 6 months of exercise in other RCTs.^25^, ^26^, ^51^ When month 6 or month 12 data were missing, the score obtained at the available assessment visit was used. If an ADAS‐Cog‐Exec component score was missing or not administered due to cognitive issues (i.e., participant unable to complete), the worst possible test score was assigned prior to construction of the composite. Safety data were analyzed using exact contingency table methods by intervention group assignment.

#### 2.12.3 Other analyses

Longitudinal change in episodic memory and executive function scores from baseline through month 12 in the mITT, pre‐pandemic, and per‐protocol (PP) populations were analyzed to test group differences using a strategy similar to that described for the primary outcome (i.e., linear regression, pre‐specified covariates: site, *APOE* ε4 carrier status, sex), with type I error level set at 0.05. Analyses of other secondary and exploratory outcomes were conducted using a similar approach. No type 1 error adjustments were made for secondary, exploratory, or sensitivity analyses. For more information, see the protocol and Statistical Analysis Plan (Supplement).

## 3 RESULTS

**FIGURE 2:**
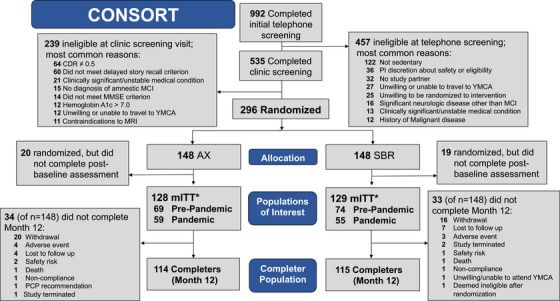
Consolidated Standards of Reporting Trials (CONSORT) flow diagram.

The CONSORT figure (Consolidated Standards of Reporting Trials) is provided in Figure 2. Screening was initiated in July 2016. A total of 992 individuals completed initial telephone screening to assess eligibility, 535 advanced to screening in the clinic, and 296 were randomized. Participants were enrolled from September 2016 to February 2020. The most common reasons for telephone screen failure included not sedentary (27%), safety issues or use of ineligible medications (8%), and no study partner (7%). The last participant exited the study in October 2021, 9 months later than the expected end date due to COVID‐19 pandemic‐related disruption. Baseline characteristics (Table 1) and baseline cognitive test scores (Table 2) were balanced across treatment groups. Twenty‐five percent of the sample were *APOE* ε4 carriers, consistent with other reports based on samples recruited from the community rather than from health care (memory) clinics.^52^, ^53^ EXERT baseline AD biomarker Aβ42/Aβ40 levels in blood and CSF (Table S1) were also consistent with those reported in other studies of community‐dwelling adults with mild memory loss associated with early MCI.^52^, ^53^ The cohort included 15% racial and ethnic minoritized groups. Of randomized participants, 257 (87%) initiated the intervention and completed at least one post‐baseline assessment (mITT), and 229 (77.4%) completed the 12‐month assessment. Baseline characteristics of the mITT population were similar to those of the entire cohort (see Table S2). The most common reasons randomized participants failed to initiate the intervention included new hesitancy to commit to the 18‐month intervention or to travel to the YMCA three to four times per week, and new medical conditions or injuries precluding commitment to regular exercise. Of the mITT group (*n* = 257), 143 (56%) completed the supervised phase of the study (month 12) prior to initiation of the pandemic‐related study pause in March 2020, and 24 (≈10%) completed the baseline and month 12 lumbar punctures. Reasons for study non‐completion are summarized in Figure 2.

**TABLE 1:** Baseline characteristics of EXERT cohort.

Variable	Total (*N* = 296)	AX (*n* = 148)	SBR (*n* = 148)	*p*
Age, mean years (SD)	74.5 (6.0)	74.3 (5.7)	74.7 (6.2)	0.76
Sex, no. female (%)	169 (57.1%)	85 (57.4%)	84 (56.8%)	>0.99
MMSE, mean score (SD)	28 (1.9)	27.9 (1.9)	28.0 (1.8)	0.62
Education, mean years (SD)	16.2 (2.4)	16.2 (2.4)	16.3 (2.4)	0.87
*APOE* genotype, no. ε4 carrier (%)	74 (25.0%)	37 (25.0%)	37 (25.0%)	1.00
Race, no. (%)				0.94
American Indian, Alaska Native	3 (1.0%)	2 (1.4%)	1 (0.7%)	
Asian	7 (2.4%)	3 (2.0%)	4 (2.7%)	
Native Hawaiian, Pacific Islander	0 (0.0%)	0 (0.0%)	0 (0.0%)	
Black, African American	29 (9.8%)	15 (10.1%)	14 (9.5%)	
White	256 (86.5%)	127 (85.8%)	129 (87.2%)	
Unknown or not reported	0 (0.0%)	0 (0.0%)	0 (0.0%)	
Multiple races	1 (0.3%)	1 (0.7%)	0 (0.0%)	
Ethnicity, no. (%)				0.41
Hispanic, Latino	3 (1.0%)	3 (2.0%)	0 (0.0%)	
Not Hispanic or Latino	287 (97.0%)	142 (95.9%)	145 (98.0%)	
Unknown, not reported	6 (2.0%)	3 (2.0%)	3 (2.0%)	
Marital status, no. (%)				0.99
Married	185 (62.5%)	92 (62.2%)	93 (62.8%)	
Widowed	33 (11.2%)	16 (10.8%)	17 (11.5%)	
Divorced	63 (21.3%)	33 (22.3%)	30 (20.3%)	
Never married	13 (4.4%)	6 (4.1%)	7 (4.7%)	
Unknown, Other	2 (0.7%)	1 (0.7%)	1 (0.7%)	
Retired, no. (%)				0.89
No	63 (21.3%)	30 (20.3%)	33 (22.3%)	
Yes	231 (78.0%)	117 (79.1%)	114 (77.0%)	
Not applicable	2 (0.7%)	1 (0.7%)	1 (0.7%)	
Home, no. (%)				0.58
Independent living facility	286 (96.6%)	142 (96.0%)	144 (97.3%)	
Lives with family	7 (2.4%)	5 (3.4%)	2 (1.4%)	
Senior residence	3 (1.0%)	1 (0.7%)	2 (1.4%)	
Assisted living	0 (0.0%)	0 (0.0%)	0 (0.0%)	
Skilled nursing facility	0 (0.0%)	0 (0.0%)	0 (0.0%)	
Other	0 (0.0%)	0 (0.0%)	0 (0.0%)	

Abbreviation: APOE, apolipoprotein; MMSE, Mini‐Mental Status Exam.

**TABLE 2:** Baseline cognitive test scores.

Test	Total (*N* = 250)	AX (*n* = 124)	SBR (*n* = 126)	*p*
ADAS‐Cog13	13.8 (6.5)	13.6 (6.4)	14.0 (6.7)	0.80
ADAS‐Cog13 Word List Recall^a^	3.7 (1.5)	3.7 (1.6)	3.7 (1.5)	0.84
ADAS‐Cog13 Delayed Word List Recall	4.1 (2.5)	4.1 (2.4)	4.2 (2.6)	0.64
ADAS‐Cog13 Orientation	0.3 (0.6)	0.3 (0.5)	0.3 (0.6)	0.81
ADAS‐Cog13 Number Cancellation	1.7 (1.0)	1.7 (0.9)	1.7 (1.0)	0.93
CDR‐Sum of Boxes scores (Memory)	0.5 (0.2)	0.5 (0.2)	0.5 (0.2)	0.50
CDR‐Sum of Boxes scores (Orientation)	0.3 (0.3)	0.2 (0.3)	0.3 (0.3)	0.91
CDR‐Sum of Boxes scores (Judgment & Problem Solving)	0.4 (0.3)	0.4 (0.3)	0.4 (0.3)	0.23
Trails A, time	38.0 (13.9)	38.3 (14.3)	37.8 (13.5)	0.98
Trails B, time	101.0 (53.5)	103.0 (55.1)	100.0 (52.0)	0.85
Digit Symbol Substitution	44.6 (10.4)	44.6 (10.6)	44.6 (10.3)	0.98
Category Fluency^b^	16.0 (4.1)	15.8 (4.1)	16.2 (4.2)	0.37
Letter Fluency^c^	12.7 (4.0)	12.7 (3.9)	12.8 (4.1)	0.98

Sufficient baseline data for construction of the primary cognitive composite score were available for *n* = 250 participants.

^a^ Mean across three trials.

^b^ Mean of Animals & Vegetables.

^c^ Mean of F & L.

### 3.1 Intervention adherence

**FIGURE 3:**
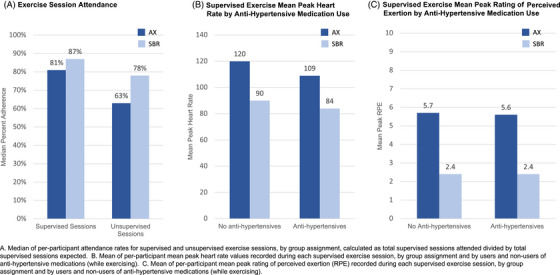
Intervention adherence by exercise session attendance (A), supervised exercise mean peak heart rate by anti‐hypertensive medication use (B)*, and supervised exercise mean peak rating of perceived exertion by anti‐hypertensive medication use (C)*.

During the first 12 months of the trial (supervised phase of the intervention), participants collectively completed 31,943 exercise sessions (18,045 supervised sessions; 13,898 unsupervised sessions). The number of completed supervised sessions was comparable across groups, exceeding 80% overall with 81% attendance for the AX group and 87% attendance for the SBR group. Intervention adherence metrics are provided in Figure 3. Attendance of the unsupervised exercise sessions through month 12 was lower for both groups, particularly for AX participants. Mean HR and RPE during supervised exercise were higher for the AX group than for the SBR group. Pandemic‐related study pause weekly calls to participants (8451 weeks for 105 participants, 97% call completion rate) indicated that the self‐reported mean (SD) number of weekly exercise sessions completed was 3.16 (1.95), which did not differ across intervention groups (*p *> 0.05).

### 3.2 Primary outcome

**FIGURE 4:**
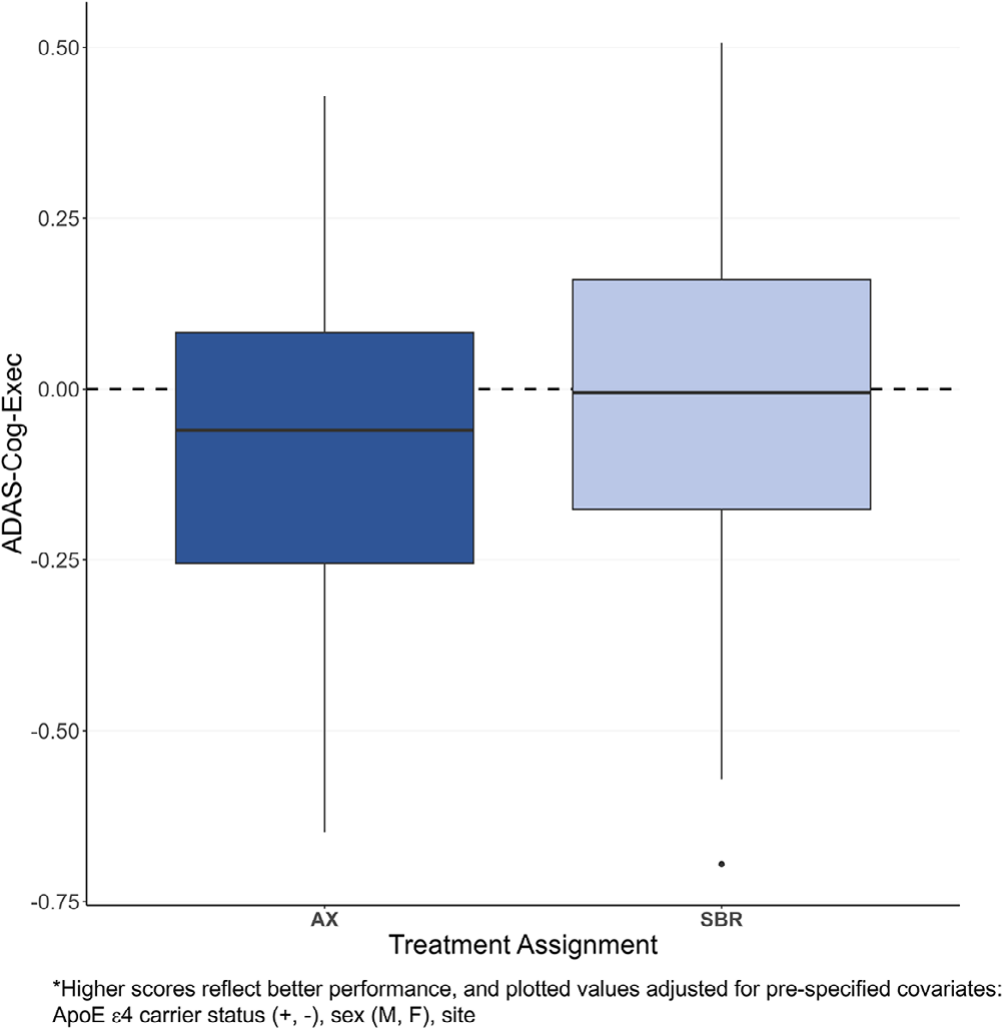
Mean change across months 6 and 12 relative to baseline on the ADAS‐Cog‐Exec Global Cognitive Composite Score*.

Figure 4 plots mean change (and SD) in the ADAS‐Cog‐Exec across months 6 and 12 (averaged) relative to baseline by treatment group assignment in the mITT population, adjusted for pre‐specified covariates (site, *APOE* ε4 carrier status, sex; no other potential confounders met pre‐specified conditions). Mean change scores did not differ significantly between groups (–0.078, SE = 0.074, *p *= 0.3; see Table S3 for model summary). Notably, ADAS‐Cog‐Exec scores remained stable over the 12‐month period (no significant decline from baseline) for both AX (0.146; standard error [SE] = 0.063; 95% confidence interval [CI]: 0.021–0.27) and SBR (0.068; SE = 0.063; 95% CI: –0.057 to 0.19).

### 3.3 Secondary and exploratory outcomes

Mean change scores across months 6 and 12 (relative to baseline) for domain‐specific cognitive composite scores (described in Supplement, Protocol) did not differ by intervention group (executive function: –0.009, SE = 0.04, *p = *0.8; episodic memory: –0.02, SE = 0.05, *p = *0.7). Mean change scores also did not differ by group for CDR‐SB (*p = *0.6), ADAS‐Cog13 (*p = *0.9), ADCS‐ADL‐MCI (*p = *0.4), CCI (*p = *0.2), BRIEF‐A (*p = *0.3), or NPI (*p = *0.2). In sensitivity analyses exploring additional time effects on intervention response, there were no group differences from baseline to month 6, or from baseline to month 12 (*p *> 0.2) for any of the cognitive or functional outcomes assessed. Brain imaging showed no 12‐month group differences in atrophy or blood flow in the hippocampus, prefrontal, or AD signature composite regions, or in whole brain or ventricular volume (Table S4). Notably, 12‐month mean (SD) hippocampal volume loss was 0.51% (1.08, *n* = 187) across both groups, which did not differ from each other: AX –0.67% (1.16, *n* = 92); SBR –0.37% (0.99, *n* = 95). There were no group differences in AD biomarkers in blood (Aβ42, Aβ40) or CSF (BDNF, t‐tau, p‐tau181) (Table S1); notably, Aβ42/Aβ40 levels in blood and CSF remained stable over the 12‐month regardless of intervention group assignment (unadjusted means for AX and SBR in blood, baseline: 0.04, month 12: 0.04; and in CSF, baseline: 0.07, month 12: 0.07). Both groups showed improvement on the self‐report EQ‐5D quality of life scale (AX: 0.65, 95% CI 0.33–0.97; SBR: 0.41, 95% CI 0.10–0.72). AX showed more improvement than SBR on the SF‐36 Health Change Scale (–6.0, SE = 2.8, *p *= 0.03). Similar findings as those described above for all outcomes were obtained in pre‐specified participant sub‐groups (i.e., pre‐pandemic, pandemic, per‐protocol). Post hoc sensitivity analyses were conducted to assess whether participants with relatively more baseline cognitive impairment responded similarly to the intervention compared to other participants. Greater cognitive impairment was identified in two ways: (1) lowest baseline 3MSE tertile, and (2) whether more stringent cognitive eligibility criteria used early in the trial were met. Regardless of how cognitive status was defined, there was no indication that individuals with greater baseline cognitive impairment responded differently to the intervention (two‐way interactions: impairment classification × treatment, *p *> 0.5).

### 3.4 Safety

Overall, there were 655 AEs reported that did not differ significantly by group assignment (*p *= 0.80; AX: *n* = 344, 77% of participants experienced at least one AE; SBR: *n* = 311, 74% of participants experienced at least one AE). Severity of AEs was comparable across groups (mild: AX 61% vs SBR 62%; moderate: AX 32% vs SBR 30%; severe: AX 8% vs SBR 8%). Relatedness of AEs to the intervention was also comparable across groups (definitely: AX 1 .5% vs SBR 1.3%; probably: AX 4 .7% vs SBR 2.3%; possibly: AX 14% vs 18%; unrelated: AX 65% vs SBR 63%). The most common AEs reported (of 655 in total, Medical Dictionary for Regulatory Activities [MedDRA] coding) included musculoskeletal and connective tissue disorders (AX *n* = 78, SBR *n* = 70), injury, poisoning and procedural complications (AX n = 76, SBR *n* = 59), and infections and infestations (AX *n* = 60, SBR *n* = 51).

Number of SAEs reported did not differ by intervention group (*p *= 0.87; AX *n* = 23, 14% of participants experienced at least one SAE; SBR *n* = 23, 11% of participants experienced at least one SAE). Severity of SAEs was similar across groups (mild: AX 0% vs SBR 9%; moderate: AX 43% vs SBR 26%; severe: AX 57% vs SBR 65%), as was Relatedness (definitely or probably: AX 0% vs SBR 0%; possibly: AX 8 .7% vs SBR 0%; unlikely: AX 4 .4% vs SBR 43%; unrelated: AX 74% vs SBR 57%). The most common SAEs recorded (MedDRA coding) included infections and infestations (AX *n* = 5, SBR *n* = 4), surgical and medical procedures (AX *n* = 4, SBR *n* = 4), and cardiac disorders (AX *n* = 4, SBR *n* = 3). At no time did the DSMB recommend stopping the trial for safety or other reasons. Vital signs by group assignment and overall are provided in Table S5. The final DSMB report, including vital signs by group assignment, is provided as a Supplement.

## 4 DISCUSSION

Twelve months of supervised moderate‐high intensity aerobic versus low‐intensity stretching/balance/range of motion exercise in sedentary older adults with amnestic MCI did not differentially affect global cognitive function as measured by the ADAS‐Cog‐Exec. In addition, there were no differences by intervention group on the CDR‐SB or ADAS‐Cog13, or on self‐ or study partner–assessed measures of functional status, executive function, mood, or health and quality of life. There were also no 12‐month intervention group differences on pre‐specified regional and whole brain volumes or blood flow, or on AD specimen biomarkers. However, neither the AX nor the SBR group showed a 12‐month decline as might be expected for adults with amnestic MCI.^54^

### 4.1 Cognitive protection for both intervention groups versus healthy participant bias

Global cognition was measured using a composite score developed and validated with high sensitivity to 12‐month change in adults with amnestic MCI^37^; yet no 12‐month change was observed for either intervention group. In the validation study, the mean to SD ratio (MSDR) for 12‐month change in ADAS‐Cog‐Exec scores for Alzheimer's Disease Neuroimaging Initiative (ADNI1) participants (matched to EXERT baseline characteristics) was 0.48. In EXERT, MSDR for change in the primary outcome (ADAS‐Cog‐Exec mean at months 6 and 12) was less than 0.15 for both groups. However, without a passive control comparator (no intervention), we are unable to confirm that EXERT exercise protects against cognitive decline. Cognitive benefits of multi‐component low and high‐intensity exercise (e.g., balance, flexibility, resistance, and aerobic) have been reported by others. A 2024 meta‐analysis^55^ of 1393 participants with MCI across 20 RCTs indicated greater cognitive benefit with multi‐component exercise versus aerobic training alone when completed for a minimum of 4 months at the same frequency and session duration used in EXERT. Another meta‐analysis^56^ of 39 non‐pharmacological intervention trials in older adults with subjective cognitive decline concluded that aerobic, resistance, and balance exercises all show independent benefits on global cognition, with balance exercises showing the most potent effects. In a 1‐year RCT of aerobic exercise versus stretching/toning in 73 cognitively healthy older adults,^57^ both groups showed improvement in global cognition, but, as in EXERT, the benefit did not differ across intervention groups. In this sample, 12‐month hippocampal volume loss was 0.81%—consistent with rates reported in other studies of healthy aging^4^, ^25^—which was greater than what we observed in EXERT participants with MCI (0.51%). Other studies of adults with MCI have also shown higher annual rates of hippocampal volume loss even among those classified as cognitively stable.^58^, ^59^ Relative preserved hippocampal volume with exercise in MCI aligns with meta‐analyses of RCTs, indicating a moderate protective effect.^60^ These and other reports,^35^, ^61^ including those of RCTs in MCI,^62^ point to the possibility that cognitive benefit for adults with MCI might be agnostic to type of exercise and more dependent on the regularity with which at least low‐medium intensity exercise is completed.

A second account of our findings to be considered is that the exercise interventions had no effect on cognition and that the absence of cognitive decline we observed may reflect a healthy participant selection bias in EXERT. In the planning of EXERT, we used several strategies to address recruitment and study design factors that have been linked to increased selection bias in clinical trials due to low trust of research by the community, use of recruitment approaches that largely exclude underserved and underrepresented individuals, and study designs that disregard participant wishes and needs.^63^ In EXERT, we developed culturally sensitive recruitment materials, used targeted grassroots outreach focused on developing mutually beneficial community partnerships to support the trial, and cast a wide net to identify potential study candidates beyond referrals provided through a health care network.^36^ In addition, the interventions were delivered at local YMCAs and by YMCA trainers to increase community support and participation, we obtained guidance from community focus groups to inform study design (i.e., no usual care group), and participants were incentivized with free 18‐month access to the YMCA and monetary compensation for their time. As a consequence, the EXERT cohort was more diverse than is typical of other AD trials,^64^ with 15% from minoritized racial and ethnic groups, and baseline participant characteristics indicated suboptimum health status (sedentary, hypertensive, overweight; see Table S5). Our recruitment strategies and protocols were designed to enroll adults with MCI with characteristics that largely reflect those of older Americans with respect to sedentary behavior and medical comorbidities, factors that have not been associated with resilience to cognitive decline.

### 4.2 Large amount of exercise completed by participants

EXERT is the largest multisite trial to date examining the effects of rigorous structured and supervised exercise completed by sedentary older adults with amnestic MCI for at least 12 months. Our findings conflict with those of other studies showing benefit of aerobic over flexibility/balance exercise in MCI, which often include fewer participants, shorter intervention durations (≤ 6 months), or home‐based exercise with minimal oversight.^25^, ^26^, ^40^, ^62^, ^65^, ^66^, ^67^ YMCA trainers supervised AX and SBR participants for 12 months during which participant progress was reviewed, barriers were identified and addressed, and adherence was encouraged. Overall adherence to both interventions was high (>80%), even during the pandemic‐related study pause, with more than 31,000 exercise sessions completed across participants in the supervised phase of the trial. Participation increased basal rates of physical activity, regardless of randomization assignment; participants traveled to the YMCA three to four times per week to complete 30–45 min of exercise each time, providing an activity boost of at least 150 h per year, or about 12 h per month for once‐sedentary individuals. The absence of cognitive decline over 12 months may be linked to the overall increase in physical activity in both intervention groups.^68^ Of note, the SBR group attended more exercise sessions than the AX group, which could have conferred additional protection for these participants.

### 4.3 Sufficient separation in exercise intensity between intervention groups

The study was designed to control total amount of exercise (frequency, duration) and quantity of trainer support to isolate potential effects of exercise intensity on cognitive trajectory. Exercise prescriptions identified specific targets for mean and peak HR and RPE, which were largely achieved by both groups (Figure 3). Mean peak HR during exercise differed across groups by 30 bpm (for non‐users of anti‐hypertensives). One possibility to consider is that the absence of a group effect on cognitive trajectory relates to insufficient separation of achieved exercise intensity. In the AX group, participants (on average) exercised at ≈65%–80% HRR. The SBR group was coached to exercise at 30%–35% HRR, which was still ≈30% higher than pre‐study basal state for physical activity. Inclusion of a usual care control group might have helped address the issue of whether more exercise (of any type) versus no exercise leads to cognitive benefit. However, physical activity RCT designs that include usual care also introduce other issues that can be problematic for data interpretation (e.g., effect on willingness to enroll and stay in the study, intervention group differences in social support and/or mental stimulation). EXERT did not include a randomized usual care group, which was largely informed by: (1) feedback from community focus groups discouraging use of a no‐intervention group to foster successful recruitment and retention, (2) an ethical obligation to provide active comparison groups due to well‐accepted exercise‐related health benefits, and (3) budget constraints. Our pre‐specified alternate approach was to leverage extensive natural history data in studies with harmonized cognitive outcomes to estimate usual care effects, which will be described in a separate study. Notably, our inclusion of a low‐intensity SBR group as the active control provides important information that could inform the development of a feasible and sustainable exercise program for the wider community. Low‐intensity SBR exercise requires minimal specialized equipment, can be completed anywhere, is safe and beneficial for balance issues, and is more achievable for older adults.

### 4.4 Regular social (trainer) support was a key component of both interventions

EXERT interventions provided equal amounts of trainer support for all participants, which may have impacted our findings. The importance of regular social contact for brain health has received much attention in the past few years, in large part due to the impact of the COVID‐19 pandemic on social isolation—particularly among older adults.^69^ EXERT provided structured social contact twice per week to participants in both groups for 12 months (≈100 h/year), and this amount has been linked to a reduced risk of cognitive decline for adults with MCI in a large longitudinal population‐based study.^70^ RCTs of structured conversational interaction in adults at high risk for cognitive decline report improved cognitive function with regular social contact.^71^, ^72^ Given the volume (frequency, duration) of exercise completed by both groups and the previously reported benefits of regular physical activity on brain health (reviewed^55^, ^73^), we hypothesize that both groups remained cognitively stable over 12 months due to regular prolonged exposure both to increased physical activity and to increased social stimulation. EXERT data, however, do not permit us to infer a causal relationship, or to disentangle potential separate contributions of these intervention components on 12‐month cognitive trajectory.

### 4.5 COVID‐19 pandemic impact on intervention delivery and response

At the time of the study‐wide pause, the trial was fully enrolled and nearly 50% of the cohort had completed the 12‐month outcomes assessment. The study team completed weekly telephone visits with all active participants to provide support, and to collect self‐report exercise data that documented continued high adherence for a large proportion of individuals across both intervention groups. Although there were no 12‐month group differences for the pre‐pandemic or pandemic subgroups on the ADAS‐Cog13, scores tended to worsen for participants who completed the intervention during the pandemic [pre‐pandemic AX mean change standard error of the mean (SEM): –0.063 (0.50); pandemic AX mean change (SEM): 0.89 (0.59); pre‐pandemic SBR mean change (SEM): –0.13 (0.48); pandemic SBR mean change (SEM): 0.98 (0.61)], and this impact may have affected our results. EXERT, however, is not powered to statistically test for differences across pandemic‐related study epochs.

### 4.6 Strengths and limitations

#### 4.6.1 Strengths

EXERT was the largest multisite randomized clinical trial of high‐ versus low‐intensity exercise in adults with amnestic MCI conducted to date. The multisite component captured regional variability in lifestyle practices, risk factor prevalence, screening/assessment heterogeneity, and pandemic impact. EXERT recruitment strategies were innovative and diverse^36^ and instrumental to successful enrollment of 15% from minoritized ethnic groups. The trial's rigor ensured careful delivery and oversight of the intervention to ensure maximum uptake by participants at the prescribed dose with over 31,000 exercise sessions completed by 296 once‐sedentary individuals with MCI, which is a notable accomplishment in itself. EXERT partnered with the Y‐USA to implement the interventions in community YMCA branches, which enhanced external validity and translatability of the design. YMCA expertise was leveraged for intervention delivery and behavior change, and clinical site and Coordinating Center guidance was provided to educate trainers about living with MCI. EXERT developed, validated, and used a new composite measure of global cognition that adds executive function tasks and clinical assessments that are most sensitive to cognitive decline in MCI. EXERT also tested new methods of exercise delivery in the context of a pandemic, with lessons learned to inform the development of other physical activity trials and community‐based exercise programs.

#### 4.6.2 Limitations

EXERT did not include a randomized usual care control group for reasons described above. By design, regular social contact was an integral part of intervention delivery for both intervention groups so that cardiorespiratory intensity effects could be isolated and compared. As a result, the unique contribution of social support cannot be examined in EXERT. The gold standard measure of cardiorespiratory fitness (VO_2_ max) was not collected and therefore can be assessed relative to cognitive response. Trial conduct during a pandemic was not only a strength but also a limitation, as it impacted how and whether the intervention could be completed by participants at the prescribed dose. Finally, the eligibility criteria were altered early in the recruitment phase to include candidates identified through a wider‐net approach rather than solely through memory clinics; sensitivity analyses indicated that this change, however, did not impact EXERT findings.

## 5 SUMMARY

EXERT trial conduct was highly successful, as indicated by our ability to recruit the targeted number of individuals with MCI, and to achieve high intervention adherence and low attrition—even during a pandemic. Our findings indicate that regular higher intensity aerobic or lower‐intensity flexibility exercise for 120–150 min per week for 12 months with regular social support did not differentially affect global cognitive function. However, neither exercise intervention group showed 12‐month cognitive declines that might be expected for adults with amnestic MCI, an association that warrants further study.

## AUTHOR CONTRIBUTIONS

*Concept and design*: Baker, Cotman, Katula, Lawson, Chmelo, Nicklas, Brewer, Thomas, Rissman, Jung, Evans, Aisen, Feldman. *Acquisition, analysis, or interpretation of data*: Baker, Cotman, Katula, Lawson, Hodge, Chmelo, Nicklas, Johnson, Brewer, Rissman, Jung, Aslanyan, Salmon, Jacobs, Morrison, Matthews, Taylor, Leger, Messer, Evans, Okonkwo, Shadyab, La Croix, Mason, Thomas, Jin, Zhang, Pa, Feldman. *Statistical analysis*: Thomas, Jin, Zhang, Zou, Aslanyan, Pa. *Manuscript development*: Baker, Pa, Katula, Salmon, Jacobs, Aslanyan, Hodge, Chmelo, Morrison, Brewer, Rissman, Shadyab, Evans, Okonkwo, Zou, La Croix, Feldman.

## CONFLICT OF INTEREST STATEMENT

Feldman receives grant funding from the National Institute on Aging (U19AG010483‐22), from Biohaven Pharmaceuticals, Vivoryon (Probiodrug), and LuMind Foundation; service agreements for consulting activities with LuMind, Axon Neuroscience, Novo Nordisk, Arrowhead Pharmaceuticals, Roche/Genentech Pharmaceuticals (DMC/DSMB), Tau Consortium (SAB), and Janssen Research & Development (DSMB); support for travel from Novo Nordisk, Royal Society of Canada, Translating Research in Elder Care (TREC), Association for Frontotemporal Dementia (AFTD), and Rainwater Charitable Foundation; and a philanthropic donation for the Epstein Family Alzheimer Research Collaboration. No personal funds have been received for these activities. Feldman personally receives royalties for patent: Feldman HH (filed November 26, 2008). Detecting and Treating Dementia Serial Number 12/3‐2691 U.S. Patent No. PCT/US2007/07008. Washington, DC: U.S. Patent and Trademark Office. For other authors, there are no conflicts to report for this work. Author disclosures are available in the Supporting Information.

## Supporting information



Supporting Information

Supporting Information

Supporting Information

Supporting Information

Supporting Information

Supporting Information

## Collaborators: EXERT Study Group

Marwan Sabbagh, MD and Charles Bernick, MD, MPH (Cleveland Clinic Lou Ruvo Center for Brain Health); Kathleen Welsh‐Bohmer, PhD (Duke University Medical Center); Whitney Wharton, PhD and Joseph Nocera, PhD (Emory University); Steve Satek, MBA, Jeffrey Ross, MD and Linda Rice, PhD (Great Lakes Clinical Trials); Jeffrey Burns, MD, MS (Kansas University, Kansas City); Clara Li, PhD and Mary Sano, PhD (Mt. Sinai School of Medicine); Martin Sadowski, MD, PhD and Steven Ferris, PhD (New York University Medical Center); J. Kaci Fairchild, PhD and Jerome Yesavage, MD (Stanford University School of Medicine); Steven Tam, MD and Aimee Pierce, MD (University of California, Irvine); Allison Caban‐Holt, PhD and Shoshana Bardach, PhD (University of Kentucky, Lexington); Sid O'Bryant, PhD and Leigh Johnson, PhD (University of North Texas Health Science Center); Christopher Van Dyck, MD (Yale University School of Medicine).
