# Supplementary material for: Effects of exercise on cognition and Alzheimer's biomarkers in a randomized controlled trial of adults with mild cognitive impairment: The EXERT study
Source: Alzheimers Dement. 2025 Apr 24;21(4):e14586. doi: 10.1002/alz.14586 (PMC12019696; doi:10.1002/alz.14586)
Supplement: Supplementary file 1 — Supporting Information [file ALZ-21-e14586-s002.pdf]

Supplement Figure S1. Timeline of COVID-19 Pandemic Study Pause and Restart

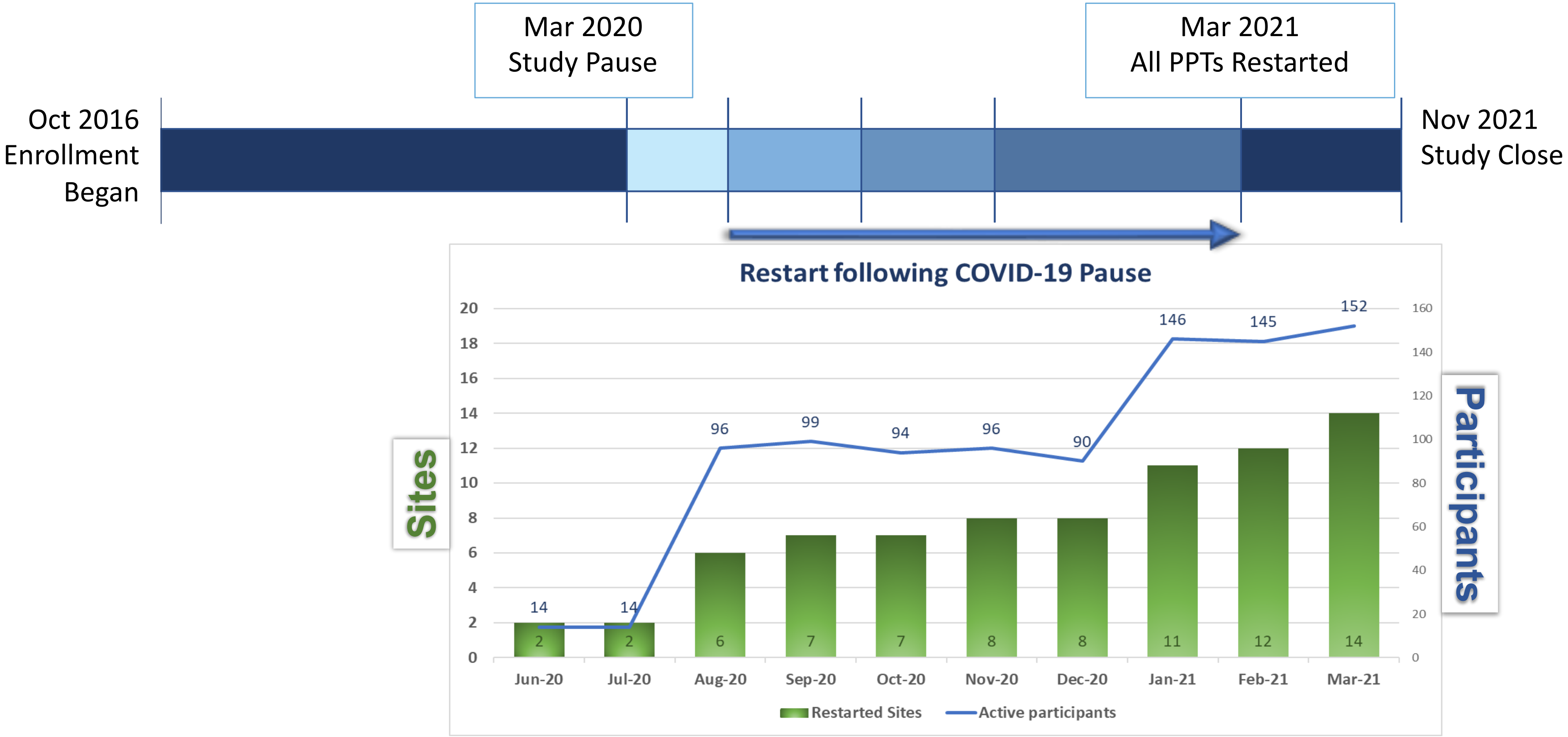

Supplement Table S1. Intervention Effects on AD Fluid Biomarkers

AD Specimen Biomarkers (pg/ml or ratio)		Unadjusted Baseline Values mean (SD)		Intervention Effect (SE)[a]	*p-value*
		AX	SBR		
**Plasma**	Aβ42b	6.71 (2.68)	7.04 (2.26)	-0.38 (0.36)[b]	0.29
	Aβ40	177.7 (66.1)	193.1 (60.5)	-7.56 (8.99)[c]	0.40
	Aβ42/Aβ40	0.04 (0.02)	0.04 (0.01)	-0.0005 (0.002)[b]	0.82
**CSF**	Aβ42	866.1 (534.9)	767.7 (322.4)	40.96 (103)[d]	0.70
	Aβ40	11949.4 (4526)	10918.3 (4204)	269 (1401)[d]	0.85
	Aβ42/Aβ40	0.07 (0.03)	0.07 (0.03)	0.002 (0.004)[d]	0.64
	Total Tau	428.1 (231.8)	357.0 (210.7)	25.94 (56.70)[d]	0.65
	pTau	63.3 (44.8)	51.8 (38.7)	4.63 (7.25)[d]	0.53
	Aβ42/Tau	2.6 (1.9)	2.9 (2.0)	-0.01 (0.33)[d]	0.97
	Aβ42/pTau	20.5 (15.8)	22.8 (16.4)	-0.17 (2.33)[d]	0.94
	BDNF	0.08 (0.03)	0.09 (0.08)	0.02 (0.04)[e]	0.66

pTau: phosphorylated tau protein; BDNF: brain-derived neurotrophic factor

[a]Effect: SBR-AX, Month12-Baseline, Covariates=age, sex, APOE-ε4 carrier status, baseline MMSE; [b](AX)=103, n(SBR)=111; [c]n(AX)=107, n(SBR)=112; [d]n(AX)=14, n(SBR)=10; [e]n(AX)=9, n(SBR)=5

Supplement Table S2. Baseline Characteristics of EXERT Modified Intent To Treat (mITT) Population

Variable	Total (N=250)*	AX (n=124)	SBR (n=126)	*p*-value
Age, yrs	74.5 (6.1)	74.5 (5.8)	74.5 (6.3)	0.79
Sex, no. Female (%)	136 (54.4%)	66 (53.2%)	70 (55.6%)	0.80
MMSE	28.1 (1.8)	28.0 (1.8)	28.1 (1.8)	0.46
Education, yrs	16.3 (2.3)	16.3 (2.3)	16.4 (2.3)	0.94
ApoE Genotype, no. E4 carrier (%)	62 (24.8%)	31 (25.0%)	31 (24.6%)	1.00
Race, no. (%)				0.63
American Indian, Alaskan Native	3 (1.0%)	2 (1.6%)	1 (0.8%)	
Asian	6 (2.4%)	2 (1.6%)	4 (3.2%)	
Native Hawaiian, Pacific Islander	0 (0.0%)	0 (0%)	0 (0.0%)	
Black, African American	20 (8.0%)	8 (6.5%)	12 (9.5%)	
White	220 (88.0%)	111 (89.5%)	109 (86.5%)	
Unknown or Not Reported	0 (0.0%)	0 (0.0%)	0 (0.0%)	
Multiple Races	1 (0.4%)	1 (0.8%)	0 (0.0%)	
Ethnicity, no. (%)				0.37
Hispanic, Latino	3 (1.2%)	3 (2.4%)	0 (0.0%)	
Not Hispanic or Latino	241 (96.4%)	118 (95.2%)	123 (97.6%)	
Unknown, Not Reported	6 (2.4%)	3 (2.4%)	3 (2.4%)	
Marital Status, no. (%)				0.92
Married	165 (66.0%)	83 (66.9%)	82 (65.1%)	
Widowed	23 (9.2%)	10 (8.1%)	13 (10.3%)	
Divorced	48 (19.2%)	25 (20.2%)	23 (18.3%)	
Never Married	12 (4.8%)	5 (4.0%)	7 (5.6%)	
Unknown, Other	2 (0.8%)	1 (0.8%)	1 (0.8%)	
Retired, no. (%)				0.82
No	54 (21.6%)	25 (20.2%)	29 (23.0%)	
Yes	194 (77.6%)	98 (79.0%)	96 (76.2%)	
Not Applicable	2 (0.8%)	1 (0.8%)	1 (0.8%)	
Home, no. (%)				0.45
Independent Living	243 (97.2%)	120 (96.8%)	123 (97.6%)	
Lives with family	6 (2.4%)	4 (3.2%)	2 (1.6%)	
Senior Residence	1 (0.4%)	0 (0.0%)	1 (0.8%)	
Assisted Living	0 (0.0%)	0 (0.0%)	0 (0.0%)	

*Baseline demographics for n=250 participants who contributed data for analysis of the primary endpoint (ADAS-Cog-Exec).

Supplement Table S3. Elements of the ADAS-Cog-Exec (Change from Baseline) and Primary Analysis Model Results for ADAS-Cog-Exec (Covariates: ApoE carrier status, sex, site)

variable	AX (n=128)	SBR (n=129)	Total (n=257)	p-value
word.recall-primary	-0.03±0.6 (128)	-0.14±0.71 (129)	-0.08±0.66 (257)	0.20
delayed.word.recall-primary	-0.02±0.53 (128)	-0.13±0.6 (129)	-0.08±0.57 (257)	0.11
adas.orientation-primary	0.09±0.85 (128)	0.28±1.13 (129)	0.18±1 (257)	0.14
adas.number.cancellation-primary	-0.03±0.77 (128)	0.03±0.88 (129)	0±0.83 (257)	0.60
memory-primary	0.03±1.29 (127)	-0.04±1.7 (128)	-0.01±1.51 (255)	0.70
orient-primary	0.12±1.12 (127)	0.03±1.16 (128)	0.08±1.14 (255)	0.50
judge-primary	0.1±1.05 (127)	-0.02±1.17 (128)	0.04±1.11 (255)	0.40
traascor-primary	0.1±0.83 (127)	-0.06±0.89 (129)	0.02±0.86 (256)	0.16
trabscor-primary	0.11±0.79 (125)	0.06±0.66 (127)	0.08±0.73 (252)	0.62
digittotal-primary	0±0.45 (128)	-0.04±0.5 (129)	-0.02±0.48 (257)	0.57
catflu-primary	-0.02±0.62 (128)	0.1±0.55 (129)	0.04±0.59 (257)	0.09
adas-cog-exec	0.07±0.56 (124)	0±0.67 (126)	0.03±0.61 (250)	0.32

tx	lsmean	SE	df	lower.CL	upper.CL
AX	0.146	0.063	233	0.021	0.27
SBR	0.068	0.063	233	-0.057	0.19

contrast	estimate	SE	df	t.ratio	p.value
SBR - AX	-0.078	0.074	233	-1.1	0.29

Abbreviations. word.recall-primary: ADAS-Cog13 word recall score; delayed.word.recall-primary: ADAS-Cog13 delayed word recall score; adas.orientation-primary: ADAS-Cog13 orientation score; adas.number.cancellation-primary: ADAS-Cog13 number cancellation number correct; memory-primary: Clinical Dementia Rating Scale (CDR) memory box score; orient-primary: CDR orientation box score; judge-primary: CDR judgement & problem-solving box score; traascor-primary: Trails A time to complete; trabscor-primary: Trails B time to complete; digittotal-primary: digit-symbol substitution number correct; catflu-primary: category verbal fluency, mean of animals and vegetables; adas-cog-exec: ADAS-Cog-Exec composite score (primary outcome); AX: Aerobic group; SBR: Stretching Balance Range of Motion group; lsmean: least squares mean (adjusted for covariates); SE: standard error; df: degrees of freedom; lower.CL: confidence interval lower limit; upper.CL: confidence interval upper limit.

Supplement Table S4. Intervention Effects on Brain Imaging Outcomes

MRI Outcomes		Intervention Effect (SE)[a]	*p-value*
**Volume**[b]	Hippocampal	0.28 (0.16)	0.08
	AD Signature Composite	-0.11 (0.14)	0.43
	Prefrontal Composite	-0.19 (0.17)	0.27
	Entorhinal	-0.09 (0.19)	0.65
	Ventricular	-0.03 (0.59)	0.96
	Whole Brain	-0.11 (0.10)	0.26
**Perfusion**	Hippocampal[c]	-0.03 (0.04)	0.5
	AD Signature Composite[c]	-0.05 (0.04)	0.23
	Prefrontal Composite[c]	-0.02 (0.05)	0.68
	Whole Brain[d]	-4.38 (3.62)	0.23
	Whole Brain - Gray Matter[d]	-2.90 (2.48)	0.24
	Whole Brain - White Matter[d]	-1.49 (1.31)	0.26

[a]Effect: SBR-AX, Month12-Baseline, Covariates=age, sex, APOE ε4 carrier status, baseline MMSE;
[b]n(AX)=92, n(SBR)=95; [c]n(AX)=74, n(SBR)=68; [d]n(AX)=78, n(SBR)=71

Supplement Table S5. Vital Signs for the Entire Sample and by Treatment Arm for (a) Weight, (b) Body Mass Index (BMI), (c) Systolic Blood Pressure, and (d) Diastolic Blood Pressure

a. **Weight (kg)**

	N	Mean	SD	Min	Q1	Median	Q3	Max	Pvalue
**sc2**									
AX	148	79.9	17.71	44.8	68	77.3	89.2	135	0.863
SBR	148	80.81	19	46.1	67.35	78.4	90.9	150.5	
Overall	296	80.36	18.34	44.8	67.57	77.55	90.1	150.5	
**bl**									
AX	147	79.53	17.59	44.6	68.2	76.7	89.15	135.9	0.618
SBR	147	81.03	18.87	47.1	67.7	77.5	91.15	150	
Overall	294	80.28	18.23	44.6	67.85	76.85	90.7	150	
**m06**									
AX	117	78.69	17.1	43.7	68.6	75.3	88.5	127.3	0.917
SBR	122	79.82	18.95	44.5	67.15	77.45	89.3	149.5	
Overall	239	79.27	18.04	43.7	67.3	76.8	89.1	149.5	
**m12**									
AX	111	78.98	17.09	43.2	69.5	78.8	89.7	128.2	0.934
SBR	113	80.19	19.08	45.9	65.9	78.2	89.1	150	
Overall	224	79.6	18.09	43.2	67.07	78.2	89.28	150	
**m18**									
AX	89	80.11	16.08	44.5	70.2	80.1	89.6	126.1	0.766
SBR	85	80.57	18.34	49.2	68.5	78.9	89.6	145.5	
Overall	174	80.34	17.18	44.5	68.95	79.35	89.6	145.5	

b. **BMI**

	N	Mean	SD	Min	Q1	Median	Q3	Max	Pvalue
**sc2**									
AX	148	28.65	5.63	18.76	24.97	27.6	31.86	48.06	0.832
SBR	147	29.05	5.95	18.56	24.5	28.15	31.79	51.58	
Overall	295	28.85	5.79	18.56	24.84	27.99	31.83	51.58	
**bl**									
AX	147	28.56	5.64	18.76	25.05	27.26	31.66	48.38	0.611
SBR	146	29.13	6.01	18.99	24.55	28.48	31.97	52.66	
Overall	293	28.85	5.83	18.76	24.98	28.03	31.74	52.66	
**m06**									
AX	117	28.04	5.38	17.92	24.43	27.57	30.94	45.38	0.624
SBR	121	28.65	5.79	18.36	24.09	28.06	31.44	47.29	
Overall	238	28.35	5.59	17.92	24.21	27.88	31.14	47.29	
**m12**									
AX	111	28.04	5.22	18.05	24.46	27.86	31.33	45.64	0.696
SBR	112	28.72	5.85	18.93	24.28	28.24	31.83	47.45	
Overall	223	28.38	5.54	18.05	24.27	28.03	31.56	47.45	
**m18**									
AX	89	28.32	4.94	19.38	25.04	27.73	31.44	43.28	0.774
SBR	85	28.69	5.38	20.09	23.95	28.53	32.05	44.26	
Overall	174	28.5	5.15	19.38	24.69	28.35	31.51	44.26	

c. **Systolic Blood Pressure (mm Hg)**

	N	Mean	SD	Min	Q1	Median	Q3	Max	Pvalue
**sc2**									
AX	148	136.04	15.39	100	124	134	147	194	0.143
SBR	148	139.8	20.05	91	127	138	150	214	
Overall	296	137.92	17.94	91	125	136	148	214	
**bl**									
AX	148	133.16	16.09	94	121	132	143.25	178	0.254
SBR	148	136.01	18.58	95	122	133	148	193	
Overall	296	134.59	17.41	94	122	132	145.25	193	
**m06**									
AX	117	133.72	16.89	92	122	132	144	181	0.256
SBR	123	137.2	20.22	96	123.5	134	149	200	
Overall	240	135.5	18.71	92	123	133	146	200	
**m12**									
AX	112	133.93	17.09	101	122	131.5	142	191	0.409
SBR	114	135.67	18.9	91	122	135	150.5	185	
Overall	226	134.81	18.01	91	122	133	146.75	191	
**m18**									
AX	90	131.82	16.36	80	120.25	130.5	144	171	0.364
SBR	85	135.52	19	98	121	133	149	183	
Overall	175	133.62	17.74	80	121	132	145	183	

d. **Diastolic Blood Pressure (mm Hg)**

	N	Mean	SD	Min	Q1	Median	Q3	Max	Pvalue
**sc2**									
AX	148	76.24	9.49	54	69.75	76	83	106	0.295
SBR	148	77.64	9.99	51	70	77.5	83	108	
Overall	296	76.94	9.75	51	70	77	83	108	
**bl**									
AX	148	75.05	9.17	49	70	76.5	81.25	96	0.404
SBR	148	74.82	9.96	49	69	74.5	80	104	
Overall	296	74.94	9.56	49	70	76	81	104	
**m06**									
AX	117	76.45	10.16	53	71	77	82	103	0.659
SBR	123	77.48	10.91	50	71	78	83	115	
Overall	240	76.98	10.54	50	71	77	83	115	
**m12**									
AX	112	75.57	9.88	45	69	75	81.25	102	0.46
SBR	114	76.32	10.19	53	70	76	82.75	105	
Overall	226	75.95	10.02	45	69	75	82	105	
**m18**									
AX	90	74.7	8.79	53	68.25	75	80.75	97	0.025
SBR	85	78.13	9.58	58	71	78	84	104	
Overall	175	76.37	9.32	53	69	77	83	104	

BMI: body mass index; sc2: Screening 2 (in clinic); bl: baseline; m06, m12, m18: month 6, 12, and 18 clinic visits); AX: aerobic training; SBR: stretching, balance, range of motion
