# Supplementary material for: Effects of exercise on cognition and Alzheimer's biomarkers in a randomized controlled trial of adults with mild cognitive impairment: The EXERT study
Source: Alzheimers Dement. 2025 Apr 24;21(4):e14586. doi: 10.1002/alz.14586 (PMC12019696; doi:10.1002/alz.14586)
Supplement: Supplementary file 3 — Supporting Information [file ALZ-21-e14586-s006.pdf]

Division of Biostatistics & Bioinformatics

Data Safety and Monitoring Board

Therapeutic Effects of Exercise in Adults with Amnestic Mild Cognitive Impairment (EXERT)

Blinded Armcode

CONFIDENTIAL DOCUMENT

March 17, 2022

For the ADCS Data Safety and Monitoring Board

the 22nd meeting for EXERT

Statistician: Jing Zhang, MSc

Unblinded Statistician: Karen Messer, PhD

Contents

1 Introduction 4

2 Executive Summary 4

3 Protocol Summary 5
3.1 Study Objectives 5
3.1.1 Primary Objective 5
3.1.2 Secondary Objectives 5
3.2 Power Analysis 5
3.3 Randomization Plan 5
3.4 Interim Analysis Plan 5
3.5 Extension Period 5

4 Methods 6
4.1 Data Set 6
4.2 Arm Code Assignment 6

5 Enrollment and Participant Status 7
5.1 Participant Status 7
5.2 Enrollment by Month 8
5.3 Enrollment by Randomization Strata 11
5.3.1 Enrollment by Site 11
5.3.2 Enrollment by Baseline ApoE4 carrier status 11

6 Visit Schedule Summary 11

7 Screen Fail 12
7.1 Screen Fail by Inclusion Criteria 12
7.2 Screen Fail by Exclusion Criteria 13

8 Early Discontinuation 14

9 Demographics 15

10 Vital Signs 18
10.1 Weight (kg) 19
10.2 BMI 20

10.3 Systolic Blood Pressure (mm Hg) . . . 21
10.4 Diastolic Blood Pressure (mm Hg) . . . 22
10.5 Pulse (bpm) . . . 23

11 Safety in the Main Study Period **26**
11.1 Adverse Events with Onset Before Randomization . . . 26
11.2 Adverse Events with Onset On or After Randomization . . . 26
11.2.1 AE Overall Summary . . . 26
11.2.2 AE by Severity . . . 27
11.2.3 AE by Relationship to Investigational Product . . . 27
11.2.4 Approved MedDRA Coded AEs . . . 28
11.3 Serious Adverse Events with Onset On or After Randomization . . . 37
11.3.1 SAE Overall Summary . . . 37
11.3.2 SAE by severity . . . 37
11.3.3 SAE by relationship to investigational product . . . 37
11.3.4 Approved MedDRA Coded SAEs . . . 38
11.4 Deaths . . . 42
11.5 Hospitalizations Reported as the Reason for a SAE . . . 43

12 Safety in the Extension Period **45**
12.1 Adverse Events in the Extension Period . . . 45
12.1.1 AE Overall Summary . . . 45
12.1.2 AE by Severity . . . 46
12.1.3 AE by Relationship to Investigational Product . . . 46
12.1.4 Approved MedDRA Coded AEs . . . 47
12.2 Serious Adverse Events with onset date in the extension period . . . 52
12.2.1 SAE Overall Summary . . . 52
12.2.2 SAE by severity . . . 52
12.2.3 SAE by relationship to investigational product . . . 52
12.2.4 Approved MedDRA Coded SAEs . . . 53
12.3 Deaths . . . 55
12.4 Hospitalizations Reported as the Reason for a SAE . . . 56

13 Protocol Deviations **57**

14 Appendix **58**
14.1 Listing of Inclusion/Exclusion Criteria (Protocol V2) . . . 58

CONFIDENTIALITY STATEMENT: The information contained in this document is confidential and may not be reproduced or otherwise distributed without the approval of the Biostatistics Research Center Directors.

14.2 Listing of Addendum Reports 60

15 Software 60

CONFIDENTIALITY STATEMENT: The information contained in this document is confidential and may not be reproduced or otherwise distributed without the approval of the Biostatistics Research Center Directors.

1 Introduction

This document presents the closed session safety report to the 22nd ADCS Data Safety and Monitoring Board (DSMB) meeting for the ADCS EXERT study using blinded arm code.

2 Executive Summary

This report is based on data downloaded on March 16th, 2022.

Enrollment and Participant Status Sections 5, 6, 7, and 8 provide information on the enrollment, visit schedule summary, screen fail, and early discontinuations. As of the data freeze, 986 subjects were screened, of which 690 were never randomized, 296 were randomized and 0 was pending. The enrollment was frozen since March, 2020. There were 63 early discontinuations.

Demographics Section 9 includes the demographic summary for all the randomized subjects.

Vital Signs Section 10 provides the descriptive summary for the vital signs.

Safety: Adverse Events (AEs), Serious Adverse Events (SAEs), Hospitalizations, and Deaths are reported in section 11 and 12. AEs are coded using the MedDRA dictionary and reported using the System Organ Class (SOC) and corresponding Preferred Term (PT). Detailed AE listings are provided in the Addendum Report. As of the data freeze, 799 (473 mild, 260 moderate, 66 severe) adverse events were reported with onset date on or after the randomization date for randomized subjects. 655 of them are in the main study period. During the main study period, 223 out of the 296 (75.3%) randomized subjects had at least one reported AE. There were 58 SAEs, 46 of them are in the main study period. 48 hospitalizations reported as the reason for a SAE, 38 of them are in the main study period. 4 death reported so far.

Protocol Deviations: Section 12 provides the summary of protocol deviations by type.

CONFIDENTIALITY STATEMENT: The information contained in this document is confidential and may not be reproduced or otherwise distributed without the approval of the Biostatistics Research Center Directors.

3 Protocol Summary

3.1 Study Objectives

3.1.1 Primary Objective

To evaluate the efficacy of aerobic exercise as measured by ADAS-Cog13 and ADAS-Cog-Exec.

3.1.2 Secondary Objectives

- To evaluate the efficacy of aerobic exercise as measured by Clinical Dementia Rating, mood and health-related quality of life, brain volume and perfusion.

3.2 Power Analysis

Power calculations for EXERT were based on two-sample t-tests. Sample sizes were estimated using 12-month ADAS-Cog change scores from the ADCS MCI trial, targeting power =80%, 2-sided alpha =5%, and SD =4.1. Calculations ranged over effect sizes in ADAS-Cog from 1.0-2.0, and dropout rates from between 10-25%. The trial is powered to conservatively accommodate 20% attrition. Based on these considerations, and an effect size =1.5, 291 total subjects are required. The trial will enroll 300 participants.

3.3 Randomization Plan

Each patient will be randomly allocated in a 1:1 ratio into one of the 2 groups: Aerobic Exercise (AX) or Stretching/Balance/Range Of Motion(SBR). A randomization schedule will be generated and incorporated into the Electronic Data Capture (EDC) system and the treatment group will be assigned as the site activates the patient. A centralized eligibility evaluation procedure will be applied for each patient. A stratified permuted block randomization procedure will be used. Site, ApoE4 carrier status, and gender will be stratification factors.

3.4 Interim Analysis Plan

There is no proposed interim analysis for the study at this time point.

3.5 Extension Period

After 12 months of supervised exercise, all participants will transition to independent exercise (without supervision) and will be instructed to continue their assigned physical activity regimen for an additional 6 months to test the efficacy of a translational model.

CONFIDENTIALITY STATEMENT: The information contained in this document is confidential and may not be reproduced or otherwise distributed without the approval of the Biostatistics Research Center Directors.

4 Methods

4.1 Data Set

This report is based on all data downloaded on 03/16/2022 from the ADCS EXERT portal (https://exe.adcs.ucsd.edu/docs/studylock).

4.2 Arm Code Assignment

This report is presented using unblinded arm codes: SBR and AX.

CONFIDENTIALITY STATEMENT: The information contained in this document is confidential and may not be reproduced or otherwise distributed without the approval of the Biostatistics Research Center Directors.

5 Enrollment and Participant Status

This section summarizes the participant status, enrollment by study month, and enrollment by randomization strata. Enrollment is defined as all randomized participants. Projected vs actual enrollment numbers are also presented graphically.

5.1 Participant Status

	N	Percent
**Screened**	**986**	**100**
Never Randomized (screen fails)	690	70
Randomized	296	30
Pending	0	0

- Screened: Number of subjects who have had the screening visit based on the Registry form.
- Never Randomized: Number of subjects who were indicated as 'never randomized' based on the Study Summary form. Screen fails encompasses all participants that were never randomized.
- Randomized: Number of subjects who have been randomized.
- Pending: Number of screened - number of never randomized - number of randomized.

CONFIDENTIALITY STATEMENT: The information contained in this document is confidential and may not be reproduced or otherwise distributed without the approval of the Biostatistics Research Center Directors.

5.2 Enrollment by Month

Month	Actual Monthly	Actual Cumulative
2016-09	2	2
2016-10	0	2
2016-11	1	3
2016-12	0	3
2017-01	1	4
2017-02	3	7
2017-03	0	7
2017-04	2	9
2017-05	3	12
2017-06	1	13
2017-07	1	14
2017-08	10	24
2017-09	2	26
2017-10	3	29
2017-11	5	34
2017-12	4	38
2018-01	6	44
2018-02	2	46
2018-03	7	53
2018-04	10	63
2018-05	11	74
2018-06	13	87
2018-07	6	93
2018-08	7	100
2018-09	5	105
2018-10	9	114
2018-11	10	124
2018-12	9	133
2019-01	14	147
2019-02	6	153
2019-03	9	162
2019-04	20	182
2019-05	15	197
2019-06	9	206
2019-07	10	216
2019-08	14	230
2019-09	9	239
2019-10	10	249
2019-11	9	258
2019-12	7	265

CONFIDENTIALITY STATEMENT: The information contained in this document is confidential and may not be reproduced or otherwise distributed without the approval of the Biostatistics Research Center Directors.

Month	Actual Monthly	Actual Cumulative
2020-01	14	279
2020-02	17	296
2020-03	0	296
2020-04	0	296
2020-05	0	296
2020-06	0	296
2020-07	0	296
2020-08	0	296
2020-09	0	296
2020-10	0	296
2020-11	0	296
2020-12	0	296
2021-01	0	296
2021-02	0	296
2021-03	0	296
2021-04	0	296
2021-05	0	296
2021-06	0	296
2021-07	0	296
2021-08	0	296
2021-09	0	296
2021-10	0	296
2021-11	0	296
2021-12	0	296
2022-01	0	296
2022-02	0	296
2022-03	0	296

CONFIDENTIALITY STATEMENT: The information contained in this document is confidential and may not be reproduced or otherwise distributed without the approval of the Biostatistics Research Center Directors.

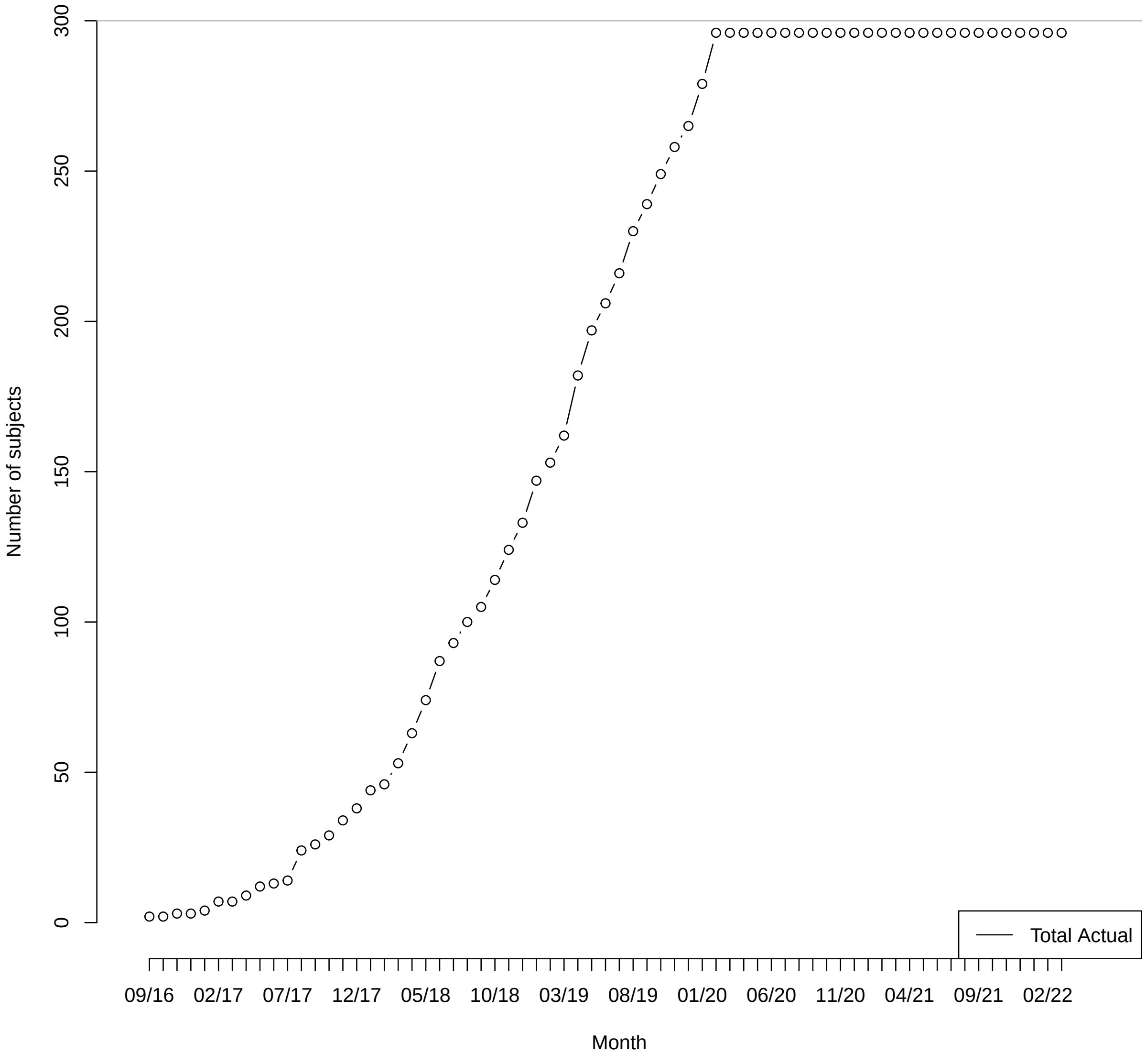

The projected enrollment number is 300. The screening was started on July 13, 2016. The enrollment/randomization has started in September 2016.

5.3 Enrollment by Randomization Strata

5.3.1 Enrollment by Site

296 subjects have been enrolled in 14 sites.

	AX	SBR	Overall
013 MSSM	10	9	19
020 NYU	2	3	5
021 DUKE	11	11	22
023 UKY	8	6	14
029 UCI	14	15	29
032 EMORY	4	5	9
033 KANSAS	5	5	10
041 YALE	19	20	39
127 UWI	24	21	45
137 WAKE	23	24	47
145 STANFORD	15	16	31
153 CCLRC	3	3	6
183 UNTHSC	5	5	10
302 GLCT	5	5	10
Overall	148	148	296

5.3.2 Enrollment by Baseline ApoE4 carrier status

	AX	SBR	Total	Pvalue
non-carriers	111 (75%)	111 (75%)	222 (75%)	Not Tested
carriers	37 (25%)	37 (25%)	74 (25%)	
Total	148 (100%)	148 (100%)	296 (100%)	

6 Visit Schedule Summary

This section includes the visit summary for the randomized subjects, by blinded arm code and overall. Visits not done are not included in the counts.

Note: There are 0 subjects who were randomized, but haven't completed all the baseline forms entry.

CONFIDENTIALITY STATEMENT: The information contained in this document is confidential and may not be reproduced or otherwise distributed without the approval of the Biostatistics Research Center Directors.

	AX	SBR	Overall
sc	148	148	296
sc2	148	148	296
bl	148	148	296
m06	118	122	240
m12	114	115	229
m18	92	86	178

7 Screen Fail

This section summarizes the screen fails by inclusions/exclusion criteria.
Note: Percentage is calculated based on the total number of criterias selected (N=644). One subject might have more than one criteria selected. There are 131 subjects who screen failed where reason for fail has not yet been entered into the database. Please refer to the Appendix for inclusion/exclusion criteria details.

7.1 Screen Fail by Inclusion Criteria

	N	Percent
Age 65-89	8	1
amnestic MCI	21	3
MMSE $\geq$24/22	14	2
CDR =0.5	68	11
Impaired delayed verbal recall	60	9
Speaks English fluently	2	0
Visual and auditory acuity adequate for cognitive testing	0	0
exclude mental retardation	0	0
Has an informant	34	5
Sedentary or underactive	125	19
Willing to be randomized	26	4
Willing and able to reliably travel to the identified YMCA	39	6
Ability to safely participate in either intervention and complete the 400 m Walk Test	11	2
reside in the area for at least 18 months	2	0
planned travel total time no more than 2 months	2	0
good general health	5	1
Hachinski $\leq$4	2	0
Stable use of certain drugs	4	1
Stable use of antidepressants	1	0
willing to complete 4-week washout of psychoactive medications	0	0
complete all baseline assessments	5	1

7.2 Screen Fail by Exclusion Criteria

	N	Percent
Any significant neurologic disease, other than MCI	21	3
Sensory or musculoskeletal impairment	5	1
Contraindications for MRI studies	19	3
Brain MRI	4	1
major depression or bipolar disorder	13	2
History of schizophrenia	1	0
alcohol or substance abuse	2	0
consumes more than 3 alcoholic drinks per day	3	0
unstable medical condition	34	5
History in the last 6 months of myocardial infarction, coronary artery angioplasty, bypass grafting, or STENT placement	2	0
History in the last 3 months of transient ischemic attack or small vessel stroke	3	0
joint replacement surgery	3	0
malignant disease	15	2
Hemoglobin A1c >7.0	12	2
abnormal lab	4	1
use of insulin to treat type 2 diabetes	12	2
current use of psychoactive medications	15	2
Current use of memantine	4	1
anxiolytics or sedative hypnotics	2	0
active immunization against amyloid	0	0
Previous treatment with agents with anti-amyloid properties	0	0
anticoagulants	1	0
blood clotting or bleeding disorder	0	0
physical distortions	0	0
PI decision	40	6

CONFIDENTIALITY STATEMENT: The information contained in this document is confidential and may not be reproduced or otherwise distributed without the approval of the Biostatistics Research Center Directors.

8 Early Discontinuation

This section summarizes the early discontinuations by reasons for the randomized subjects. Early discontinuation is defined as discontinuing supervised exercise intervention.
Note: Since one of the death (EXE0290916) from June 1st report was changed to "adverse event", total early discontinuations due to death changed from 3 to 2.

	**AX** (N=148)	**SBR** (N=148)	**Overall** (N=296)
Death	1 (1%)	1 (1%)	2 (1%)
Safety risk	2 (1%)	1 (1%)	3 (1%)
Perceived lack of efficacy	0 (0%)	0 (0%)	0 (0%)
Non-compliance	1 (1%)	1 (1%)	2 (1%)
Adverse event	4 (3%)	3 (2%)	7 (2%)
Investigator recommendation	0 (0%)	0 (0%)	0 (0%)
Other non-site clinician recommendation	1 (1%)	0 (0%)	1 (0%)
Started prohibited medication	0 (0%)	0 (0%)	0 (0%)
Participant unwilling or unable to participate	20 (14%)	14 (9%)	34 (11%)
Study partner unwilling or unable to participate	0 (0%)	0 (0%)	0 (0%)
Study terminated	1 (1%)	2 (1%)	3 (1%)
Starting new trial	0 (0%)	0 (0%)	0 (0%)
Lost to follow-up	3 (2%)	4 (3%)	7 (2%)
Coordinating Center request	0 (0%)	0 (0%)	0 (0%)
Other	0 (0%)	4 (3%)	4 (1%)
TOTAL	33 (22%)	30 (20%)	63 (21%)

CONFIDENTIALITY STATEMENT: The information contained in this document is confidential and may not be reproduced or otherwise distributed without the approval of the Biostatistics Research Center Directors.

9 Demographics

This section includes the key demographics for the randomized subjects.

Categorical measures:
- Gender
- Race
- Ethnicity
- Marital Status
- Retirement
- Home

Continuous measures:
- Age (years)
- Education (years)

Continuous variables are summarized with N, mean, standard deviation, minmum, 25thQ, median, 75thQ, and maximum, by blinded armcode and overall. Kruskal-Wallis test will be used for the comparison when there are at least 5 subjects in each group. Categorical variables are summarized using frequency tables and percentages. Fisher's exact test will be used for the comparison when there are at least 5 subjects in each group.

CONFIDENTIALITY STATEMENT: The information contained in this document is confidential and may not be reproduced or otherwise distributed without the approval of the Biostatistics Research Center Directors.

	AX	SBR	Total	Pvalue
**Gender**				
Male	63 (42.57%)	64 (43.24%)	127 (42.91%)	>0.999
Female	85 (57.43%)	84 (56.76%)	169 (57.09%)	
Total	148 (100%)	148 (100%)	296 (100%)	
**Race**				
American Indian or Alaskan Native	2 (1.36%)	1 (0.68%)	3 (1.02%)	0.952
Asian	3 (2.04%)	4 (2.7%)	7 (2.37%)	
Native Hawaiian or Other Pacific Islander	0 (0%)	0 (0%)	0 (0%)	
Black or African American	15 (10.2%)	14 (9.46%)	29 (9.83%)	
White	127 (86.39%)	129 (87.16%)	256 (86.78%)	
Unknown or Not Reported	0 (0%)	0 (0%)	0 (0%)	
Multiple Race	0 (0%)	0 (0%)	0 (0%)	
Total	147 (99.99%)	148 (100%)	295 (100%)	
**Ethnicity**				
Hispanic or Latino	3 (2.03%)	0 (0%)	3 (1.01%)	0.408
Not Hispanic or Latino	142 (95.95%)	145 (97.97%)	287 (96.96%)	
Unknown or Not Reported	3 (2.03%)	3 (2.03%)	6 (2.03%)	
Total	148 (100.01%)	148 (100%)	296 (100%)	
**Marital Status**				
Married	92 (62.16%)	93 (62.84%)	185 (62.5%)	0.986
Widowed	16 (10.81%)	17 (11.49%)	33 (11.15%)	
Divorced	33 (22.3%)	30 (20.27%)	63 (21.28%)	
Never married	6 (4.05%)	7 (4.73%)	13 (4.39%)	
Unknown/Other	1 (0.68%)	1 (0.68%)	2 (0.68%)	
Total	148 (100%)	148 (100.01%)	296 (100%)	
**Retired**				
No	30 (20.27%)	33 (22.3%)	63 (21.28%)	0.888
Yes	117 (79.05%)	114 (77.03%)	231 (78.04%)	
Not Applicable	1 (0.68%)	1 (0.68%)	2 (0.68%)	
Total	148 (100%)	148 (100.01%)	296 (100%)	
**Home**				
Independent Living	142 (95.95%)	144 (97.3%)	286 (96.62%)	0.584
Lives with family	5 (3.38%)	2 (1.35%)	7 (2.36%)	
Senior Residence	1 (0.68%)	2 (1.35%)	3 (1.01%)	
Assisted Living	0 (0%)	0 (0%)	0 (0%)	
Skilled Nursing Facility	0 (0%)	0 (0%)	0 (0%)	
Other	0 (0%)	0 (0%)	0 (0%)	
Total	148 (100.01%)	148 (100%)	296 (99.99%)	

CONFIDENTIALITY STATEMENT: The information contained in this document is confidential and may not be reproduced or otherwise distributed without the approval of the Biostatistics Research Center Directors.

	N	Mean	SD	Min	Q1	Median	Q3	Max	Pvalue
**Age**									
AX	148	74.26	5.71	65	70	73	77.82	88	0.764
SBR	148	74.65	6.23	65.04	69.73	73.74	79.45	89.99	
Overall	296	74.46	5.96	65	69.85	73.25	78.73	89.99	
**Education(years)**									
AX	148	16.2	2.36	12	14	16	18	22	0.867
SBR	148	16.26	2.36	10	14.75	16	18	20	
Overall	296	16.23	2.36	10	14	16	18	22	

CONFIDENTIALITY STATEMENT: The information contained in this document is confidential and may not be reproduced or otherwise distributed without the approval of the Biostatistics Research Center Directors.

10 Vital Signs

This section summarizes the following vital sign measures.

- Weight (kg) at sc2, bl, m06,m12,m18
- BMI at sc2, bl, m06,m12,m18
- Systolic Blood Pressure (mm Hg) at sc2, bl, m06,m12,m18
- Diastolic Blood Pressure (mm Hg) at sc2, bl, m06,m12,m18
- Pulse (bpm) at sc2, bl, m06,m12,m18

Vital signs are summarized with N, mean, standard deviation, minimum, 25thQ, median, 75thQ, and maximum at each scheduled visit, by blinded armcode and overall. Kruskal-Wallis test will be used for the comparison when there are at least 5 subjects in each group at each visit.

CONFIDENTIALITY STATEMENT: The information contained in this document is confidential and may not be reproduced or otherwise distributed without the approval of the Biostatistics Research Center Directors.

10.1 Weight (kg)

	N	Mean	SD	Min	Q1	Median	Q3	Max	Pvalue
**sc2**									
AX	148	79.9	17.71	44.8	68	77.3	89.2	135	0.863
SBR	148	80.81	19	46.1	67.35	78.4	90.9	150.5	
Overall	296	80.36	18.34	44.8	67.57	77.55	90.1	150.5	
**bl**									
AX	147	79.53	17.59	44.6	68.2	76.7	89.15	135.9	0.618
SBR	147	81.03	18.87	47.1	67.7	77.5	91.15	150	
Overall	294	80.28	18.23	44.6	67.85	76.85	90.7	150	
**m06**									
AX	117	78.69	17.1	43.7	68.6	75.3	88.5	127.3	0.917
SBR	122	79.82	18.95	44.5	67.15	77.45	89.3	149.5	
Overall	239	79.27	18.04	43.7	67.3	76.8	89.1	149.5	
**m12**									
AX	111	78.98	17.09	43.2	69.5	78.8	89.7	128.2	0.934
SBR	113	80.19	19.08	45.9	65.9	78.2	89.1	150	
Overall	224	79.6	18.09	43.2	67.07	78.2	89.28	150	
**m18**									
AX	89	80.11	16.08	44.5	70.2	80.1	89.6	126.1	0.766
SBR	85	80.57	18.34	49.2	68.5	78.9	89.6	145.5	
Overall	174	80.34	17.18	44.5	68.95	79.35	89.6	145.5	

CONFIDENTIALITY STATEMENT: The information contained in this document is confidential and may not be reproduced or otherwise distributed without the approval of the Biostatistics Research Center Directors.

10.2 BMI

	N	Mean	SD	Min	Q1	Median	Q3	Max	Pvalue
**sc2**									
AX	148	28.65	5.63	18.76	24.97	27.6	31.86	48.06	0.832
SBR	147	29.05	5.95	18.56	24.5	28.15	31.79	51.58	
Overall	295	28.85	5.79	18.56	24.84	27.99	31.83	51.58	
**bl**									
AX	147	28.56	5.64	18.76	25.05	27.26	31.66	48.38	0.611
SBR	146	29.13	6.01	18.99	24.55	28.48	31.97	52.66	
Overall	293	28.85	5.83	18.76	24.98	28.03	31.74	52.66	
**m06**									
AX	117	28.04	5.38	17.92	24.43	27.57	30.94	45.38	0.624
SBR	121	28.65	5.79	18.36	24.09	28.06	31.44	47.29	
Overall	238	28.35	5.59	17.92	24.21	27.88	31.14	47.29	
**m12**									
AX	111	28.04	5.22	18.05	24.46	27.86	31.33	45.64	0.696
SBR	112	28.72	5.85	18.93	24.28	28.24	31.83	47.45	
Overall	223	28.38	5.54	18.05	24.27	28.03	31.56	47.45	
**m18**									
AX	89	28.32	4.94	19.38	25.04	27.73	31.44	43.28	0.774
SBR	85	28.69	5.38	20.09	23.95	28.53	32.05	44.26	
Overall	174	28.5	5.15	19.38	24.69	28.35	31.51	44.26	

CONFIDENTIALITY STATEMENT: The information contained in this document is confidential and may not be reproduced or otherwise distributed without the approval of the Biostatistics Research Center Directors.

10.3 Systolic Blood Pressure (mm Hg)

	N	Mean	SD	Min	Q1	Median	Q3	Max	Pvalue
**sc2**									
AX	148	136.04	15.39	100	124	134	147	194	0.143
SBR	148	139.8	20.05	91	127	138	150	214	
Overall	296	137.92	17.94	91	125	136	148	214	
**bl**									
AX	148	133.16	16.09	94	121	132	143.25	178	0.254
SBR	148	136.01	18.58	95	122	133	148	193	
Overall	296	134.59	17.41	94	122	132	145.25	193	
**m06**									
AX	117	133.72	16.89	92	122	132	144	181	0.256
SBR	123	137.2	20.22	96	123.5	134	149	200	
Overall	240	135.5	18.71	92	123	133	146	200	
**m12**									
AX	112	133.93	17.09	101	122	131.5	142	191	0.409
SBR	114	135.67	18.9	91	122	135	150.5	185	
Overall	226	134.81	18.01	91	122	133	146.75	191	
**m18**									
AX	90	131.82	16.36	80	120.25	130.5	144	171	0.364
SBR	85	135.52	19	98	121	133	149	183	
Overall	175	133.62	17.74	80	121	132	145	183	

CONFIDENTIALITY STATEMENT: The information contained in this document is confidential and may not be reproduced or otherwise distributed without the approval of the Biostatistics Research Center Directors.

10.4 Diastolic Blood Pressure (mm Hg)

	N	Mean	SD	Min	Q1	Median	Q3	Max	Pvalue
**sc2**									
AX	148	76.24	9.49	54	69.75	76	83	106	0.295
SBR	148	77.64	9.99	51	70	77.5	83	108	
Overall	296	76.94	9.75	51	70	77	83	108	
**bl**									
AX	148	75.05	9.17	49	70	76.5	81.25	96	0.404
SBR	148	74.82	9.96	49	69	74.5	80	104	
Overall	296	74.94	9.56	49	70	76	81	104	
**m06**									
AX	117	76.45	10.16	53	71	77	82	103	0.659
SBR	123	77.48	10.91	50	71	78	83	115	
Overall	240	76.98	10.54	50	71	77	83	115	
**m12**									
AX	112	75.57	9.88	45	69	75	81.25	102	0.46
SBR	114	76.32	10.19	53	70	76	82.75	105	
Overall	226	75.95	10.02	45	69	75	82	105	
**m18**									
AX	90	74.7	8.79	53	68.25	75	80.75	97	0.025
SBR	85	78.13	9.58	58	71	78	84	104	
Overall	175	76.37	9.32	53	69	77	83	104	

CONFIDENTIALITY STATEMENT: The information contained in this document is confidential and may not be reproduced or otherwise distributed without the approval of the Biostatistics Research Center Directors.

10.5 Pulse (bpm)

	N	Mean	SD	Min	Q1	Median	Q3	Max	Pvalue
**sc2**									
AX	148	67.02	10.63	46	60	66.5	72.25	96	0.86
SBR	148	66.57	10.14	40	60	66	73	93	
Overall	296	66.8	10.37	40	60	66	73	96	
**bl**									
AX	148	67.72	9.32	44	61	67	74	90	0.224
SBR	148	66.82	10.85	44	60	65	74	114	
Overall	296	67.27	10.11	44	60.75	66	74	114	
**m06**									
AX	118	66.64	10.89	44	60	65	72.75	120	0.421
SBR	123	67.47	10.94	38	60	67	74	100	
Overall	241	67.07	10.9	38	60	66	74	120	
**m12**									
AX	112	66.45	8.81	51	59	66	71	95	0.45
SBR	114	67.33	9.91	45	61	66.5	73.75	95	
Overall	226	66.89	9.37	45	60	66	72	95	
**m18**									
AX	90	68.39	11.43	45	61	66.5	74.75	99	0.986
SBR	85	67.99	10.83	42	60	68	74	91	
Overall	175	68.19	11.11	42	60.5	67	74	99	

CONFIDENTIALITY STATEMENT: The information contained in this document is confidential and may not be reproduced or otherwise distributed without the approval of the Biostatistics Research Center Directors.

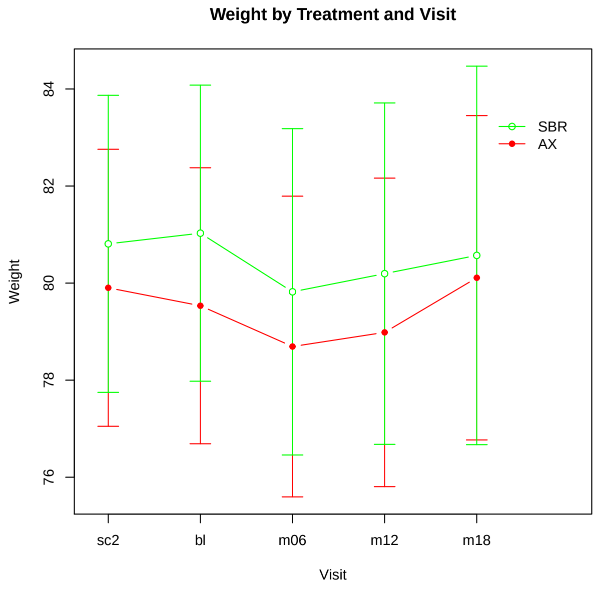

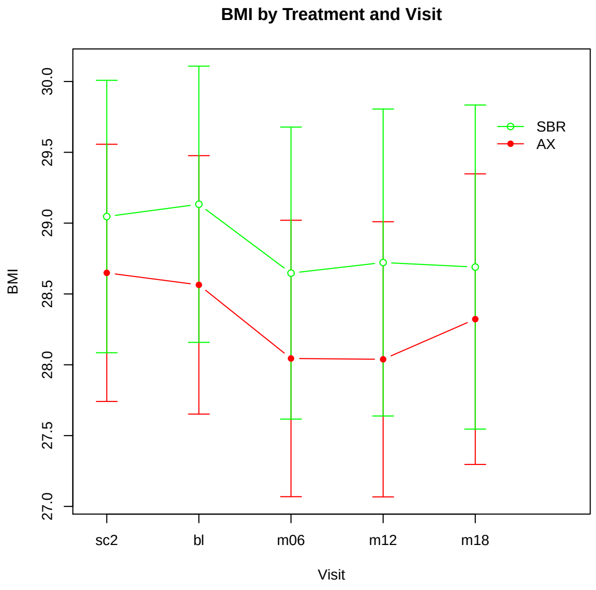

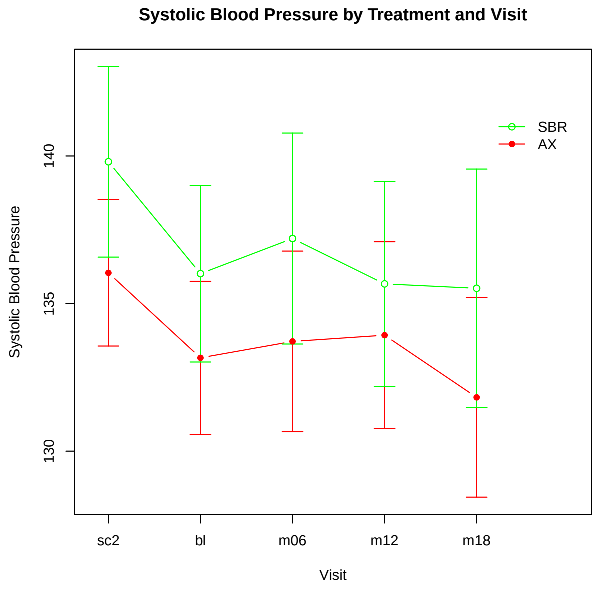

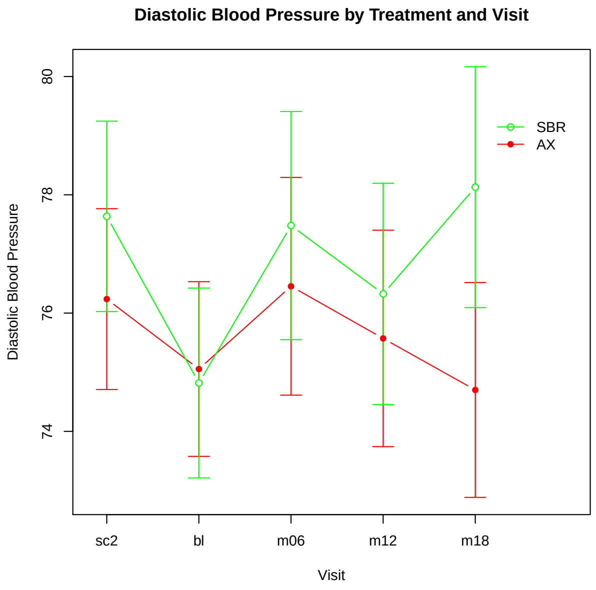

CONFIDENTIALITY STATEMENT: The information contained in this document is confidential and may not be reproduced or otherwise distributed without the approval of the Biostatistics Research Center Directors.

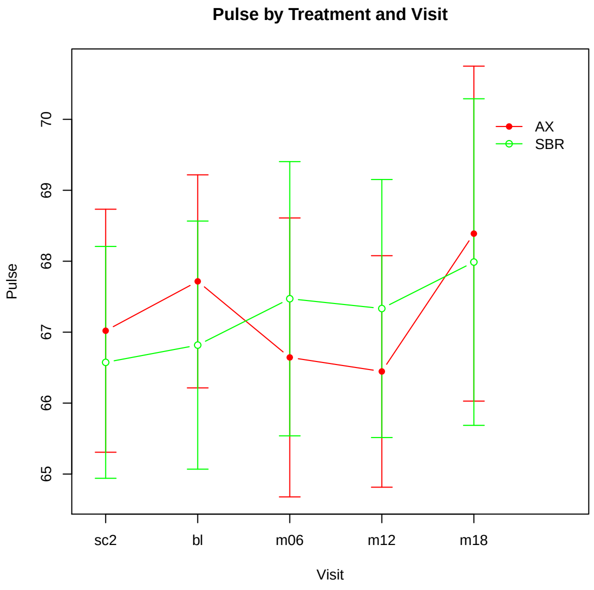
Pulse by Treatment and Visit
Pulse
65
66
67
68
69
70
sc2
bl
m06
m12
m18
Visit
AX
SBR

11 Safety in the Main Study Period

11.1 Adverse Events with Onset Before Randomization

	N of events	N of subjects with event
AEs for subjects never randomized (n=690)	12	7
AEs for subjects pending randomization (n=0)	0	0
AEs for randomized subjects with onset before randomization (n=296)	39	32

Detailed AE listings are provided in Addendum Report 1.

11.2 Adverse Events with Onset On or After Randomization

This section summarizes AEs for randomized subjects with onset date on or after the baseline visit date, and before month 12 visit date, by blinded armcode and overall. Detailed AE listings (including uncoded AEs) are provided in Addendum Report 1.

11.2.1 AE Overall Summary

	**AX**	**SBR**	**Overall**	
	(N=148)	(N=148)	(N=296)	P.value
Number of AE events	344	311	655	
Number of subjects who experienced at least one AE	114	109	223	
Percent of subjects who experienced at least one AE	77.03%	73.65%	75.34%	0.7958

P.value is from Fisher's exact test comparing the proportions of subjects with AE between the groups.

11.2.2 AE by Severity

	AX	SBR	Total	Pvalue
Mild	209 (60.76%)	193 (62.06%)	402 (61.37%)	Not Tested
Moderate	109 (31.69%)	94 (30.23%)	203 (30.99%)	
Severe	26 (7.56%)	24 (7.72%)	50 (7.63%)	
Total	344 (100.01%)	311 (100.01%)	655 (99.99%)	

11.2.3 AE by Relationship to Investigational Product

	AX	SBR	Total	Pvalue
Definitely	5 (1.45%)	4 (1.29%)	9 (1.37%)	Not Tested
Probably	16 (4.65%)	7 (2.25%)	23 (3.51%)	
Possibly	48 (13.95%)	39 (12.54%)	87 (13.28%)	
Unlikely	43 (12.5%)	57 (18.33%)	100 (15.27%)	
Unrelated	224 (65.12%)	197 (63.34%)	421 (64.27%)	
Prior to initial exposure to intervention	8 (2.33%)	7 (2.25%)	15 (2.29%)	
Total	344 (100%)	311 (100%)	655 (99.99%)	

CONFIDENTIALITY STATEMENT: The information contained in this document is confidential and may not be reproduced or otherwise distributed without the approval of the Biostatistics Research Center Directors.

11.2.4 Approved MedDRA Coded AEs

Due to the real-time data entry, there may be AEs that have not been MedDRA coded at the time of data freeze. Only coded AEs that have been approved are included in this part.

Number of AEs summarized by MedDRA SOC

	AX	SBR	Total
Cardiac disorders	11	9	20
Ear and labyrinth disorders	4	1	5
Endocrine disorders	1	0	1
Eye disorders	4	4	8
Gastrointestinal disorders	9	19	28
General disorders and administration site conditions	13	15	28
Immune system disorders	3	3	6
Infections and infestations	60	51	111
Injury, poisoning and procedural complications	76	59	135
Investigations	6	8	14
Metabolism and nutrition disorders	2	0	2
Musculoskeletal and connective tissue disorders	78	70	148
Neoplasms benign, malignant and unspecified (incl cysts and polyps)	6	6	12
Nervous system disorders	18	29	47
Psychiatric disorders	4	6	10
Renal and urinary disorders	4	6	10
Reproductive system and breast disorders	1	0	1
Respiratory, thoracic and mediastinal disorders	8	3	11
Skin and subcutaneous tissue disorders	8	2	10
Surgical and medical procedures	19	17	36
Vascular disorders	9	3	12
Total	344	311	655

CONFIDENTIALITY STATEMENT: The information contained in this document is confidential and may not be reproduced or otherwise distributed without the approval of the Biostatistics Research Center Directors.

Number of participants with at least one AE summarized by MedDRA SOC

	AX (N=148)	SBR (N=148)	Overall (N=296)
Cardiac disorders	8 (5%)	7 (5%)	15 (5%)
Ear and labyrinth disorders	4 (3%)	1 (1%)	5 (2%)
Endocrine disorders	1 (1%)	0 (0%)	1 (0%)
Eye disorders	4 (3%)	4 (3%)	8 (3%)
Gastrointestinal disorders	7 (5%)	18 (12%)	25 (8%)
General disorders and administration site conditions	13 (9%)	14 (9%)	27 (9%)
Immune system disorders	3 (2%)	3 (2%)	6 (2%)
Infections and infestations	40 (27%)	40 (27%)	80 (27%)
Injury, poisoning and procedural complications	51 (34%)	42 (28%)	93 (31%)
Investigations	6 (4%)	7 (5%)	13 (4%)
Metabolism and nutrition disorders	2 (1%)	0 (0%)	2 (1%)
Musculoskeletal and connective tissue disorders	51 (34%)	50 (34%)	101 (34%)
Neoplasms benign, malignant and unspecified (incl cysts and polyps)	6 (4%)	6 (4%)	12 (4%)
Nervous system disorders	18 (12%)	22 (15%)	40 (14%)
Psychiatric disorders	4 (3%)	4 (3%)	8 (3%)
Renal and urinary disorders	3 (2%)	5 (3%)	8 (3%)
Reproductive system and breast disorders	1 (1%)	0 (0%)	1 (0%)
Respiratory, thoracic and mediastinal disorders	8 (5%)	3 (2%)	11 (4%)
Skin and subcutaneous tissue disorders	6 (4%)	2 (1%)	8 (3%)
Surgical and medical procedures	14 (9%)	14 (9%)	28 (9%)
Vascular disorders	8 (5%)	3 (2%)	11 (4%)

CONFIDENTIALITY STATEMENT: The information contained in this document is confidential and may not be reproduced or otherwise distributed without the approval of the Biostatistics Research Center Directors.

Number of AEs summarized by MedDRA SOC and PT

	AX		SBR		Overall	
	Event	Subj	Event	Subj	Event	Subj
CARDIAC DISORDERS	11	8	9	7	20	15
Arrhythmia	2	1	2	2	4	3
Atrial fibrillation	1	1	3	3	4	4
Atrial flutter	2	2	0	0	2	2
Cardiac arrest	1	1	1	1	2	2
Cardiac failure	0	0	1	1	1	1
Cardiac failure congestive	1	1	0	0	1	1
Mitral valve incompetence	0	0	1	1	1	1
Myocardial infarction	1	1	0	0	1	1
Presyncope	1	1	0	0	1	1
Tachycardia	2	1	0	0	2	1
Ventricular extrasystoles	0	0	1	1	1	1
EAR AND LABYRINTH DISORDERS	4	4	1	1	5	5
Hypoacusis	1	1	0	0	1	1
Tinnitus	1	1	0	0	1	1
Vertigo	2	2	1	1	3	3
ENDOCRINE DISORDERS	1	1	0	0	1	1
Hyperparathyroidism	1	1	0	0	1	1
EYE DISORDERS	4	4	4	4	8	8
Blepharospasm	1	1	0	0	1	1
Cataract	0	0	2	2	2	2
Glaucoma	1	1	1	1	2	2
Lenticular opacities	0	0	1	1	1	1
Subretinal fluid	1	1	0	0	1	1
Vitreous floaters	1	1	0	0	1	1
GASTROINTESTINAL DISORDERS	9	7	19	18	28	25
Abdominal discomfort	2	2	3	3	5	5
Abdominal pain	0	0	2	2	2	2
Abdominal pain upper	0	0	2	2	2	2
Colitis microscopic	1	1	0	0	1	1
Colitis ulcerative	1	1	0	0	1	1
Constipation	1	1	0	0	1	1
Dental caries	0	0	1	1	1	1
Diarrhoea	1	1	4	4	5	5
Gastrointestinal disorder	1	1	0	0	1	1
Gastrooesophageal reflux disease	0	0	1	1	1	1
Haemorrhoids	0	0	1	1	1	1
Nausea	2	2	0	0	2	2
Pancreatitis	0	0	1	1	1	1
Toothache	0	0	2	2	2	2

	AX		SBR		Overall	
	Event	Subj	Event	Subj	Event	Subj
Volvulus	0	0	1	1	1	1
Vomiting	0	0	1	1	1	1
GENERAL DISORDERS AND ADMINISTRATION SITE CONDITIONS	13	13	15	14	28	27
Asthenia	0	0	1	1	1	1
Calcinosis	0	0	1	1	1	1
Chest discomfort	0	0	2	2	2	2
Chest pain	2	2	5	5	7	7
Chills	1	1	0	0	1	1
Device failure	0	0	1	1	1	1
Hernia	0	0	1	1	1	1
Inflammation	1	1	1	1	2	2
Influenza like illness	1	1	0	0	1	1
Lethargy	1	1	0	0	1	1
Non-cardiac chest pain	1	1	0	0	1	1
Oedema peripheral	5	5	1	1	6	6
Pain	1	1	1	1	2	2
Peripheral swelling	0	0	1	1	1	1
IMMUNE SYSTEM DISORDERS	3	3	3	3	6	6
Hypersensitivity	2	2	1	1	3	3
Seasonal allergy	1	1	2	2	3	3
INFECTIONS AND INFESTATIONS	60	40	51	40	111	80
Abdominal abscess	0	0	1	1	1	1
Abscess	0	0	1	1	1	1
Appendicitis	1	1	0	0	1	1
Bacterial infection	1	1	0	0	1	1
Bronchitis	1	1	3	3	4	4
Cellulitis	1	1	2	2	3	3
Clostridium difficile colitis	1	1	0	0	1	1
Clostridium difficile infection	1	1	0	0	1	1
Corona virus infection	4	3	1	1	5	4
Cystitis	0	0	3	2	3	2
Ear infection	1	1	1	1	2	2
Gastroenteritis	0	0	1	1	1	1
Gastroenteritis viral	2	2	0	0	2	2
Gastrointestinal viral infection	1	1	0	0	1	1
Gingivitis	0	0	1	1	1	1
Herpes zoster	1	1	1	1	2	2
Influenza	3	3	4	4	7	7
Kidney infection	1	1	0	0	1	1
Localised infection	1	1	0	0	1	1

	AX		SBR		Overall	
	Event	Subj	Event	Subj	Event	Subj
Lower respiratory tract infection	1	1	0	0	1	1
Nasopharyngitis	12	11	8	8	20	19
Pneumonia	5	4	3	3	8	7
Respiratory tract infection	2	1	0	0	2	1
Rhinitis	1	1	0	0	1	1
Root canal infection	1	1	0	0	1	1
Sinusitis	1	1	4	4	5	5
Tooth abscess	0	0	1	1	1	1
Tooth infection	0	0	1	1	1	1
Upper respiratory tract infection	9	6	9	7	18	13
Upper respiratory tract infection bacterial	1	1	0	0	1	1
Urinary tract infection	5	5	6	5	11	10
Viral infection	1	1	0	0	1	1
Viral rhinitis	1	1	0	0	1	1
INJURY, POISONING AND PROCEDURAL COMPLICATIONS	76	51	59	42	135	93
Ankle fracture	0	0	1	1	1	1
Arthropod bite	1	1	0	0	1	1
Back injury	0	0	1	1	1	1
Chemical eye injury	1	1	0	0	1	1
Contusion	2	2	2	1	4	3
Dermatitis contact	1	1	0	0	1	1
Fall	39	31	29	23	68	54
Foot fracture	3	3	3	3	6	6
Head injury	1	1	0	0	1	1
Hip fracture	1	1	1	1	2	2
Humerus fracture	1	1	0	0	1	1
Injection site pain	1	1	0	0	1	1
Joint dislocation	0	0	1	1	1	1
Joint injury	1	1	1	1	2	2
Laceration	1	1	2	2	3	3
Ligament sprain	5	5	1	1	6	6
Limb injury	5	5	1	1	6	6
Meniscus injury	0	0	1	1	1	1
Muscle rupture	1	1	0	0	1	1
Muscle strain	5	5	5	5	10	10
Neuroleptic malignant syndrome	1	1	0	0	1	1
Overdose	0	0	1	1	1	1
Post lumbar puncture syndrome	1	1	0	0	1	1
Procedural dizziness	2	1	0	0	2	1
Rib fracture	0	0	1	1	1	1

	AX		SBR		Overall	
	Event	Subj	Event	Subj	Event	Subj
Road traffic accident	0	0	1	1	1	1
Skin abrasion	1	1	2	1	3	2
Stress fracture	0	0	1	1	1	1
Tendon injury	1	1	1	1	2	2
Upper limb fracture	1	1	0	0	1	1
Vaccination complication	0	0	2	2	2	2
Wrist fracture	0	0	1	1	1	1
INVESTIGATIONS	6	6	8	7	14	13
Antinuclear antibody positive	0	0	1	1	1	1
Arthroscopy	1	1	0	0	1	1
Biopsy skin	1	1	0	0	1	1
Catheterisation cardiac	0	0	1	1	1	1
Haemoglobin decreased	0	0	1	1	1	1
Heart rate increased	1	1	1	1	2	2
Heart rate irregular	1	1	1	1	2	2
Neurological examination abnormal	1	1	0	0	1	1
Platelet count decreased	0	0	1	1	1	1
Pupillary light reflex tests abnormal	1	1	0	0	1	1
Troponin increased	0	0	2	1	2	1
METABOLISM AND NUTRITION DISORDERS	2	2	0	0	2	2
Hyperlipidaemia	2	2	0	0	2	2
MUSCULOSKELETAL AND CONNECTIVE TISSUE DISORDERS	78	51	70	50	148	101
Arthralgia	23	21	20	19	43	40
Arthritis	1	1	1	1	2	2
Back pain	17	13	15	15	32	28
Dupuytren's contracture	1	1	0	0	1	1
Intervertebral disc degeneration	1	1	0	0	1	1
Intervertebral disc protrusion	2	2	0	0	2	2
Joint instability	0	0	1	1	1	1
Joint swelling	1	1	0	0	1	1
Limb discomfort	0	0	1	1	1	1
Medial tibial stress syndrome	2	2	0	0	2	2
Muscle spasms	1	1	5	4	6	5
Muscular weakness	0	0	2	2	2	2
Musculoskeletal pain	7	5	8	8	15	13
Musculoskeletal stiffness	1	1	0	0	1	1
Myalgia	0	0	3	3	3	3
Neck pain	1	1	2	2	3	3
Osteoarthritis	0	0	2	2	2	2
Osteoporosis	0	0	2	2	2	2

	AX		SBR		Overall	
	Event	Subj	Event	Subj	Event	Subj
Pain in extremity	11	11	6	6	17	17
Pain in jaw	1	1	0	0	1	1
Patellofemoral pain syndrome	1	1	0	0	1	1
Periarthritis	1	1	0	0	1	1
Plantar fasciitis	1	1	0	0	1	1
Rotator cuff syndrome	0	0	1	1	1	1
Scoliosis	1	1	0	0	1	1
Synovial cyst	3	3	0	0	3	3
Tendonitis	1	1	1	1	2	2
NEOPLASMS BENIGN, MALIGNANT AND UNSPECIFIED (INCL CYSTS AND POLYPS)	6	6	6	6	12	12
B-cell lymphoma	1	1	0	0	1	1
Basal cell carcinoma	0	0	1	1	1	1
Breast cancer	1	1	0	0	1	1
Cyst	1	1	1	1	2	2
Lung carcinoma cell type unspecified stage IV	0	0	1	1	1	1
Lymphoma	0	0	1	1	1	1
Malignant melanoma	1	1	1	1	2	2
Meningioma	1	1	0	0	1	1
Squamous cell carcinoma	1	1	1	1	2	2
NERVOUS SYSTEM DISORDERS	18	18	29	22	47	40
Carpal tunnel syndrome	1	1	0	0	1	1
Cerebrovascular accident	1	1	0	0	1	1
Cognitive disorder	1	1	1	1	2	2
Dizziness	3	3	6	6	9	9
Dysarthria	0	0	1	1	1	1
Haemorrhagic stroke	0	0	1	1	1	1
Headache	2	2	2	2	4	4
Hypoaesthesia	0	0	1	1	1	1
Hyporeflexia	0	0	1	1	1	1
Nerve compression	0	0	1	1	1	1
Nervous system disorder	0	0	2	1	2	1
Neuralgia	1	1	0	0	1	1
Paraesthesia	1	1	1	1	2	2
Postural tremor	1	1	0	0	1	1
Presyncope	0	0	2	2	2	2
Sciatica	1	1	5	5	6	6
Seizure	0	0	1	1	1	1
Syncope	1	1	1	1	2	2
Transient ischaemic attack	1	1	2	2	3	3
Tremor	3	3	1	1	4	4

	AX		SBR		Overall	
	Event	Subj	Event	Subj	Event	Subj
Vertebral artery stenosis	1	1	0	0	1	1
PSYCHIATRIC DISORDERS	4	4	6	4	10	8
Anxiety	0	0	1	1	1	1
Delirium	1	1	0	0	1	1
Depression	0	0	1	1	1	1
Insomnia	1	1	1	1	2	2
Mental disorder	0	0	3	1	3	1
Somnolence	1	1	0	0	1	1
Suicidal ideation	1	1	0	0	1	1
RENAL AND URINARY DISORDERS	4	3	6	5	10	8
Acute kidney injury	0	0	1	1	1	1
Haematuria	1	1	3	2	4	3
Hypertonic bladder	1	1	0	0	1	1
Nephrolithiasis	2	2	1	1	3	3
Urinary incontinence	0	0	1	1	1	1
REPRODUCTIVE SYSTEM AND BREAST DISORDERS	1	1	0	0	1	1
Breast pain	1	1	0	0	1	1
RESPIRATORY, THORACIC AND MEDIASTINAL DISORDERS	8	8	3	3	11	11
Asthma	2	2	0	0	2	2
Chronic obstructive pulmonary disease	1	1	0	0	1	1
Cough	1	1	0	0	1	1
Dyspnoea exertional	1	1	0	0	1	1
Epistaxis	0	0	1	1	1	1
Pneumothorax	0	0	1	1	1	1
Pulmonary congestion	0	0	1	1	1	1
Pulmonary mass	1	1	0	0	1	1
Respiratory disorder	1	1	0	0	1	1
Sleep apnoea syndrome	1	1	0	0	1	1
SKIN AND SUBCUTANEOUS TISSUE DISORDERS	8	6	2	2	10	8
Blister	3	1	0	0	3	1
Blister rupture	1	1	0	0	1	1
Dermal cyst	0	0	1	1	1	1
Pigmentation disorder	1	1	0	0	1	1
Psoriasis	1	1	0	0	1	1
Rash	1	1	0	0	1	1
Skin lesion	1	1	0	0	1	1
Solar lentigo	0	0	1	1	1	1
SURGICAL AND MEDICAL PROCEDURES	19	14	17	14	36	28
Cancer surgery	1	1	1	1	2	2
Cardiac ablation	0	0	2	2	2	2

	AX		SBR		Overall	
	Event	Subj	Event	Subj	Event	Subj
Cardiac pacemaker insertion	0	0	1	1	1	1
Carotid endarterectomy	0	0	1	1	1	1
Carpal tunnel decompression	0	0	2	2	2	2
Cataract operation	3	3	5	3	8	6
Cholecystectomy	1	1	1	1	2	2
Dupuytren's contracture operation	1	1	0	0	1	1
Endodontic procedure	1	1	0	0	1	1
Hip arthroplasty	1	1	1	1	2	2
Intervertebral disc operation	1	1	0	0	1	1
Joint arthroplasty	0	0	1	1	1	1
Knee arthroplasty	1	1	0	0	1	1
Limb operation	1	1	0	0	1	1
Ptosis repair	1	1	0	0	1	1
Renal tumour excision	0	0	1	1	1	1
Rotator cuff repair	1	1	0	0	1	1
Stent placement	2	1	0	0	2	1
Tooth extraction	1	1	0	0	1	1
Transurethral prostatectomy	1	1	0	0	1	1
Umbilical hernia repair	2	2	0	0	2	2
Ureteral stent insertion	0	0	1	1	1	1
VASCULAR DISORDERS	9	8	3	3	12	11
Artery dissection	1	1	0	0	1	1
Hypertension	5	5	3	3	8	8
Orthostatic hypotension	1	1	0	0	1	1
Thrombosis	1	1	0	0	1	1
Varicose vein	1	1	0	0	1	1

11.3 Serious Adverse Events with Onset On or After Randomization

This section summarizes SAEs reported with onset date on or after the randomization date and before month 12 visit date, by blinded armcode and overall. There is 2 SAE reported before the randomization.

11.3.1 SAE Overall Summary

	**AX** (N=148)	**SBR** (N=148)	**Overall** (N=296)	P.value
Number of SAE events	23	23	46	
Number of subjects who experienced at least one SAE	20	17	37	
Percent of subjects who experienced at least one SAE	13.51%	11.49%	12.5%	0.8714

P.value is from Fisher's exact test comparing the proportions of subjects with SAE between the groups.

11.3.2 SAE by severity

	AX	SBR	Total
Mild	0 (0%)	2 (8.7%)	2 (4.35%)
Moderate	10 (43.48%)	6 (26.09%)	16 (34.78%)
Severe	13 (56.52%)	15 (65.22%)	28 (60.87%)
Total	23 (100%)	23 (100.01%)	46 (100%)

11.3.3 SAE by relationship to investigational product

	AX	SBR	Total
Definitely	0 (0%)	0 (0%)	0 (0%)
Probably	0 (0%)	0 (0%)	0 (0%)
Possibly	2 (8.7%)	0 (0%)	2 (4.35%)
Unlikely	1 (4.35%)	10 (43.48%)	11 (23.91%)
Unrelated	17 (73.91%)	13 (56.52%)	30 (65.22%)
Prior to initial exposure to intervention	3 (13.04%)	0 (0%)	3 (6.52%)
Total	23 (100%)	23 (100%)	46 (100%)

11.3.4 Approved MedDRA Coded SAEs

Due to the real-time data entry, there may be SAEs that have not been MedDRA coded at the time of data freeze. Only coded SAEs which have been approved are included in this part.

Number of SAEs summarized by MedDRA SOC

	AX	SBR	Total
Cardiac disorders	4	3	7
Gastrointestinal disorders	0	1	1
Infections and infestations	5	4	9
Injury, poisoning and procedural complications	4	2	6
Investigations	0	2	2
Neoplasms benign, malignant and unspecified (incl cysts and polyps)	1	1	2
Nervous system disorders	2	4	6
Psychiatric disorders	2	0	2
Renal and urinary disorders	0	1	1
Respiratory, thoracic and mediastinal disorders	0	1	1
Surgical and medical procedures	4	4	8
Vascular disorders	1	0	1
Total	23	23	46

CONFIDENTIALITY STATEMENT: The information contained in this document is confidential and may not be reproduced or otherwise distributed without the approval of the Biostatistics Research Center Directors.

Number of participants with at least one SAE summarized by MedDRA SOC

	AX (N=148)	SBR (N=148)	Overall (N=296)
Cardiac disorders	4 (3%)	3 (2%)	7 (2%)
Gastrointestinal disorders	0 (0%)	1 (1%)	1 (0%)
Infections and infestations	5 (3%)	4 (3%)	9 (3%)
Injury, poisoning and procedural complications	4 (3%)	2 (1%)	6 (2%)
Investigations	0 (0%)	2 (1%)	2 (1%)
Neoplasms benign, malignant and unspecified (incl cysts and polyps)	1 (1%)	1 (1%)	2 (1%)
Nervous system disorders	2 (1%)	4 (3%)	6 (2%)
Psychiatric disorders	2 (1%)	0 (0%)	2 (1%)
Renal and urinary disorders	0 (0%)	1 (1%)	1 (0%)
Respiratory, thoracic and mediastinal disorders	0 (0%)	1 (1%)	1 (0%)
Surgical and medical procedures	4 (3%)	4 (3%)	8 (3%)
Vascular disorders	1 (1%)	0 (0%)	1 (0%)

CONFIDENTIALITY STATEMENT: The information contained in this document is confidential and may not be reproduced or otherwise distributed without the approval of the Biostatistics Research Center Directors.

Number of SAEs summarized by MedDRA SOC and PT

	AX		SBR		Overall	
	Event	Subj	Event	Subj	Event	Subj
CARDIAC DISORDERS	4	4	3	3	7	7
Atrial fibrillation	0	0	1	1	1	1
Cardiac arrest	1	1	1	1	2	2
Mitral valve incompetence	0	0	1	1	1	1
Myocardial infarction	1	1	0	0	1	1
Presyncope	1	1	0	0	1	1
Tachycardia	1	1	0	0	1	1
GASTROINTESTINAL DISORDERS	0	0	1	1	1	1
Volvulus	0	0	1	1	1	1
INFECTIONS AND INFESTATIONS	5	5	4	4	9	9
Abdominal abscess	0	0	1	1	1	1
Appendicitis	1	1	0	0	1	1
Cellulitis	1	1	2	2	3	3
Cystitis	0	0	1	1	1	1
Pneumonia	2	2	0	0	2	2
Urinary tract infection	1	1	0	0	1	1
INJURY, POISONING AND PROCEDURAL COMPLICATIONS	4	4	2	2	6	6
Fall	1	1	0	0	1	1
Hip fracture	1	1	1	1	2	2
Humerus fracture	1	1	0	0	1	1
Neuroleptic malignant syndrome	1	1	0	0	1	1
Road traffic accident	0	0	1	1	1	1
INVESTIGATIONS	0	0	2	2	2	2
Haemoglobin decreased	0	0	1	1	1	1
Troponin increased	0	0	1	1	1	1
NEOPLASMS BENIGN, MALIGNANT AND UNSPECIFIED (INCL CYSTS AND POLYPS)	1	1	1	1	2	2
Lung carcinoma cell type unspecified stage IV	0	0	1	1	1	1
Meningioma	1	1	0	0	1	1
NERVOUS SYSTEM DISORDERS	2	2	4	4	6	6
Cerebrovascular accident	1	1	0	0	1	1
Dizziness	0	0	1	1	1	1
Haemorrhagic stroke	0	0	1	1	1	1
Paraesthesia	0	0	1	1	1	1
Transient ischaemic attack	0	0	1	1	1	1
Vertebral artery stenosis	1	1	0	0	1	1
PSYCHIATRIC DISORDERS	2	2	0	0	2	2
Delirium	1	1	0	0	1	1
Suicidal ideation	1	1	0	0	1	1
RENAL AND URINARY DISORDERS	0	0	1	1	1	1
Acute kidney injury	0	0	1	1	1	1

	AX		SBR		Overall	
	Event	Subj	Event	Subj	Event	Subj
RESPIRATORY, THORACIC AND MEDIASTINAL DISORDERS	0	0	1	1	1	1
Pulmonary congestion	0	0	1	1	1	1
SURGICAL AND MEDICAL PROCEDURES	4	4	4	4	8	8
Cardiac ablation	0	0	2	2	2	2
Cholecystectomy	1	1	0	0	1	1
Hip arthroplasty	1	1	0	0	1	1
Intervertebral disc operation	1	1	0	0	1	1
Joint arthroplasty	0	0	1	1	1	1
Renal tumour excision	0	0	1	1	1	1
Umbilical hernia repair	1	1	0	0	1	1
VASCULAR DISORDERS	1	1	0	0	1	1
Artery dissection	1	1	0	0	1	1

11.4 Deaths

	AX	SBR	Overall	
	(N=148)	(N=148)	(N=296)	P.value
Number of Deaths	1	2	3	
Percent of Deaths	0.68%	1.35%	1.01%	>0.9999

P.value is from Fisher's exact test comparing the proportions of death between the groups.

Listing of Death

PID	ARM	RANDDATE	DEATHDATE	EVENT	RELATION	SOC	PT
EXE0290916	SBR	2019-12-05 08:49:51.139947	04/03/2021	Cardiac arrest-fatal	Unrelated	Cardiac disorders	Cardiac arrest
EXE1370106	SBR	2017-11-20 13:08:47.871147	10/19/2018	DEATH S/P HEMOR-RHAGIC STROKE	Unrelated	Nervous system disorders	Haemorrhagic stroke
EXE1370570	AX	2019-04-09 11:50:32.195594	09/01/2020	Chest pain/hospitalization/death	Unrelated	Cardiac disorders	Cardiac arrest

CONFIDENTIALITY STATEMENT: The information contained in this document is confidential and may not be reproduced or otherwise distributed without the approval of the Biostatistics Research Center Directors.

11.5 Hospitalizations Reported as the Reason for a SAE

	AX	SBR	Overall	
	(N=148)	(N=148)	(N=296)	P.value
Number of Hospitalizations	20	18	38	
Number of subjects who hospitalized at least once	19	13	32	
Percent of subjects who hospitalized at least once	12.84%	8.78%	10.81%	0.5423

P.value is from Fisher's exact test comparing the proportions of subjects with hospitalization between the groups.

Listing of Hospitalizations

PID	ARM	RANDDATE	ADMITDT	ADMITDIAG	DISCHDT	DISCHDIAG
EXE0130411	SBR	2018-06-27 12:43:25.792695	04/15/2019	dizziness	04/16/2019	
EXE0130678	AX	2019-06-26 09:26:31.595263	10/19/2020	Myocardial Infarction	10/22/2020	Status Post Stent Placement
EXE0230406	AX	2018-06-25 07:51:48.327528	10/07/2018	hemoptysis	10/11/2018	pneumonia
EXE0230856	AX	2019-09-13 07:55:49.021829	11/24/2019	Broken Hip (right)	11/28/2019	Broken Hip(right) with partial hip replacement
EXE0290506	AX	2019-02-27 14:37:05.785546	07/16/2020	Comminuted fracture of the left humerus head and neck	07/17/2020	Comminuted fracture of the left humerus head and neck
EXE0290916	SBR	2019-12-05 08:49:51.139947	02/07/2021	Unknown	03/03/2021	Small bowel obstruction with cecal volvulus
EXE0290916	SBR	2019-12-05 08:49:51.139947	03/21/2021	Severe sepsis		
EXE0290916	SBR	2019-12-05 08:49:51.139947	03/08/2021	Unknown	03/15/2021	Intraabdominal abscess and wound dihiscence
EXE0320582	AX	2019-03-28 09:51:05.63263	08/21/2019	acute abdominal pain	08/22/2019	appendicitis with lapanascopic appendectomy
EXE0410168	SBR	2017-12-15 07:36:06.243766	09/07/2018	Cellulitis of right lower extremity	09/11/2018	Cellulitis of right lower extremity
EXE0410168	SBR	2017-12-15 07:36:06.243766	05/02/2018	Numbness	05/03/2018	Paresthesia
EXE0410171	AX	2018-03-13 07:15:27.594371	05/07/2018	Aute delirium	05/12/2018	Neuroleptic malignant syndrome
EXE0410578	SBR	2019-01-18 09:07:26.918338	05/13/2019	fatigue and abnormal troponins in setting of known CAD	05/14/2019	abnormal troponins in setting of known CAD
EXE1270422	AX	2018-10-12 06:48:31.209643	05/06/2019	Arterial dissection	05/08/2019	Arterial dissection
EXE1270656	AX	2019-07-25 04:44:21.504613	09/24/2019	multiple intracranial meningiomas	09/27/2019	multiple intracranial meningiomas
EXE1270896	AX	2019-11-19 12:50:00.289972	06/21/2020	Dizziness	06/22/2020	Vertebral artery stenosis
EXE1370006	SBR	2016-11-15 05:48:51.722799	09/07/2017	TUMOR REMOVAL ON LEFT KIDNEY	09/10/2017	TUMOR REMOVAL WITH PARTIAL LEFT NEPHRECTOMY

PID	ARM	RANDDATE	ADMITDT	ADMITDIAG	DISCHDT	DISCHDIAG
EXE1370009	SBR	2017-04-14 05:47:48.513865	08/05/2017	Irregular heart rate:Atrial Fibrullation	08/08/2017	Atrial Fibrillation
EXE1370013	AX	2017-02-27 10:32:39.997516	10/15/2017	ACUTE DELIRIUM DUE TO DEHYDRATION, SLEEP DEPRIVATION, DISORIENTATION FROM BEING LOST AND POSSIBLE UTI.		DEHYDRATION AND UTI.
EXE1370153	AX	2017-11-08 04:42:14.843699	12/28/2017	Tachycardia		
EXE1370512	SBR	2018-11-15 08:27:11.434365	11/21/2018	DX RIGHT HIP		
EXE1370545	AX	2018-12-19 06:42:09.91827	02/13/2019	PAIN LEFT ARM AND HAND	02/14/2019	S/P SURGERY-ANTERIOR CERVICAL DISCECTOMY WITH FUSION, SPACERS AND PLATES AT C-6 AND C7 LEVEL.
EXE1370570	AX	2019-04-09 11:50:32.195594	08/04/2020	Possible stroke	08/06/2020	Stroke
EXE1370570	AX	2019-04-09 11:50:32.195594	08/30/2020	Chest Pain	09/01/2020	Cardiac Arrest/death
EXE1370612	AX	2019-04-12 12:42:49.688717	07/21/2019	Chest Pain; cholecystectomy	07/22/2019	S/P cholecystectomy
EXE1370621	AX	2019-04-02 12:15:25.514691	03/09/2020	UMBILICAL HERNIA	03/12/2020	SURGICAL REPAIR OF UMBILICAL HERNIA
EXE1370647	SBR	2019-05-01 14:05:28.064633	12/09/2019	Atrial Fibrillation, heart ablation procedure	12/10/2019	Atrial Fibrillation; S/P successful radiofrequency ablation
EXE1370647	SBR	2019-05-01 14:05:28.064633	04/08/2020	Mitral valve insufficiency and patent foreman ovale	04/11/2020	Mitral valve repair and patent foreman ovale closure
EXE1370661	SBR	2019-05-13 08:50:18.355272	11/17/2019	MVA, chest pain	11/18/2019	Fractured sternum, 2 fractured ribs, and multiple contusions
EXE1370753	AX	2019-09-10 10:28:48.954946	05/14/2020	Osteoarthritis of right hip	05/15/2020	Total right hip replacement
EXE1450119	SBR	2018-01-09 10:54:06.457726	07/24/2018	TIA, Hypertension		TIA, Hypertension
EXE1450138	SBR	2018-01-18 12:03:01.089218	04/09/2018	Cellulitis leg	04/10/2018	
EXE1450872	AX	2020-01-22 13:52:39.829619	10/31/2020	Fall & Hip Discomfort	10/31/2020	Hip Fracture
EXE1450951	SBR	2020-01-22 10:58:17.851959	06/14/2021	Atrial Fibrillation	06/15/2021	Atrial Fibrillation
EXE1450951	SBR	2020-01-22 10:58:17.851959	08/18/2021	Symptomatic pulmonary vein stenosis after AF ablation	08/18/2021	Symptomatic pulmonary vein stenosis after AF ablation
EXE1530499	SBR	2018-10-02 07:56:03.32545	02/–/2019	Low Hb count	02/–/2019	stable
EXE1530922	AX	2019-11-12 08:49:46.734071	09/24/2020	High WBC count	09/27/2020	Pneumonia
EXE3020792	AX	2019-08-02 10:23:48.25831	10/12/2020	Cellulitis	10/14/2020	Cellulitis

CONFIDENTIALITY STATEMENT: The information contained in this document is confidential and may not be reproduced or otherwise distributed without the approval of the Biostatistics Research Center Directors.

12 Safety in the Extension Period

12.1 Adverse Events in the Extension Period

This section summarizes AEs for randomized subjects with onset date in the extension period (on or after month 12 visit), by blinded armcode and overall. Randomized subjects participated in the extension period are those who have on or after month 12 visits. Detailed AE listings (including uncoded AEs) are provided in Addendum Report 1.

12.1.1 AE Overall Summary

	**AX**	**SBR**	**Overall**	
	(N=115)	(N=117)	(N=232)	P.value
Number of AE events	90	54	144	
Number of subjects who experienced at least one AE	42	37	79	
Percent of subjects who experienced at least one AE	36.52%	31.62%	34.05%	0.489

P.value is from Fisher's exact test comparing the proportions of subjects with AE between the groups.

12.1.2 AE by Severity

	AX	SBR	Total	Pvalue
Mild	40 (44.44%)	31 (57.41%)	71 (49.31%)	Not Tested
Moderate	36 (40%)	21 (38.89%)	57 (39.58%)	
Severe	14 (15.56%)	2 (3.7%)	16 (11.11%)	
Total	90 (100%)	54 (100%)	144 (100%)	

12.1.3 AE by Relationship to Investigational Product

	AX	SBR	Total	Pvalue
Definitely	4 (4.44%)	0 (0%)	4 (2.78%)	Not Tested
Probably	0 (0%)	1 (1.85%)	1 (0.69%)	
Possibly	9 (10%)	1 (1.85%)	10 (6.94%)	
Unlikely	18 (20%)	7 (12.96%)	25 (17.36%)	
Unrelated	59 (65.56%)	44 (81.48%)	103 (71.53%)	
Prior to initial exposure to intervention	0 (0%)	1 (1.85%)	1 (0.69%)	
Total	90 (100%)	54 (99.99%)	144 (99.99%)	

12.1.4 Approved MedDRA Coded AEs

Due to the real-time data entry, there may be AEs that have not been MedDRA coded at the time of data freeze. Only coded AEs that have been approved are included in this part.

Number of AEs summarized by MedDRA SOC

	AX	SBR	Total
Cardiac disorders	1	1	2
Ear and labyrinth disorders	2	1	3
Eye disorders	1	1	2
Gastrointestinal disorders	3	6	9
General disorders and administration site conditions	4	0	4
Infections and infestations	9	3	12
Injury, poisoning and procedural complications	21	11	32
Investigations	10	6	16
Metabolism and nutrition disorders	1	0	1
Musculoskeletal and connective tissue disorders	14	3	17
Neoplasms benign, malignant and unspecified (incl cysts and polyps)	1	1	2
Nervous system disorders	5	6	11
Psychiatric disorders	1	0	1
Renal and urinary disorders	2	1	3
Respiratory, thoracic and mediastinal disorders	1	1	2
Skin and subcutaneous tissue disorders	0	1	1
Surgical and medical procedures	6	10	16
Vascular disorders	8	2	10
Total	90	54	144

CONFIDENTIALITY STATEMENT: The information contained in this document is confidential and may not be reproduced or otherwise distributed without the approval of the Biostatistics Research Center Directors.

Number of participants with at least one AE summarized by MedDRA SOC

	AX (N=115)	SBR (N=117)	Overall (N=232)
Cardiac disorders	1 (1%)	1 (1%)	2 (1%)
Ear and labyrinth disorders	2 (2%)	1 (1%)	3 (1%)
Eye disorders	1 (1%)	1 (1%)	2 (1%)
Gastrointestinal disorders	3 (3%)	5 (4%)	8 (3%)
General disorders and administration site conditions	4 (3%)	0 (0%)	4 (2%)
Infections and infestations	7 (6%)	3 (3%)	10 (4%)
Injury, poisoning and procedural complications	14 (12%)	9 (8%)	23 (10%)
Investigations	4 (3%)	6 (5%)	10 (4%)
Metabolism and nutrition disorders	1 (1%)	0 (0%)	1 (0%)
Musculoskeletal and connective tissue disorders	12 (10%)	3 (3%)	15 (6%)
Neoplasms benign, malignant and unspecified (incl cysts and polyps)	1 (1%)	1 (1%)	2 (1%)
Nervous system disorders	5 (4%)	6 (5%)	11 (5%)
Psychiatric disorders	1 (1%)	0 (0%)	1 (0%)
Renal and urinary disorders	2 (2%)	1 (1%)	3 (1%)
Respiratory, thoracic and mediastinal disorders	1 (1%)	1 (1%)	2 (1%)
Skin and subcutaneous tissue disorders	0 (0%)	1 (1%)	1 (0%)
Surgical and medical procedures	5 (4%)	9 (8%)	14 (6%)
Vascular disorders	6 (5%)	2 (2%)	8 (3%)

CONFIDENTIALITY STATEMENT: The information contained in this document is confidential and may not be reproduced or otherwise distributed without the approval of the Biostatistics Research Center Directors.

Number of AEs summarized by MedDRA SOC and PT

	AX		SBR		Overall	
	Event	Subj	Event	Subj	Event	Subj
CARDIAC DISORDERS	1	1	1	1	2	2
Atrial fibrillation	1	1	0	0	1	1
Bradycardia	0	0	1	1	1	1
EAR AND LABYRINTH DISORDERS	2	2	1	1	3	3
Vertigo	2	2	1	1	3	3
EYE DISORDERS	1	1	1	1	2	2
Eye irritation	0	0	1	1	1	1
Photopsia	1	1	0	0	1	1
GASTROINTESTINAL DISORDERS	3	3	6	5	9	8
Abdominal discomfort	0	0	1	1	1	1
Abdominal pain upper	1	1	0	0	1	1
Diarrhoea	0	0	2	2	2	2
Dyspepsia	0	0	1	1	1	1
Haemorrhoids	1	1	0	0	1	1
Inguinal hernia	0	0	1	1	1	1
Intestinal obstruction	0	0	1	1	1	1
Vomiting	1	1	0	0	1	1
GENERAL DISORDERS AND ADMINISTRATION SITE CONDITIONS	4	4	0	0	4	4
Chest pain	1	1	0	0	1	1
Fatigue	1	1	0	0	1	1
Gait disturbance	1	1	0	0	1	1
Oedema peripheral	1	1	0	0	1	1
INFECTIONS AND INFESTATIONS	9	7	3	3	12	10
Corona virus infection	2	2	1	1	3	3
Gastroenteritis salmonella	1	1	0	0	1	1
Gingivitis	0	0	1	1	1	1
Nasopharyngitis	1	1	0	0	1	1
Pneumonia	1	1	0	0	1	1
Upper respiratory tract infection	1	1	0	0	1	1
Urinary tract infection	2	1	1	1	3	2
Wound infection	1	1	0	0	1	1
INJURY, POISONING AND PROCEDURAL COMPLICATIONS	21	14	11	9	32	23
Contusion	1	1	1	1	2	2
Fall	9	7	8	7	17	14
Femur fracture	1	1	0	0	1	1
Foot fracture	0	0	1	1	1	1
Head injury	0	0	1	1	1	1
Injection site pain	1	1	0	0	1	1
Laceration	1	1	0	0	1	1
Ligament sprain	2	2	0	0	2	2

CONFIDENTIALITY STATEMENT: The information contained in this document is confidential and may not be reproduced or otherwise distributed without the approval of the Biostatistics Research Center Directors.

	AX		SBR		Overall	
	Event	Subj	Event	Subj	Event	Subj
Limb injury	1	1	0	0	1	1
Multiple fractures	1	1	0	0	1	1
Muscle strain	1	1	0	0	1	1
Patella fracture	1	1	0	0	1	1
Post lumbar puncture syndrome	1	1	0	0	1	1
Skin abrasion	1	1	0	0	1	1
INVESTIGATIONS	10	4	6	6	16	10
Biopsy bone marrow	1	1	0	0	1	1
Blood creatinine increased	1	1	0	0	1	1
Blood glucose increased	1	1	0	0	1	1
Blood pressure increased	0	0	1	1	1	1
Blood urea increased	1	1	0	0	1	1
Blood urine present	1	1	0	0	1	1
Bone density decreased	1	1	0	0	1	1
Cardiac murmur	0	0	1	1	1	1
Creatinine urine abnormal	0	0	1	1	1	1
Glycosylated haemoglobin increased	2	2	0	0	2	2
Hepatic enzyme increased	0	0	1	1	1	1
International normalised ratio increased	1	1	0	0	1	1
Low density lipoprotein increased	0	0	1	1	1	1
Prothrombin time prolonged	1	1	0	0	1	1
Weight decreased	0	0	1	1	1	1
METABOLISM AND NUTRITION DISORDERS	1	1	0	0	1	1
Hypercalcaemia	1	1	0	0	1	1
MUSCULOSKELETAL AND CONNECTIVE TISSUE DISORDERS	14	12	3	3	17	15
Arthralgia	4	4	2	2	6	6
Arthritis	1	1	0	0	1	1
Back pain	4	4	1	1	5	5
Exostosis	1	1	0	0	1	1
Muscular weakness	1	1	0	0	1	1
Osteoarthritis	1	1	0	0	1	1
Pain in extremity	1	1	0	0	1	1
Spinal pain	1	1	0	0	1	1
NEOPLASMS BENIGN, MALIGNANT AND UNSPECIFIED (INCL CYSTS AND POLYPS)	1	1	1	1	2	2
Breast cancer	0	0	1	1	1	1
Neoplasm malignant	1	1	0	0	1	1
NERVOUS SYSTEM DISORDERS	5	5	6	6	11	11
Amyotrophic lateral sclerosis	0	0	1	1	1	1
Cerebral microhaemorrhage	0	0	1	1	1	1
Colloid brain cyst	0	0	1	1	1	1

CONFIDENTIALITY STATEMENT: The information contained in this document is confidential and may not be reproduced or otherwise distributed without the approval of the Biostatistics Research Center Directors.

	AX		SBR		Overall	
	Event	Subj	Event	Subj	Event	Subj
Confusional state	1	1	0	0	1	1
Dizziness	0	0	1	1	1	1
Paraesthesia	1	1	0	0	1	1
Parkinsonism	0	0	1	1	1	1
Reflexes abnormal	0	0	1	1	1	1
Syncope	1	1	0	0	1	1
Transient global amnesia	1	1	0	0	1	1
Tremor	1	1	0	0	1	1
PSYCHIATRIC DISORDERS	1	1	0	0	1	1
Insomnia	1	1	0	0	1	1
RENAL AND URINARY DISORDERS	2	2	1	1	3	3
Chronic kidney disease	1	1	1	1	2	2
Ureteric obstruction	1	1	0	0	1	1
RESPIRATORY, THORACIC AND MEDIASTINAL DISORDERS	1	1	1	1	2	2
Dyspnoea	1	1	1	1	2	2
SKIN AND SUBCUTANEOUS TISSUE DISORDERS	0	0	1	1	1	1
Rash	0	0	1	1	1	1
SURGICAL AND MEDICAL PROCEDURES	6	5	10	9	16	14
Arthrodesis	0	0	1	1	1	1
Arthroscopic surgery	1	1	1	1	2	2
Cancer surgery	1	1	1	1	2	2
Inguinal hernia repair	0	0	2	2	2	2
Knee arthroplasty	1	1	1	1	2	2
Lymphadenectomy	0	0	1	1	1	1
Medical device removal	1	1	0	0	1	1
Micrographic skin surgery	0	0	2	1	2	1
Sinus operation	1	1	0	0	1	1
Skin neoplasm excision	1	1	0	0	1	1
Transurethral prostatectomy	0	0	1	1	1	1
VASCULAR DISORDERS	8	6	2	2	10	8
Accelerated hypertension	2	1	0	0	2	1
Blood pressure fluctuation	0	0	1	1	1	1
Dizziness	2	1	0	0	2	1
Haemorrhage	0	0	1	1	1	1
Hypertension	2	2	0	0	2	2
Hypotension	1	1	0	0	1	1
Thrombosis	1	1	0	0	1	1

CONFIDENTIALITY STATEMENT: The information contained in this document is confidential and may not be reproduced or otherwise distributed without the approval of the Biostatistics Research Center Directors.

12.2 Serious Adverse Events with onset date in the extension period

This section summarizes SAEs reported with onset date in the extension period (on or after month 12 visit date), by blinded armcode and overall.

12.2.1 SAE Overall Summary

	**AX** (N=115)	**SBR** (N=117)	**Overall** (N=232)	P.value
Number of SAE events	10	2	12	
Number of subjects who experienced at least one SAE	7	2	9	
Percent of subjects who experienced at least one SAE	4.73%	1.35%	3.04%	0.2349

P.value is from Fisher's exact test comparing the proportions of subjects with SAE between the groups.

12.2.2 SAE by severity

	AX	SBR	Total	Pvalue
Mild	1 (10%)	0 (0%)	1 (8.33%)	Not Tested
Moderate	0 (0%)	1 (50%)	1 (8.33%)	
Severe	9 (90%)	1 (50%)	10 (83.33%)	
Total	10 (100%)	2 (100%)	12 (99.99%)	

12.2.3 SAE by relationship to investigational product

	AX	SBR	Total	Pvalue
Definitely	0 (0%)	0 (0%)	0 (0%)	Not Tested
Probably	0 (0%)	0 (0%)	0 (0%)	
Possibly	0 (0%)	0 (0%)	0 (0%)	
Unlikely	2 (20%)	0 (0%)	2 (16.67%)	
Unrelated	8 (80%)	2 (100%)	10 (83.33%)	
Prior to initial exposure to intervention	0 (0%)	0 (0%)	0 (0%)	
Total	10 (100%)	2 (100%)	12 (100%)	

CONFIDENTIALITY STATEMENT: The information contained in this document is confidential and may not be reproduced or otherwise distributed without the approval of the Biostatistics Research Center Directors.

12.2.4 Approved MedDRA Coded SAEs

Due to the real-time data entry, there may be SAEs that have not been MedDRA coded at the time of data freeze. Only coded SAEs which have been approved are included in this part.

Number of SAEs summarized by MedDRA SOC

	AX	SBR	Total
Cardiac disorders	0	1	1
Infections and infestations	2	0	2
Injury, poisoning and procedural complications	1	0	1
Musculoskeletal and connective tissue disorders	3	0	3
Neoplasms benign, malignant and unspecified (incl cysts and polyps)	1	0	1
Nervous system disorders	1	0	1
Surgical and medical procedures	1	1	2
Vascular disorders	1	0	1
Total	10	2	12

Number of participants with at least one SAE summarized by MedDRA SOC

	**AX** (N=115)	**SBR** (N=117)	**Overall** (N=232)
Cardiac disorders	0 (0%)	1 (1%)	1 (0%)
Infections and infestations	2 (2%)	0 (0%)	2 (1%)
Injury, poisoning and procedural complications	1 (1%)	0 (0%)	1 (0%)
Musculoskeletal and connective tissue disorders	2 (2%)	0 (0%)	2 (1%)
Neoplasms benign, malignant and unspecified (incl cysts and polyps)	1 (1%)	0 (0%)	1 (0%)
Nervous system disorders	1 (1%)	0 (0%)	1 (0%)
Surgical and medical procedures	1 (1%)	1 (1%)	2 (1%)
Vascular disorders	1 (1%)	0 (0%)	1 (0%)

CONFIDENTIALITY STATEMENT: The information contained in this document is confidential and may not be reproduced or otherwise distributed without the approval of the Biostatistics Research Center Directors.

Number of SAEs summarized by MedDRA SOC and PT

	AX		SBR		Overall	
	Event	Subj	Event	Subj	Event	Subj
CARDIAC DISORDERS	0	0	1	1	1	1
Bradycardia	0	0	1	1	1	1
INFECTIONS AND INFESTATIONS	2	2	0	0	2	2
Gastroenteritis salmonella	1	1	0	0	1	1
Pneumonia	1	1	0	0	1	1
INJURY, POISONING AND PROCEDURAL COMPLICATIONS	1	1	0	0	1	1
Fall	1	1	0	0	1	1
MUSCULOSKELETAL AND CONNECTIVE TISSUE DISORDERS	3	2	0	0	3	2
Arthralgia	1	1	0	0	1	1
Back pain	1	1	0	0	1	1
Muscular weakness	1	1	0	0	1	1
NEOPLASMS BENIGN, MALIGNANT AND UNSPECIFIED (INCL CYSTS AND POLYPS)	1	1	0	0	1	1
Neoplasm malignant	1	1	0	0	1	1
NERVOUS SYSTEM DISORDERS	1	1	0	0	1	1
Confusional state	1	1	0	0	1	1
SURGICAL AND MEDICAL PROCEDURES	1	1	1	1	2	2
Inguinal hernia repair	0	0	1	1	1	1
Skin neoplasm excision	1	1	0	0	1	1
VASCULAR DISORDERS	1	1	0	0	1	1
Hypotension	1	1	0	0	1	1

CONFIDENTIALITY STATEMENT: The information contained in this document is confidential and may not be reproduced or otherwise distributed without the approval of the Biostatistics Research Center Directors.

12.3 Deaths

	AX	SBR	Overall	
	(N=115)	(N=117)	(N=232)	P.value
Number of Deaths	1	0	1	
Percent of Deaths	0.87%	0%	0.43%	0.4978

P.value is from Fisher's exact test comparing the proportions of death between the groups.

Listing of Death

PID	ARM	RANDDATE	DEATHDATE	EVENT	RELATION	SOC	PT
EXE0210611	AX	2019-05-15 10:30:35.503298	05/31/2021	Cancer of unknown type	Unrelated	Neoplasms benign, malignant and unspecified (incl cysts and polyps)	Neoplasm malignant

12.4 Hospitalizations Reported as the Reason for a SAE

	AX	SBR	Overall	
	(N=115)	(N=117)	(N=232)	P.value
Number of Hospitalizations	8	2	10	
Number of subjects who hospitalized at least once	6	2	8	
Percent of subjects who hospitalized at least once	5.22%	1.71%	3.45%	0.3333

P.value is from Fisher's exact test comparing the proportions of subjects with hospitalization between the groups.

Listing of Hospitalizations

PID	ARM	RANDDATE	ADMITDT	ADMITDIAG	DISCHDT	DISCHDIAG
EXE0210587	SBR	2019-03-27 09:05:57.129916	02/13/2021	unknown	02/17/2021	Bradycardia
EXE0290034	AX	2017-05-09 12:54:35.177657	10/08/2018	Dehydration	10/11/2018	Salmonella gastroenteritis
EXE0290573	AX	2019-01-08 11:14:18.471621	05/15/2021	Right hip pain	05/20/2021	Pneumonia
EXE0330485	AX	2018-11-29 12:48:31.746577	08/04/2020	Basal Cell Carcinoma Removal	08/05/2020	Basal Cell Carcinoma Removal
EXE1270561	SBR	2019-04-05 11:50:11.341678	12/27/2020	Inguinal hernia laparoscopic repair	12/28/2020	Inguinal hernia laparoscopic repair
EXE1270904	AX	2020-01-09 09:14:02.244533	08/10/2021	Hip + Leg pain	08/11/2021	Hip + Leg pain
EXE1370664	AX	2019-08-06 12:04:47.443981	05/05/2021	1) Severe spinal stenosis, instability, and thoracic myelopathy secondary to L1 burst fracture with delayed healing; 2) L3-4 severe spinal stenosis; 3) L4-5 severe spinal stenosis	05/07/2021	SPINAL FUSION POSTERIOR T12-L2 PSF and L3-4, L4-5
EXE1370664	AX	2019-08-06 12:04:47.443981	06/04/2021	Altered mental status	06/16/2021	Debility, multiple myeloma
EXE1370664	AX	2019-08-06 12:04:47.443981	07/01/2021	Weakness bilateral lower extremities, pain	07/02/2021	Weakness bilateral lower extremities, pain
EXE1830050	AX	2017-08-03 08:15:36.539198	11/11/2018	hypotension, syncope	11/15/2018	hypotension

CONFIDENTIALITY STATEMENT: The information contained in this document is confidential and may not be reproduced or otherwise distributed without the approval of the Biostatistics Research Center Directors.

13 Protocol Deviations

This section summarizes the protocol deviations by deviation type for randomized subjects. 272 patients had 1019 deviations in total.

	AX	SBR	Total	Pvalue
Informed Consent	4 (0.81%)	1 (0.19%)	5 (0.49%)	Not Tested
Inclusion Criteria	18 (3.64%)	20 (3.81%)	38 (3.73%)	
Exclusion Criteria	3 (0.61%)	2 (0.38%)	5 (0.49%)	
Randomization	22 (4.45%)	30 (5.71%)	52 (5.1%)	
Exercise Intervention	29 (5.87%)	23 (4.38%)	52 (5.1%)	
Protocol Procedures	271 (54.86%)	297 (56.57%)	568 (55.74%)	
Excluded Concurrent Medications	0 (0%)	1 (0.19%)	1 (0.1%)	
Investigator Site Personnel	3 (0.61%)	6 (1.14%)	9 (0.88%)	
Other	144 (29.15%)	145 (27.62%)	289 (28.36%)	
Total	494 (100%)	525 (99.99%)	1019 (99.99%)	

CONFIDENTIALITY STATEMENT: The information contained in this document is confidential and may not be reproduced or otherwise distributed without the approval of the Biostatistics Research Center Directors.

14 Appendix

14.1 Listing of Inclusion/Exclusion Criteria (Protocol V2)

Inclusion Criteria
1. Age between 65 and 89 years old, inclusive
2. Diagnosis of single or multi-domain amnestic MCI using clinical criteria as per NIA/Alzheimer's Association Guidelines.[86]
3. MMSE: ≥24 for participants with 13 or more years of education; ≥22 for participants with 12 or fewer years of education
4. CDR =0.5
5. Impaired delayed verbal recall as indicated by scores meeting at least ONE of the following criteria:
    a. Logical Memory II ≤8
    b. Auditory Verbal Learning Test, Trial 7 ≤4
6. Speaks English fluently
7. Visual and auditory acuity adequate for cognitive testing
8. Completed at least 6 years of formal education or work history sufficient to exclude mental retardation
9. Has an informant who knows the participant well, has at least weekly contact, and is available to accompany the participant to clinic visits
10. Sedentary or underactive, determined by responses to the staff-administered Telephone Assessment of Physical Activity (TAPA) survey
11. Willing to be randomized to either intervention group and to complete the assigned activities as specified for 18 months
12. Willing and able to reliably travel to the identified YMCA, 4 times per week for 18 months
13. Ability to safely participate in either intervention and complete the 400 m Walk Test within 15 min without sitting or use of any assistance
14. Plans to reside in the area for at least 18 months
15. For planned travel, total time away must be no more than 2 months over the course of the study, and no more than 1 month at any one time; participants must be willing to continue the assigned exercise program if travelling out of the area for more than 1 week
16. In overall good general health with no disease or planned surgery that could interfere with study participation
17. Modified Hachinski ≤4
18. Stable use of cholinesterase inhibitors, vitamin E (up to 400 IU daily), estrogens, aspirin (81-300 mg daily), beta-blockers, or cholesterol-lowering agents for 12 weeks prior to screening (important for biomarker analyses)
19. Stable use of antidepressants lacking significant anticholinergic side effects for 4 weeks prior to screening as long as the participant does not meet DSM V criteria for major depression currently or in the last 12 months; GDS scores are to be used to inform clinical decisions but there is no specified cut-off score for inclusion
20. When applicable, willing to complete 4-week washout of psychoactive medications, including disallowed antidepressants, neuroleptics, chronic anxiolytics or sedative hypnotics, and willing to avoid these medications for the duration of the trial
21. Able to complete all baseline assessments

Exclusion Criteria
1. Any significant neurologic disease, other than MCI, including any form of dementia, Parkinson's disease, Huntington's disease, normal pressure hydrocephalus, brain tumor, progressive supranuclear palsy, seizure disorder, subdural hematoma, multiple sclerosis, or history of significant head trauma with persistent neurologic sequelae or known structural brain abnormalities
2. Sensory or musculoskeletal impairment sufficient to preclude successful and safe completion of the intervention or assessment protocols; must be able to walk safely and unassisted on a treadmill
3. Contraindications for MRI studies, including claustrophobia, metal (ferromagnetic) implants, or cardiac pacemaker
4. Brain MRI at screening shows evidence of infection, infarction, or other clinically significant focal lesions, including multiple lacunes in prefrontal or critical memory regions; inconclusive findings may be subject to review by the ADCS Imaging Core
5. History of major depression or bipolar disorder (DSM V criteria), psychotic features, agitation or behavioral problems within the last 12 months

CONFIDENTIALITY STATEMENT: The information contained in this document is confidential and may not be reproduced or otherwise distributed without the approval of the Biostatistics Research Center Directors.

6. History of schizophrenia, as per DSM V criteria
7. History of alcohol or substance abuse or dependence within the past 2 years, as per DSM V criteria
8. Currently consumes more than 3 alcoholic drinks per day
9. Clinically significant or unstable medical condition, including uncontrolled hypertension or significant cardiac, pulmonary, hematologic, renal, hepatic, gastrointestinal, endocrine, metabolic or other systemic disease in the opinion of clinic medical personnel that may put the participant at increased risk, influence the results or compromise the participant's ability to participate in the study (treated atrial fibrillation for more than 1 year or occasional premature ventricular contractions on ECG are not exclusions)
10. History in the last 6 months of myocardial infarction, coronary artery angioplasty, bypass grafting, or STENT placement
11. History in the last 3 months of transient ischemic attack or small vessel stroke (if more than 3 months, small vessel stroke with no residual effects are permitted)
12. Expected joint replacement surgery within the next 18 months
13. History within the last 5 years of a primary or recurrent malignant disease with the exception of non-melanoma skin cancers, resected cutaneous squamous cell carcinoma in situ, basal cell carcinoma, cervical carcinoma in situ, or in situ prostate cancer with normal prostate-specific antigen posttreatment
14. Hemoglobin A1c >7.0
15. Clinically significant abnormalities in screening laboratory blood tests: low B12 is exclusionary, unless follow-up labs (homocysteine [HCY] and methylmalonic acid [MMA]) indicate that it is not physiologically significant
16. Current or past use of insulin to treat type 2 diabetes (other diabetes medications are acceptable if hemoglobin A1c ≤7)
17. Current use (within 60 days of screening) of psychoactive medications including tricyclic antidepressants, antipsychotics, mood-stabilizing psychotropic agents (e.g. lithium salts), psychostimulants, opiate analgesics, antiparkinsonian medications, anticonvulsant medications (except gabapentin and pregabulin for non-seizure indications), systemic corticosteroids, or medications with significant central anticholinergic activity
18. Current use of memantine
19. Chronic use of anxiolytics or sedative hypnotics except as follows: use of benzodiazepines for treatment on an as-needed basis for insomnia or daily dosing of anxiolytics is permitted; medications must be avoided for 8 hours before clinic assessments
20. Previous or current treatment involving active immunization against amyloid
21. Previous treatment with investigational agents with anti-amyloid properties or passive immunization against amyloid are prohibited 12 months prior to screening and for the duration of the trial; treatment with other investigational agents are prohibited 3 months prior to screening and for the duration of the trial
22. For LP, current use of anticoagulants such as Coumadin or Plavix
23. For LP, current blood clotting or bleeding disorder, or significantly abnormal prothrombin time (PT) or partial thromboplastin time (PTT) at screening
24. For LP, presence of physical distortions due to spinal surgery, severe degenerative joint disease or deformity, or obesity that could interfere with CSF collection (as per investigator judgment)
25. Participants whom the PI deems otherwise ineligible

14.2 Listing of Addendum Reports

1. Addendum 1: Detailed AE Report
2. Addendum 2: Detailed SAE Report

15 Software

Statistical software R (version 4.0.5) is used *http://www.r-project.org*.

CONFIDENTIALITY STATEMENT: The information contained in this document is confidential and may not be reproduced or otherwise distributed without the approval of the Biostatistics Research Center Directors.
