# Supplementary material for: Effects of exercise on cognition and Alzheimer's biomarkers in a randomized controlled trial of adults with mild cognitive impairment: The EXERT study
Source: Alzheimers Dement. 2025 Apr 24;21(4):e14586. doi: 10.1002/alz.14586 (PMC12019696; doi:10.1002/alz.14586)
Supplement: Supplementary file 4 — Supporting Information [file ALZ-21-e14586-s001.pdf]

Protocol Title:

Therapeutic Effects of Exercise in Adults with Amnestic Mild Cognitive Impairment

Protocol Short Title:

EXERT

Protocol Number:

ADC-041-EX

Protocol Version 7.0
18NOV2021

Confidentiality Statement:
The information in this document is privileged or confidential and may not be disclosed unless such disclosure is required by applicable laws and regulations. It is the property of NIH and ADCS and should not be copied by or distributed to persons not involved in the study. Persons to whom the information is disclosed must be informed that the information is privileged or confidential and may not be further disclosed by them. These restrictions on disclosure will apply equally to all future information supplied to you, which is indicated as privileged or confidential. Any unauthorized review, use, disclosure, copying or distribution is strictly prohibited.

SIGNATURE PAGE

(SIGNATURES ON FILE AT ADCS)

________________________________ _________________

Laura Baker, PhD Date (MM/DD/YYYY)
Project Director
Professor of Internal Medicine - Geriatrics
Wake Forest University School of Medicine

________________________________ _________________

Carl Cotman, PhD Date (MM/DD/YYYY)
Project Director
Professor of Neurology & Neurobiology
University of California, Irvine

________________________________ _________________

Howard Feldman, MDCM, FRCP(C) Date (MM/DD/YYYY)
Principal Investigator
Alzheimer’s Disease Cooperative Study
University of California, San Diego

________________________________ _________________

Gabriel Leger, MD Date (MM/DD/YYYY)
Medical Monitor
Alzheimer's Disease Cooperative Study
University of California, San Diego

Sponsor: Laurie M. Ryan, PhD
National Institute on Aging
7201 Wisconsin Avenue, Suite 350
Bethesda, MD 20892-9205
ryanl@mail.nih.gov
Tel: 301-496-9350

Protocol Number: ADC-041-EX

Project Directors: Laura Baker, PhD
Wake Forest University Health Sciences
Medical Center Boulevard
Winston Salem, NC 27157-1207
ldbaker@wakehealth.edu
Tel: 336-713-8831

Carl Cotman, PhD
University of California - Irvine
School of Medicine
1113 Gillespie Building
Irvine, CA 92697
cwcotman@uci.edu
Tel: 949-824-5847

Principal Investigator, Coordination & Data Management Center Coordinating Center: Howard Feldman, MD
ADCS Director
Alzheimer's Disease Cooperative Study
University of California, San Diego
9500 Gilman Drive
La Jolla, CA 92093-0949
howardfeldman@health.ucsd.edu
Tel: (858) 246-1365

Medical Emergencies: Gabriel Leger, MD
Medical Monitor
Alzheimer's Disease Cooperative Study
gleger@health.ucsd.edu
Tel: (858) 246-2539

Version: 7.0 18NOV2021

TABLE OF CONTENTS

LIST OF ABBREVIATIONS 9
PROTOCOL SYNOPSIS 11
1.0 INTRODUCTION 13
1.1 Primary Aim 13
1.2 Secondary Aims 14
2.0 Preliminary Studies 15
2.1 Summary of Clinical Findings 15
2.2 Safety and Compliance 16
3.0 Background and significance 16
3.1 Rationale for Aerobic Exercise as a Therapeutic Intervention in Mild Cognitive Impairment 16
3.2 Rationale for Aerobic Exercise Effects on AD Pathology in the Brain 17
3.3 Rationale Supporting Selection of Primary and Secondary Cognitive Outcomes 17
3.4 Rationale for Length and Design of EXERT 18
3.5 Rationale for Population Selection 18
3.6 Rationale for Selected Exercise Dose 18
3.7 Rationale for Intervention Implementation in Community-Based Facilities 18
3.8 Rationale for Magnetic Resonance Imaging 19
3.9 Rationale for Inclusion of Actigraphy 19
3.10 Rationale for Collection of Biofluids 20
4.0 Potential Risks and benefits associated with Intervention 20
4.1 Potential Benefits 20
4.2 Potential Risks 20
5.0 Sample Size and Statistical Plan 22
5.1 Randomization 22
5.2 Power and Sample Size Determination 22
5.3 Selection of Participants to be Used in Analysis 23
5.4 Efficacy Analysis 23
5.4.1 Analysis of Primary Outcome 23
5.4.2 Analyses of Secondary Outcomes 24
5.5 Safety Analysis 25
5.6 Termination of the Trial 25

6.0 Interventions and implementation ... 25
6.1 Overview ... 25
6.2 Collaboration with the YMCA ... 26
6.2.1 YMCA Study Team ... 26
6.2.2 Interface Between the Clinic and the YMCA ... 27
6.2.3 Adverse Event Reporting by YMCA Trainers ... 27
6.3 Implementation of the Interventions ... 28
6.3.1 Aerobic Training Intervention ... 30
6.3.2 Stretching, Balance, and Range of Motion Intervention ... 31
6.4 Adherence ... 31
6.5 Safety During Exercise ... 32
6.6 Temporary Intervention Discontinuation ... 33
6.7 Reasons for Temporary Discontinuation ... 33
6.8 Timing of Temporary Discontinuation Relative to Assessment of Primary and Secondary Outcomes ... 34
6.9 Blinding ... 34
6.10 Breaking the Blind ... 35
6.11 Concomitant AD Medications ... 35
7.0 Study Population ... 35
7.1 Inclusion Criteria ... 35
7.2 Exclusion Criteria ... 36
7.3 Recruitment and Retention Strategies ... 38
7.4 Inclusion of Women and Minorities ... 38
8.0 Study Timeline ... 38
9.0 Description of study visits ... 38
9.1 Screening I (Visit 0) ... 38
9.2 Screening II (Visit 1) ... 39
9.3 Baseline (Visit 2) ... 40
9.4 Month 6 (Visit 3) ... 42
9.5 Month 12 (Visit 4) / or Early Termination ... 43
9.6 Month 18 (Visit 5) ... 45
10.0 Early intervention discontinuation ... 46
10.1 Reasons for Early Discontinuation ... 47

11.0 Study-Specific Instruments 47
11.1 EXERT Telephone Assessment of Physical Activity 47
11.2 Cognitive Evaluations 47
11.2.1 Modified Mental State Examination 47
11.2.2 Logical Memory 48
11.2.3 Auditory Verbal Learning Test 48
11.2.4 ADAS-Cog-Exec Global Composite 48
11.2.5 Executive Function Composite 49
11.2.6 Episodic Memory Composite 51
11.3 Behavioral and Functional Evaluations 51
11.3.1 Clinical Dementia Rating Scale – Sum of Boxes (described above) 51
11.3.2 ADCS-Activities of Daily Living-MCI 51
11.3.3 Behavior Rating Inventory of Executive Function – Adult Version 52
11.3.4 Neuropsychiatric Inventory 52
11.3.5 Cognitive Change Index 52
11.3.6 Geriatric Depression Scale 52
11.3.7 SF-36 52
11.3.8 EQ5D 53
11.3.9 Study Partner Self-Assessment Questionnaire 53
11.4 Modified Hachinski 53
11.5 Research Satisfaction Survey 53
11.6 Treatment Blinding Questionnaire 53
11.7 Heart Rate Monitor 54
11.8 ActiGraph Link 54
11.9 Instruments Administered by Trainers at the YMCA 54
11.9.1 Rating of Perceived Exertion 54
11.9.2 Adverse Event Checklist 54
12.0 Study–specific procedures 54
12.1 Safety Assessments 54
12.1.1 Physical and Neurological Examination 55
12.1.2 Electrocardiogram 55
12.1.3 Clinical Laboratory Evaluations 55

12.2 Biofluids.....56
12.2.1 Genetic Samples, Storage and Future Use.....56
12.2.2 Blood Collection for Biomarker Analyses and Banking.....56
12.2.3 CSF Collection for Biomarker Analyses and Banking.....56
12.3 Magnetic Resonance Imaging.....57
12.3.1 Site Qualification.....57
12.3.2 Data Acquisition.....57
12.3.3 Data Management and Quality Control.....58
12.4 400 m Walk Test.....58
12.5 Actigraphy Recordings.....59
13.0 Personnel Requirements.....59
13.1 Clinic Personnel.....59
13.2 YMCA Study Team (see Section 6.2.1).....60
14.0 Adverse Events.....61
14.1 Definition.....61
14.2 Follow-Up of AEs.....62
14.3 Reporting of Unanticipated Problems, AEs, Protocol Deviations.....62
15.0 Serious Adverse Events.....62
15.1 Definition.....62
15.2 Reporting SAEs.....62
16.0 Data and Safety Monitoring Board.....63
17.0 Recording and collection of data.....63
17.1 Case Report Form.....63
17.2 Audio / Visual Recording.....64
17.3 Study Files and Patient Source Documents.....64
18.0 Ethics and Regulatory Considerations.....64
18.1 Good Clinical Practice.....64
18.2 Institutional Review Board.....65
18.3 Informed Consent and HIPAA Compliance.....65
18.3.1 Participant Confidentiality | HIPAA.....66
18.4 Genetic Research and Storage of Genetic Material.....66
18.5 Storage of Biospecimen Samples.....66
18.6 MRI Data Storage.....67

18.7 Inclusion of Children as Participants in Research Involving Human Subjects 67
18.8 Study Monitoring 67
19.0 Audit 67
20.0 Record Retention 67
21.0 Publication Policy 68
22.0 Sharing of Final Research Data 68
23.0 Literature Cited 69
Appendix 1: Schedule of Events 77

LIST OF ABBREVIATIONS

**3MSE**	**Modified Mental Status Exam**
**Aβ**	**Beta-Amyloid**
**AD**	**Alzheimer's Disease**
**ADAS-Cog**	**Alzheimer's Disease Assessment Scale – Cognitive Subscale**
**ADAS-Cog-Exec**	**Alzheimer's Disease Assessment Scale – Cognitive and Executive Function Composite Score**
**ADCS**	**Alzheimer's Disease Cooperative Study**
**ADCS-ADL-MCI**	**Alzheimer's Disease Cooperative Study – Activities Of Daily Living – Mild Cognitive Impairment**
**ADNI**	**Alzheimer's Disease Neuroimaging Initiative**
**AE**	**Adverse Event**
**APOE/APOE4**	**Apolipoprotein/Apolipoprotein Epsilon 4**
**ASL**	**Arterial Spin Labeling**
**AVLT**	**Auditory Verbal Learning Test**
**AX**	**Aerobic Exercise**
**BDNF**	**Brain-Derived Neurotrophic Factor**
**BRIEF-A**	**Behavior Rating Inventory Of Executive Function – Adult Version**
**BPSO**	**Behavioral Pattern Separation Of Objects (Stark Task)**
**CDR**	**Clinical Dementia Rating**
**CDR-SB**	**Clinical Dementia Rating – Sum Of Boxes**
**CFR**	**Code Of Federal Regulations**
**CRF/e-CRF**	**Case Report Form/Electronic Case Report Form**
**CSF**	**Cerebrospinal Fluid**
**DET**	**Detection Task (Cogstate)**
**DCCS**	**Dimensional Change Card Sort (NIH Toolbox)**
**DNA**	**Deoxyribonucleic Acid**
**DSMB**	**Data And Safety Monitoring Board**
**DSST**	**Digit Symbol Substitution Test**
**ECG**	**Electrocardiogram**
**EDC**	**Electronic Data Capture**
**EQ5D**	**EuroQol 5-Item Health Questionnaire**
**FDA**	**Food And Drug Administration**
**FNAME**	**Face-Name Associative Memory Exam**
**fMRI**	**Functional Magnetic Resonance Imaging**
**GCP**	**Good Clinical Practice**
**GDS**	**Geriatric Depression Scale**
**GMLT**	**Groton Maze Learning Test (Cogstate)**
**HBA1C**	**Hemoglobin A1c**
**HCY**	**Homocysteine**
**HIPAA**	**Health Insurance Portability And Accountability Act**
**HR**	**Heart Rate**

**HRR**	**Heart Rate Reserve**
**ICF**	**Informed Consent Form**
**ICH**	**International Conference On Harmonisation**
**IDN**	**Identification Task (Cogstate)**
**IRB**	**Institutional Review Board**
**LP**	**Lumbar Puncture**
**MCI**	**Mild Cognitive Impairment**
**MEDDRA**	**Medical Dictionary For Regulatory Activities**
**MMA**	**Methylmalonic Acid**
**MMRM**	**Mixed Effects Model For Repeated Measures**
**MMSE**	**Mini-Mental State Examination**
**MPRAGE**	**Magnetization Prepared Rapid Gradient Echo**
**MR/MRI**	**Magnetic Resonance / Magnetic Resonance Imaging**
**NIA**	**National Institute On Aging**
**NIH**	**National Institutes Of Health**
**NPI**	**Neuropsychiatric Inventory**
**NSAID**	**Non-Steroidal Anti-Inflammatory Drugs**
**OBK**	**One Back Task (Cogstate)**
**OCL**	**One Card Learning (Cogstate)**
**OHRP**	**Office For Human Research Protections**
**PD**	**Project Director**
**PCP**	**Primary Care Provider**
**PHI**	**Protected Health Information**
**PI**	**Principal Investigator**
**PID**	**Participant Identification Number**
**RPE**	**Rating Of Perceived Exertion**
**PT**	**Prothrombin Time**
**PTT**	**Partial Thromboplastin Time**
**SAE**	**Severe Adverse Event**
**SBR**	**Stretching/Balance/Range Of Motion**
**SD**	**Standard Deviation**
**SF-36**	**36-Item Short Form Health Survey**
**sMRI**	**Structural Magnetic Resonance Imaging**
**T**	**Tesla**
**TAPA**	**Telephone Assessment Of Physical Activity**
**TEAE**	**Treatment-Emergent Adverse Event**
**TSH**	**Thyroid Stimulating Hormone**
**VEGF**	**Vascular Endothelial Growth Factor**
**WAIS-R**	**Wechsler Adult Intelligence Scale – Revised**
**Y-PM**	**YMCA Project Manager**
**Y-USA**	**YMCA Of The United States Of America**

PROTOCOL SYNOPSIS

**PROTOCOL TITLE**	Therapeutic Effects of Exercise in Adults with Amnestic Mild Cognitive Impairment
**PROJECT DIRECTORS**	Laura Baker, PhD and Carl Cotman, PhD
**STUDY SPONSOR**	National Institute on Aging (NIA)
**STUDY DESIGN**	Phase 3, multicenter, randomized single-blind study to examine the effects of aerobic exercise on cognition, functional status, whole and regional brain atrophy, whole and regional cerebral blood flow, and cerebrospinal fluid biomarkers of Alzheimer's disease in 300 adults with amnestic mild cognitive impairment (MCI)
**DURATION OF STUDY PARTICIPATION**	• Screening period of up to 4 weeks<br>• Supervised intervention duration of 12 months<br>• Unsupervised intervention extension of 6 months
**SUMMARY OF INVESTIGATIONAL INTERVENTION**	• Two interventions will be implemented: supervised and structured moderate/high intensity aerobic training vs. stretching/balance/range of motion for 12 months, followed by a 6-month unsupervised extension<br>• Moderate/high intensity aerobic exercise will involve training at 70-80% heart rate reserve for 30 min, with an additional 10 minutes for warm-up and 5 minutes for cool-down, 4 times per week, for 12 months while supervised twice per week by a study-certified YMCA Trainer; participants in the stretching/balance/range of motion group will adhere to the same timeline, but exercise at an intensity at or below 35% heart rate reserve
**SUMMARY OF KEY ELIGIBILITY CRITERIA**	• Age: 65 to 89 years (inclusive)<br>• Mini-Mental State Examination at screening: ≥24 for participants with 13 or more years of education; ≥22 for participants with 12 or fewer years of education<br>• Clinical Dementia Rating =0.5 with a memory score of 0.5 or greater at screening<br>• Profile of test scores and clinical ratings is consistent with amnestic mild cognitive impairment<br>• Modified Hachinski score ≤4
**PRIMARY OUTCOME MEASURE**	ADAS-Cog-Exec Global Composite Score: Word Recall, Delayed Word Recall, Orientation and Number Cancellation subscales of the Alzheimer's Disease Assessment Scale – Cognitive Subscale, version 13 (ADAS-Cog13); tests of executive function including Trail-Making Test A and B, Digit Symbol Substitution Test, and Category Fluency; and Clinical Dementia Rating (CDR) sum of box scores for Memory, Orientation and Judgement and Problem Solving.

**SECONDARY OUTCOME MEASURES**	Clinical Dementia Rating – Sum of Boxes, ADAS-Cog13 total score, Executive Function Composite, Episodic Memory Composite, functional status (Alzheimer's Disease Cooperative Study – Activities Of Daily Living – MCI, Behavior Rating Inventory Of Executive Function – Adult Version), mood and health-related quality of life (Neuropsychiatric Inventory, 36-Item Short Form Health Survey, EuroQol 5-Item Health Questionnaire), brain volume and perfusion using structural and functional magnetic resonance imaging, biomarkers in blood and cerebrospinal fluid, actigraphy-measured total activity and sleep integrity

1.0 INTRODUCTION

An urgent need exists to find effective treatments for Alzheimer's disease (AD) that can arrest or reverse the disease at its earliest stages. The emotional and financial burden of AD to patients, family members, and society is enormous, and is predicted to grow exponentially as the median population age increases. Amnestic mild cognitive impairment (MCI), characterized by mild memory loss often accompanied by mild executive dysfunction, is presumed early stage AD, and the prodrome of clinical dementia. The potential to preserve, or even enhance, cognition in adults at high risk of cognitive decline due to neurodegenerative disease clearly has important implications not only for the affected individual, but also for the support system that bears the social and financial burdens of long-term caregiving. However, prior studies testing interventions to prevent or arrest cognitive decline in MCI using a variety of different pharmaceutical approaches have been largely negative. Contemporary approaches in modern health care are beginning to acknowledge lifestyle modification as a potential potent health-promoting and disease-modifying strategy to preserve or improve cognitive function and quality of life for older adults. The long prodromal period associated with MCI provides an ideal opportunity for lifestyle interventions such as increased physical activity to protect and enhance cognition when neuropathologic processes that cause decline can still be prevented or delayed.

To date, no large multi-site study has been carried out to evaluate potential therapeutic effects of aerobic exercise on brain function and markers of disease progression in adults with MCI and thus at high risk for dementia despite promising evidence from epidemiological and preliminary clinical trials. This pivotal study will examine whether moderate-to-high intensity aerobic exercise can improve cognition and other measures of brain health in adults with MCI. If successful, the results of this study may have large-scale implications for public policy regarding standard of clinical care and prescriptive practices for a fast-growing and vulnerable population of older adults. The study will also provide information about potential mechanisms through which aerobic exercise may benefit brain function by examining key markers of disease progression in brain imaging and cerebrospinal fluid (CSF). These results will likely have considerable clinical, scientific, and social significance, and may provide therapeutically relevant knowledge about the effect of aerobic exercise on AD pathophysiology.

1.1 Primary Aim

Aim 1: To test the hypothesis that 12 months of supervised moderate/high intensity aerobic exercise, relative to a stretching/balance/range of motion control, will improve cognitive function, measured using a validated composite score (ADAS-Cog-Exec) that includes select subscales (Word Recall, Delayed Word Recall, Orientation, Number Cancellation) from the Alzheimer's Disease Assessment Scale – Cognitive Subscale (version 13); tests of executive function including Trail-Making Test A and B, Digit Symbol Substitution Test, Category Fluency; and Clinical Dementia Rating (CDR) sum of box scores for Memory, Orientation and Judgement and Problem Solving in older adults with amnestic MCI.

1.2 Secondary Aims

Aim 2: To test the hypothesis that 12 months of aerobic exercise, relative to the control, will reduce clinical ratings of cognitive impairment as measured by the Clinical Dementia Rating Scale – Sum of Boxes, and total score on the ADAS-Cog13.

Aim 3: To test the hypothesis that 12 months of aerobic exercise, relative to the control, will improve executive function and episodic memory, as measured by domain-specific composite scores derived from ADAS-Cog-Exec subtests and other computer-administered tests (Cogstate, NIH Toolbox).

Aim 4: To test the hypothesis that 12 months of aerobic exercise, relative to the control, will improve daily living skills (ADCS–Activities Of Daily Living–MCI, Behavior Rating Inventory Of Executive Function–Adult Version), mood and health-related quality of life (Neuropsychiatric Inventory, 36-Item Short Form Health Survey, EuroQol 5-Item Health Questionnaire), and subjective memory concerns (Cognitive Change Index).

Aim 5: To test the hypothesis that 12 months of aerobic exercise, relative to the control, reduces the rate of regional and whole brain atrophy, and increases regional and whole brain perfusion assessed using volumetric and functional magnetic resonance imaging (fMRI).

Aim 6: To test the hypothesis that 12 months of aerobic exercise, relative to the control, will favorably alter AD biomarkers in CSF including beta-amyloid and tau, and to conduct exploratory analyses of exercise effects on other markers of brain health and disease in CSF and blood (e.g., brain-derived neurotrophic factor, vascular endothelial growth factor, measures of inflammation, proteomics/metabolomics).

Aim 7: To examine whether gender, age, baseline AD biomarker profile, apolipoprotein epsilon 4 (ApoE4) genotypes, total activity, and sleep integrity predict treatment response.

Aim 8: To examine the enduring cognitive and behavioral effects of the intervention following a 6-month extension, during which the assigned exercise is continued without supervision, to test a model for a community-based exercise prescription in adults with MCI.

Aim 9: To assess the impact of the intervention on the study partner's health, wellbeing and quality of life using the Study Partner Self-Assessment Questionnaire.

2.0 PRELIMINARY STUDIES

2.1 Summary of Clinical Findings

Although not without controversy,[1] the majority of cross-sectional and longitudinal epidemiologic studies to date,[2] including the Canadian Study on Health and Aging,[3] the Hisayama Study,[4] the Cardiovascular Health Cognitive Study,[5] the MacArthur Study,[6] and the Mayo Clinic Study,[7] indicate reduced risk of cognitive decline with increased physical activity, even among the oldest old.[8]

Exercise benefits on cognition are also supported by the results of controlled trials in normal aging. The results of a meta-analysis support an overall benefit of aerobic exercise on episodic memory, attention, processing speed, and executive function in non-demented older adults.[9] Short-term aerobic training (e.g., 4-12 months) increases whole brain and hippocampal volume, and regional gray and white matter volumes in prefrontal cortex.[10,11] Short-term aerobic exercise also augments hippocampal perfusion,[12] consistent with findings from longitudinal observational studies of lifelong fitness training.[13] Other exercise effects on brain function include favorable changes in neuronal network activity, notably in brain regions supporting higher cognitive functions. In a small controlled 4-month trial, Burdette and colleagues[12] demonstrated exercise-induced increases in cerebral blood flow and network connectivity in brain regions that support episodic memory and executive function in healthy older adults. In this study, post-intervention connectivity exceeded that observed in young adults, and the primary circuit altered by exercise was that between the hippocampus and the anterior cingulate, an important region for executive control. Positive effects of exercise on resting network activity within the frontal lobe and between frontal, posterior parietal, and temporal cortices (default mode network) are also reported by others.[11] Together, these studies demonstrate that exercise can be a potent modulator of brain structure and function in healthy older adults.

While studies of exercise in normal older adults support benefits for cognitive health, the impact of aerobic exercise on brain structure and function in adults with amnestic MCI has only recently been examined. Conceivably, exercise may not confer the same benefits when brain health is compromised by pathological processes associated with more advanced neurodegenerative disease. However, in adults with AD, improved cardiorespiratory fitness is associated with less whole brain atrophy and increased white matter volume.[14] In adults with MCI and presumably relatively less AD pathology, increasing fitness is associated with less regional brain atrophy in medial temporal (including entorhinal) cortex.[15] Although only a few controlled exercise trials in MCI have been completed to date, the results thus far provide encouraging support for cognition-enhancing effects. In the Baker et al. controlled 6-month pilot trial of supervised aerobic exercise versus stretching in MCI, they reported positive effects of exercise on cognition, particularly on tasks of executive function.[16] Favorable exercise effects on glucoregulation and Aβ levels in plasma were also reported. In a larger controlled 6-month trial of home-based aerobic exercise (walking) versus usual care in adults with MCI, Lautenschlager et al.[17] reported exercise benefits on a global test of cognitive function (ADAS-Cog), on delayed word list recall, and on a clinician-based assessment, with evidence for persisting benefits at the

18-month follow-up. These studies are encouraging, but not without limitations. In the Lautenschlager study, the interventions were not tightly controlled (usual care group was permitted to exercise), and cognitive abilities that typically improve with exercise in healthy older adults were not adequately assessed. In the Baker et al. study, sample size was small. Biomarkers in brain were not collected in either study.

In a recent larger 6-month study in MCI that included brain imaging and CSF collection,[18] moderate/high intensity aerobic exercise vs. stretching improved executive function; increased blood flow in areas of the brain that are characteristically reduced with aging and with progression of AD; and reduced CSF levels of phosphorylated tau protein – a hallmark biomarker of disease progression. The multi-site trial of exercise in adults with MCI, referred to as “EXERT,” will test whether these promising findings can be confirmed in a larger and longer duration study to definitively establish the therapeutic efficacy of moderate/high intensity aerobic exercise to slow disease progression in older adults at high risk for AD dementia.

2.2 Safety and Compliance

Aerobic exercise, at the intensity targeted in EXERT, is well-tolerated even in sedentary older adults, is the standard recommendation by the American Heart Association for older adults,19 and is commonly used in community settings to improve physical fitness. Although aerobic exercise intensity in EXERT is described as ‘moderate/high’, this descriptor in no way describes intensity of exercise completed by highly trained athletes. Moderate/high intensity of aerobic exercise in EXERT is defined as 70-80% of heart rate reserve (HRR), which is similar to 70-80% of maximum heart rate (HR) with an adjustment for baseline cardiorespiratory fitness. This exercise intensity was successfully achieved in three 6-month studies conducted by Baker et al. that enrolled over 130 older adults who were at increased risk for AD dementia. In these studies, serious adverse event (SAE) rates were low (<5%) and comparable across intervention groups.[16,18,20]

3.0 BACKGROUND AND SIGNIFICANCE

3.1 Rationale for Aerobic Exercise as a Therapeutic Intervention in Mild Cognitive Impairment

The study rationale is based on growing evidence that aerobic exercise has numerous health-restoring effects in the brain, and that a sedentary lifestyle may contribute to AD pathogenesis.[21,22] Increased physical activity improves cardiovascular health, glucose regulation, and lipid metabolism, and has positive effects on mood and stress in older adults.[23] In clinical studies, aerobic exercise is associated with improved cognitive function,[16,18] increased brain volume,[10,11] increased brain perfusion,[18] and reduced levels of AD biomarkers in CSF.[18,22]

3.2 Rationale for Aerobic Exercise Effects on AD Pathology in the Brain

The identification of biomarkers associated with AD pathogenesis can aid diagnosis, help to monitor disease progression, and identify potential underlying mechanisms. Thus, exercise-induced effects on AD biomarkers represent a key component of this trial. EXERT will examine whole brain and regional changes in structure and blood flow as measured by brain magnetic resonance imaging (MRI), and chemical biomarkers in CSF and blood. Alzheimer's Disease Neuroimaging Initiative (ADNI) studies provide valuable longitudinal MRI data in adults with MCI and early stage dementia versus healthy adults pointing to entorhinal and hippocampal atrophy as sensitive markers of disease progression.[24,25] New evidence also suggests that aerobic exercise may reverse aging- and AD-related changes in cerebral blood flow.[18] CSF concentration of beta-amyloid (Aβ) 1-42 (Aβ42), the more toxic form of the Aβ peptide, is also an important marker of AD risk.[25-28] A decrease in CSF Aβ42 is an early event preceding hippocampal atrophy, tauopathy, and other degenerative processes,[29,30] and thus a valuable index of disease progression.

Although reducing rate of progression from MCI to Alzheimer's dementia is not a primary outcome in this study, the use of progression-sensitive biomarkers will be a powerful tool to evaluate the impact of exercise on brain health, and on disease progression in future studies. In cross-sectional studies, high-levels of physical activity are associated with reduced cerebral Aβ deposition (evaluated with brain imaging using positron emission tomography) and increased CSF Aβ in older adults,[22] and in a controlled 12-month trial of moderate-intensity aerobic exercise, hippocampal volume in older adults was increased by 2%, reversing age-related loss by 1-2 years.[31] Similar aging- and AD-related markers of brain structure and function will be measured to provide important information about mechanisms that may account for positive exercise effects, including CSF levels of inflammatory markers such as IL-1β, IL-6, IL-8, TNFα,[32] about proteolytic products such as visinin-like protein-1 (a marker of neuronal injury),[33] and about growth factors such as brain-derived neurotrophic factor (BDNF).[34,35] In animal studies, exercise increases brain BDNF, reduces peripheral and brain inflammation, and slows progression of neurodegeneration.[36,37] Other new markers that probe possible mechanisms are emerging though the use of proteomics and metabolomics, which will be explored in EXERT.[38-41] Genotype effects on response to exercise will also be examined, including the ε4 allelic variant of the apolipoprotein E (ApoE) gene that has independent and synergistic effects on Aβ-related AD pathogenesis, atherosclerotic changes, cardiovascular disease, and cognitive decline.[42-48]

3.3 Rationale Supporting Selection of Primary and Secondary Cognitive Outcomes

Recent findings from ADNI indicate that ADAS-Cog scores reliably change over 12 months in MCI. A modified version of the ADAS-Cog (version 13) supplemented with other tests, referred to as ADAS-Cog-Exec, has been validated[49] and will be used as the primary outcome measure to assess efficacy in EXERT. This composite includes ADAS-Cog13 subtests shown to be maximally sensitive to change over time in MCI (Word Recall, Delayed Word Recall, Orientation, Number Cancellation), additional measures of executive function (Trail-Making

Test, Digit Symbol Substitution Test [DSST], Category Fluency), and Clinical Dementia Rating scale sum of box scores for Memory, Orientation, and Judgement and Problem Solving. Although inclusion of supplemental measures may increase variability overall, a heavier executive function load in the ADAS-Cog composite will likely add sensitivity given prior evidence of beneficial exercise effects on this cognitive domain. The selected secondary cognitive measures were either sensitive to aerobic exercise in previous studies of healthy older adults[20,50,51] or individuals with MCI,[16,18,20] or to early hippocampal/medial temporal cortical changes in MCI.[52]

3.4 Rationale for Length and Design of EXERT

In preliminary studies, beneficial cognitive effects in MCI were observed following 6 months of moderate/high intensity aerobic exercise. EXERT includes an extended intervention period of one year for the primary analyses, which should only increase power to detect a treatment effect on cognition and biomarker endpoints in MCI. A 6-month unsupervised extension of the intervention will provide additional information regarding longer-term efficacy and exercise adherence that will provide important translational value for future community-based and, potentially, therapeutic programs for adults with MCI.

3.5 Rationale for Population Selection

Adults with a cognitive profile indicating amnestic MCI will be enrolled in EXERT to examine potential therapeutic effects on brain structure and function in early stage AD when neuropathologic processes that cause decline can still be potentially prevented or delayed. Although enrollment will not be restricted to biomarker-positive patients, the trial will collect CSF biomarkers and MRI measures of atrophy and blood flow that can be examined relative to treatment response and group assignment in secondary analyses.

3.6 Rationale for Selected Exercise Dose

The selected dose of aerobic exercise was based on previous work showing that the targeted frequency, duration, and intensity improved cognitive function both in healthy older adults[10,11,31,51] and in MCI.[16,18] Variability in achieved adherence for the aerobic training (AX) group will permit examination of relationships between exercise volume (based on intensity, duration, frequency) and magnitude of cognitive and biomarker response. A maximum of 35% HRR is targeted in the stretching/balance/range of motion (SBR) group as this level of exertion falls well below American College of Sports Medicine (ACSM) guidelines for moderate-intensity exercise[19] and thus ensures maximal separation in exercise dose between the groups.

3.7 Rationale for Intervention Implementation in Community-Based Facilities

One aim of EXERT is to test the feasibility of prescriptive aerobic exercise for adults with MCI using community-based facilities equipped to successfully provide adequate resources and a structured system of social and safety support. The national office of the YMCA (referred to as

Y-USA) and local YMCA branches will provide facilities for exercise and trainer supervision of participants as they complete their assigned intervention activities. The Y-USA has worked closely with the study leadership to identify suitable YMCA facilities near participating clinic sites that will provide oversight of study-related activities carried out at the YMCAs, and ensure participant safety and high levels of intervention fidelity. This innovative collaboration between academia and a well-established national nonprofit community-based organization will test a highly translatable mechanism for delivery of a promising behavioral intervention to slow or prevent progression to Alzheimer's dementia.

3.8 Rationale for Magnetic Resonance Imaging

Longitudinal data collected in ADNI provides robust evidence that structural MRI can be a credible predictor of future cognitive and functional decline, and is sensitive to total and hippocampal/entorhinal volume reductions over a 12-month period.[24,25,53] Jack et al. examined the role of structural MRI outcomes as endpoints for disease progression in therapeutic trials for AD and reported that multi-site consistency is feasible.[54] Others highlight particular vulnerability of the nearby entorhinal cortex to atrophy over the same period of time.[55] The results of prior normal aging studies show positive effects of exercise on hippocampal, prefrontal, and posterior parietal regions;[10,11,31] and those of a recent study in MCI indicate increased brain perfusion using fMRI in prefrontal and posterior parietal regions.[18] EXERT will test whether 12 months of supervised aerobic exercise can attenuate disease-related atrophy and increase perfusion in hippocampal, prefrontal, and posterior parietal regions that support memory and executive function.

3.9 Rationale for Inclusion of Actigraphy

Actigraphy will be used to collect multiple 24-hour samples of physical activity to test the impact of total activity and of sleep quality on treatment response.

Aerobic exercise has favorable effects on multiple neurobiological systems, one of which controls sleep. Results from the Baltimore Longitudinal Study of Aging indicate that poor sleep may have a neuropathologic signature: in community-dwelling older adults, brain Aβ burden was increased in older adults with self-reported shorter sleep duration and quality,[56] which may point to an important role of sleep in Aβ clearance.[57,58] Behaviorally, when sleep is disturbed, memory is impaired.[59,60] In healthy adults, 6 months of aerobic exercise has been shown to significantly improve overall sleep quality,[61-63] with downstream benefits on inflammatory processes.[63] EXERT will expand these findings to assess intervention effects on sleep quality in adults with MCI.

While regular participation in structured exercise programs may influence quality of nighttime activity, short intense bouts of exercise may also impact total quantity of other physical activity completed during other times of the day. Controlled trials of structured exercise typically quantify activities that occur only during these short bouts. The importance of objective measures of total physical activity is underscored by the results of prospective studies showing that increased

total physical activity predicts reduced dementia risk,[64] and that older adults who engage in structured exercise may actually be less active at other times of the day.[65] Controlled exercise trials that fail to quantify total physical activity may overlook an important mediator of response to the intervention.

3.10 Rationale for Collection of Biofluids

CSF biomarkers associated with AD pathology such as Aβ42, Aβ 1-40 (Aβ40) and total and phosphorylated tau protein will be measured, as will relevant biomarkers in blood. Residual specimens will be banked in the ADCS biospecimen bank to permit putative and evolving biomarkers associated with AD pathology (such as brain-derived neurotrophic factor [BDNF], vascular endothelial growth factor [VEGF], insulin, inflammatory markers) to be examined later.[66,67]
Although some studies suggest that the greatest cognitive benefit may be seen in adults with the ε4 allelic variant of the ApoE genotype,[68] recent data indicate that exercise may benefit ε4 carriers and non-carriers alike. Specific Aim 7 will examine whether ApoE4 carriage is a predictor of treatment response.

4.0 POTENTIAL RISKS AND BENEFITS ASSOCIATED WITH INTERVENTION

4.1 Potential Benefits

There is an urgent need to identify promising treatments for adults with MCI who are at increased risk for progression to Alzheimer's dementia. At present, no large multi-site study has evaluated the potential therapeutic effect of 12 months of aerobic exercise in MCI. There are significant potential scientific and clinical benefits for the subject population. Although regular exercise is the standard of care to maintain optimum physical health and prevent various medical conditions such as cardiovascular disease and diabetes, exercise is not prescribed to maintain optimum cognitive health or prevent diseases associated with cognitive decline. In addition, little is known about the effects of aerobic exercise on AD pathophysiology with possible consequences for Aβ regulation and tau phosphorylation, and for structural brain integrity characteristically compromised by disease progression. EXERT has the potential to identify important mechanisms related to exercise effects on cognition in the earliest stages of AD. Participants will receive a free 18-month membership to a participating YMCA, and a personal trainer who will oversee their exercise in the first 12 months of the study. The relatively minor risks posed by the intervention, cognitive testing, MRI and lumbar puncture (LP) are outweighed by the value of the scientific investigations outlined in this proposal that could potentially alter widespread practices for standard of care in MCI.

4.2 Potential Risks

Although regular physical activity and exercise have numerous health benefits on blood circulation, metabolism, lipoprotein profile, bone mass, risk of falling and disease,[69] high-intensity exercise can be associated with greater risk of adverse cardiovascular events,[70] as

well as orthopedic[71] and soft tissue (muscle, tendon, cartilage) injury. High-intensity exercise is also associated with lower adherence to a training regimen than lower-intensity exercise.[72] Evidence favoring high-intensity over moderate-intensity exercise, however, is provided by a recent joint report by the ACSM and the American Heart Association[73] showing greater risk reduction for cardiovascular disease and all-cause mortality. The EXERT protocols of moderate/high intensity aerobic training are consistent with the latest ACSM exercise recommendations for older adults.[19] Risk of injury is strongest for overweight and unfit older adults.[74] When sedentary adults begin to walk or jog, they may develop foot, leg, and knee injuries when training is performed more than 3 days per week and for more than 30 minutes per session;[75] while prolonged exercise may result in overuse-related orthopedic injuries.[74] The EXERT protocol that includes gradual increments of exercise intensity and duration, under the supervision of a trainer and taking into account individual needs and limitations, is in accordance with this recommendation and its safety has been established in other community-based exercise programs.[76-78] In previous pilot exercise studies in MCI on which the protocol is based, of the 100 enrollees who completed the high-intensity arm (meeting all EXERT inclusion criteria),[16,20,79] only 3 subjects (3%) were dropped due to a cardiovascular adverse event (AE) (follow-up tests indicated chronic disease pre-dating study entry for all 3 adults).

There are no known risks associated with participation in the SBR control group.

<u>400 m Walk Test</u>. Participants will be screened for cardiac or pulmonary disorders to exclude those who cannot safely complete this test. Participants will be instructed to walk as quickly as possible to complete 10 laps, totaling 400 m. Safety precautions will be taken while administering this test by monitoring physical signs and symptoms of fatigue or discomfort, and by applying standardized stopping criteria. If the participant reports chest pain, tightness or pressure, significant shortness of breath or difficulty breathing, or feeling lightheaded or faint, the test will be terminated. The 400 m Walk Test will be performed in settings where a defibrillator is available. Clinic staff members are trained to provide immediate care and promptly contact medical personnel in the event of a medical emergency. Also, institutional and community Emergency Medical Services (EMS) will be engaged (call 9-1-1) if needed.

<u>Brain MRI</u>. All participants will be screened for presence of iron-containing substances near the head that can be perturbed by the magnet of the MRI scanner, including pacemakers, cardiac defibrillators, or other metal fragments in or near the head. There is a remote risk of external injury to the body when metal outside of the magnet is brought close to the scanner. However, scanners are located in secure rooms to avoid inadvertent introduction of metals into the magnetic field. Other than injuries from unsecured metal materials, there are no known longstanding detrimental health effects associated with exposure to high magnetic fields, magnetic gradients, or radiofrequency energy used in MRI. Because the procedure requires participants to lie still inside a narrow tube, participants may experience claustrophobia or mild body position discomfort.

<u>Lumbar Puncture</u>. The risk for serious AE related to LP (e.g., post-LP headache, infection, nerve root damage) is very low.[80] LP may be associated with pain during the procedure. This is

usually temporary and confined to the lower back. Headache may occur in about 5% of elderly adults who receive an LP using the ADCS LP protocol. Less commonly, in about 1-4% of subjects, a persistent low-pressure headache may develop, probably due to leakage of CSF. On rare occasions, a blood patch (injection of some of the subject's blood to patch the CSF leak) may be needed. Potential but rare risks of LP include infection, damage to nerves in the back, bleeding into the CSF space, and death. Risk for these events is less than 1%.

General. Blood collection may be associated with transient discomfort and bruising, and a small risk of infection. Cognitive testing may produce frustration in some subjects. Questionnaires about mood may uncover or potentiate feelings of dysthymia and helplessness.

5.0 SAMPLE SIZE AND STATISTICAL PLAN

This study is a multisite, randomized, single-blind trial comparing the effects of supervised moderate/high intensity aerobic exercise (70-80% HRR) versus stretching/balance/range of motion (≤35% HRR) in adults with MCI (N=300).

5.1 Randomization

In this single-blind study design, eligible participants per site will be randomized using a simple 1:1 schedule to either the AX or SBR group, and stratified by site, ApoE4 carrier status (yes or no), and gender. After 12 months of supervised exercise, all participants will transition to independent exercise (without supervision) and will be instructed to continue their assigned physical activity regimen for an additional 6 months to test the efficacy of a translational model.

5.2 Power and Sample Size Determination

Power calculations for EXERT were based on two-sample t-tests.[81] Sample sizes were estimated using 12-month ADAS-Cog change scores from the ADCS MCI trial, targeting power =80%, 2-sided alpha =5%, and SD =4.1. Calculations ranged over effect sizes in ADAS-Cog from 1.0–2.0, and dropout rates from between 10-25%, which are similar to other reports, including multi-center trials of physical activity interventions in older cohorts.[11,16,17,31,82,83] The trial is powered to conservatively accommodate 20% attrition. Based on these considerations, and an effect size =1.5, 291 total subjects are required. The trial will target enrollment of 300 participants. The statistical approach that includes analyses of a composite measure rather than an individual test score is expected to increase statistical power. In light of evidence showing exercise benefits on executive function in other studies, and sensitivity of select CDR and ADAS-Cog13 subscales to more subtle changes associated with MCI, use of the ADAS-Cog-Exec composite, in particular, should also increase sensitivity and power.

To estimate an intervention effect on biomarkers, the difference in hippocampal atrophy between ADNI MCI participants versus healthy controls (HCs) without the ApoE4 allele was used. With a total sample size of 300, assuming 20% attrition and SD =1.54 (based on ADNI data), power to detect a difference in percent change from Baseline to Month 12 in hippocampal

volume between intervention groups as small as 0.56%, is 80% (5% alpha). As a reference, the difference in percent change from Baseline to Month 12 in hippocampal volume between ADNI MCI and normal controls ApoE4 non-carriers (presumed to be free of disease) is estimated to be 1.02%. Thus, the study is powered to detect an improvement equivalent to 0.559/1.02 =54.8% of this ApoE4 related effect. With regard to entorhinal cortical thinning, assuming SD =1.68 (ADNI data), power to detect a difference in percent change from Baseline to Month 12 as small as 0.61% between intervention groups is estimated at 80%. Again as a reference, the difference in percent change from Baseline to Month 12 between ADNI MCI and normal controls ApoE4 non-carriers is estimated to be 1.49%. Thus the study is powered to detect an improvement equivalent to 0.61/1.49 =40.9% of this ApoE4 related effect.

The preceding calculation assumes a maximal intervention effect would result in normalization of atrophy to the rate seen in healthy controls. However, Erickson et al.[31] report hippocampal *expansion* with exercise in non-demented older adults. Here, 120 subjects completed a 12-month program of aerobic exercise (mean age+/-SD: 67.6yrs+/-5.81yrs) or stretching (65.5yrs+/-5.44yrs), and were tested at baseline, and at months 6 and 12. The aerobic group had significant *increases* in left and right hippocampal volumes (2.12% and 1.97% change from baseline, respectively) after 12 months. For the stretching group, left and right hippocampal volumes *decreased* (-1.40% and -1.43% change, respectively) after 12 months (all p's<0.001). Assuming an effect as large as that observed in Erickson et al. (~2%) and ADNI estimated standard deviation of change (SD =1.7), 20% attrition, and 2-sided alpha =5%, 300 randomized participants should provide greater than 99% power. Despite the compelling data described by Erickson et al., EXERT is conservatively powered to detect a relatively modest normalization of hippocampal atrophy.

5.3 Selection of Participants to be Used in Analysis

The modified intent to treat (mITT) population will be used for primary analysis. The mITT population will include all eligible individuals who, (1) began the exercise intervention and (2) completed at least one post-baseline assessment for the primary analysis. Exploratory analyses will include a per-protocol population of all eligible participants who, (1) completed the 12-month assessments for the primary analysis, (2) participated in at least 60% of the prescribed exercise sessions, and (3) met exercise goals in at least 60% of the supervised sessions, and an intent-to-treat (ITT) population of all eligible individuals who are randomized.

5.4 Efficacy Analysis

5.4.1 Analysis of Primary Outcome

To address **Aim 1** for the primary efficacy analysis, longitudinal scores from baseline through the first 12 months will be analyzed using a mixed effects model for repeated measures (MMRM) to assess differences in scores between intervention groups. The MMRM model will include terms for time, intervention, and baseline ADAS-Cog-Exec, intervention-by-time

interaction, baseline ADAS-Cog-Exec by time interaction, plus covariates that include site, ApoE4 carrier status, sex, and additional covariates meeting the criteria below.

Time will be treated as categorical. An analysis of covariance model imputing missing data with multiple imputation will be used as a sensitivity analysis. As a second sensitivity analysis, a similar MMRM model will be fit with time modeled as a continuous factor. If the longitudinal patterns over time are markedly non-linear, such as might occur due to learning effects, a quadratic term for time will be included in the model. In this case the intervention-by-time linear term assesses whether there is a relative difference between intervention groups that grows linearly with time. Potential confounders (baseline Walk Test time, age, education, 3MSE baseline score, and use of cholinesterase inhibitors) will be included in efficacy analyses as covariates if the following two conditions are satisfied: (1) imbalance at baseline ($p<0.1$) and, (2) association between the covariate and the response ($p<0.15$). Safety data will be analyzed using exact contingency table methods by intervention group assignment.

Differences in the primary outcome of change in ADAS-Cog-Exec between intervention groups will be tested at the two-sided 5% significance level. No adjustment to the type 1 error level will be made for secondary analyses.

5.4.2 Analyses of Secondary Outcomes

Aims 2–4: Similar analyses to that described for the primary analysis will be performed on the Clinical Dementia Rating Scale – Sum of Boxes (CDR-SB), ADAS-Cog13 total score, Executive Function and Episodic Memory Composites, tests of daily living skills (ADCS–Activities Of Daily Living–MCI [ADCS-ADL-MCI], Behavior Rating Inventory Of Executive Function–Adult Version [BRIEF-A]), mood and health-related quality of life (Neuropsychiatric Inventory [NPI], 36-Item Short-Form Health Survey [SF-36]), EuroQol 5-Item Health Questionnaire [EQ5D]), and subjective memory concerns (Cognitive Change Index).

Aim 5: Change in brain volume and inter-regional correlations in brain functional activity will be compared between treatment groups. The analysis will be completed separately for each volumetric and functional MRI parameter.

Aim 6: CSF biomarkers of brain health and disease will be examined using a similar analytic strategy to that described for the analysis of the primary outcome using MMRM and other sensitivity models to test for intervention effects. Covariate adjustment, sensitivity analyses, and multiple imputation analyses will be carried out as specified under Aim 1.

Aim 7: To examine whether sociodemographic factors, genotype, actigraphy outcomes, or baseline cognitive and biomarker profiles predict treatment response, MMRM and sensitivity models as described above will be performed using subgroup analyses. Model fitting and covariate adjustment will be carried out as specified under Aim 1.

Aim 8: To examine the enduring cognitive and behavioral effects of the intervention following the 6-month unsupervised exercise extension, MMRM and sensitivity models as described above will be performed. Model fitting and covariate adjustment will be carried out as specified under Aim 1.

Aim 9: To examine the impacts of the intervention on the study partner, MMRM and sensitivity models as described above will be performed on data obtained using the Study Partner Self-Assessment Questionnaire.

5.5 Safety Analysis

Safety will be assessed by summarizing and analyzing AEs during the intervention period. Adverse events will be coded according to established Medical Dictionary for Regulatory Activities (MedDRA) terms and summarized by MedDRA System Organ Class and Preferred Term. Treatment-emergent adverse events (TEAE) will be defined as events that first occurred or worsened on or after randomization.

An overview of AEs, including the number and percentage of participants who died, suffered SAEs, discontinued due to AEs, and who suffered TEAEs, will be provided. A comparison between intervention arms will be performed. Summaries of AEs by within system organ class will be provided for: (1) pre-existing conditions, (2) TEAEs, and (3) SAEs. Discontinuations due to AEs will also be listed.

5.6 Termination of the Trial

No criteria for trial termination are specified. If safety or efficacy issues are identified during trial conduct, the Data and Safety Monitoring Board (DSMB) may decide to stop the study.

6.0 INTERVENTIONS AND IMPLEMENTATION

6.1 Overview

Participants will complete EXERT interventions at participating YMCAs located near the selected clinic sites across the U.S. The YMCA will provide 18-month memberships at no cost to participants. In the first 12 months, a study-certified YMCA Trainer will supervise all participants for the first 8 exercise sessions completed (weeks 1 and 2), and for 2 of 4 weekly sessions thereafter through Month 12. At this time, participants will transition to independent exercise and continue their assigned exercise programs for the final 6 months of the study without supervision. To encourage adherence and optimize cost efficiency, Trainers will provide supervision to small groups of participants (2-4 individuals) randomized to the same intervention whenever possible. Compliance will be evaluated using multiple mechanisms including HR monitoring, participant ratings of perceived exertion, entries in participants' Physical Activity Logs, Trainer assessment of effort, and weekly data review by the YMCA-Project Manager (Y-

PM) and the Intervention Oversight Team (includes the PDs, Wake Forest team of exercise trial specialists, and Y-USA). These mechanisms will provide multiple and regular opportunities to discuss participant progress, identify and resolve barriers, and encourage high levels of adherence to study protocols. The Intervention Oversight Team has the necessary expertise to successfully accomplish this objective in EXERT.

6.2 Collaboration with the YMCA

EXERT has partnered with the national office of the YMCA (referred to as ‘Y-USA’) to provide exercise facilities and trained personnel to supervise participants as they complete their assigned intervention protocols. The YMCA Trainers maintain certifications to keep current with latest knowledge about fitness and its practice, and have experience supervising older adult exercise. This collaboration with the Y-USA will move the field forward in bridging the rift between academic research and health care delivery, thus providing additional important translational value to the study.

Study-certification of Trainers will involve completion of training regarding the study protocol, proper implementation of the interventions, data capture of adherence outcomes, and AE recording and reporting.

Intervention adherence data will be recorded by Trainers on Case Report Forms (CRFs) during supervised exercise sessions and will include type of exercise completed, HR during exercise, ratings of perceived exertion, and exercise duration and intensity. During supervised sessions, Trainers will also review participants’ Physical Activity Logs to ensure they are properly completed and study goals are met, and complete an AE Checklist that will be faxed to the clinic for review and follow-up as per study protocol (see **Sections 14 and 15**). The Trainers and Y-PMs will provide regular updates as requested by the clinic, the Intervention Oversight Team, and the ADCS. All Y-PMs will attend a telephone conference twice per month, attended by Clinic YMCA Liaisons, the Intervention Oversight Team, and the ADCS to ensure efficient coordination and implementation of the intervention protocols. Trainers will also attend at least one telephone conference call per month with members of the Intervention Oversight Team.

6.2.1 YMCA Study Team

The YMCA team will consist of the following:

Y-USA. The Senior Manager of Program Development for Y-USA, and the Y-USA Study Manager will serve as the primary interface between the national office of the YMCA, the PDs, the Wake Forest team, the ADCS, the participating YMCA Associations, and the Y-PMs.

YMCA Project Manager (Y-PM). The site Y-PM, a senior level staff member will oversee Trainer supervision of EXERT participants, review and upload all adherence data to the central data repository, respond to adherence data queries as needed, and maintain a close bidirectional relationship with the clinic to facilitate efficient monitoring of participant progress and

management of study-related issues. The site Y-PM will report all AEs to the clinic, maintain regular communication with the Clinic YMCA Liaison regarding participant progress, and participate in bi-weekly conference calls with other site Y-PMs and Clinic YMCA Liaisons, the Intervention Oversight Team, and the ADCS to review study progress and address challenges as they arise.

YMCA Trainers. Trainers at each site will supervise participants, providing encouragement and exercise-related expertise to help participants achieve the targeted intervention goals and to maintain high levels of adherence throughout the trial. During supervised sessions, Trainers will record adherence data on CRFs, review participant Physical Activity Logs to identify and address challenges and assess adherence, complete the AE Checklist, maintain close communication with the Y-PM and the Clinic YMCA Liaison regarding participant progress and, as schedule permits, participate in twice per month conference calls to review study progress with other Trainers, Y-PMs, Clinic YMCA Liaisons, the Intervention Oversight Team, and the ADCS.

6.2.2 Interface Between the Clinic and the YMCA

The clinic will be responsible for recruitment and screening of study candidates, all assessments of primary and secondary outcomes, randomization, and AE tracking, management and reporting to the site Institutional Review Board (IRB), the Intervention Oversight Team, and the ADCS.

Once randomized, the site Y-PM will be notified by the clinic and the participant's contact information will be provided so that the first orientation meeting at the YMCA can be scheduled. The sharing of contact information with the YMCA will be covered in the consent form. The Y-PM will review all adherence CRFs completed by the Trainers during supervised exercise sessions, and will enter and upload all compliance data to the ADCS Electronic Data Capture (EDC) and respond to any associated data queries.

6.2.3 Adverse Event Reporting by YMCA Trainers

The AE Checklist will be completed weekly during a Trainer-supervised exercise session. This survey will query change in health or physical status in the last week, and will include questions to identify potential SAEs (death, life-threatening, hospitalization, disability or permanent damage). The AE Checklist will also query TEAEs, which most commonly include muscle or joint aches including flare-ups of pre-existing musculoskeletal conditions (e.g., arthritis, foot bone spurs or neuromas). The AE Checklist will be faxed to the clinic to permit review by the Study Clinician within 24 hours of AE notification by the participant.

Response to emergent serious or life-threatening medical events that occur while the participant is exercising at the YMCA will follow standard YMCA protocols that apply to the management of these events for any member of the facility. When appropriate, using the best judgment of YMCA staff, the EMS may be engaged to stabilize and transport an individual to a nearby

hospital for treatment. Other standard YMCA protocols may be used in the event of an onsite injury or medical event.

6.3 Implementation of the Interventions

Both intervention groups will complete their assigned exercise at participating YMCA branches near the selected clinics. Intensity and duration of exercise in both groups will be gradually increased over the first 6 weeks in the study, and then maintained at the intervention-specific targeted level (described in **Sections 6.3.1** and **6.3.2**) for the remainder of the 18-month intervention.

All participants will be provided with a digital HR monitor that includes a chest strap and a wrist-worn monitoring device and watch, and taught to use this device to monitor and record HR while exercising. Participants taking medications with HR-lowering effects will be provided with HR monitors, but will be taught to rely more heavily on other measures of effort intensity such as breathing rate, and ability to talk while exercising. During supervised sessions, effort will also be assessed by the Trainers. Participants in both intervention groups will be trained to properly use the Rating of Perceived Exertion (RPE) modified Borg scale (ratings range from 1=very mild, to 10=very, very hard) in the first 2 weeks of the program as a supplemental means of assessing exercise effort. Proper use of the RPE scale will be assessed and retrained by the Trainer at regular intervals and as needed throughout the study.

During supervised sessions for both intervention groups, the Trainer will record HR and RPE every 5-10 minutes, as well as other relevant exercise parameters (e.g., total duration, type of exercise completed, treadmill speed and grade). The Trainer will also record any additional observations that might assist the Intervention Oversight Team in their weekly review of participant progress. These data will be uploaded to the EDC by the Y-PM within 48 hours of the exercise session.

For all supervised and unsupervised exercise sessions completed, participants will record the following in their Physical Activity Log: date and time, type of exercise, exercise duration, average HR, maximum RPE achieved, and other intervention-specific details (e.g., body regions stretched, grade/resistance and speed of equipment used). During each supervised session, the Trainer will review these entries, discuss and problem-solve issues that are uncovered during this review, and confirm the exercise schedule for the next few days. At the Baseline, Month 6, and Month 12 clinic assessments, participants will receive a 6-month supply of Physical Activity Logs. At the end of each 4-week period, the Trainer will collect completed Logs and store them in a locked cabinet until retrieved by the Clinic YMCA Liaison, which will occur monthly. In the final 6 months of the study (unsupervised), participants will return the last set of 6 completed Physical Activity Logs to the clinic at the Month 18 visit.

Progression through the 18-month intervention will involve transition from one ‘phase’ to another. This transition will provide an objective measure of progress for participants, and a structure that will facilitate Trainer, Y-PM, and Intervention Oversight Team efforts to anticipate

and address participant issues that may characteristically change as they advance through the study (e.g., development of mastery, boredom with routines). The phases will also differ with regard to study expectations of participants and emphasis in training, which will shift from the basics of the prescribed exercise routines in the early part of the study to strategies to support and maintain independent exercise later in the program. The phases are defined by months in study, where 'month' always refers to exactly 4 weeks.

- In **Phase 1** (months 0-3), participants will receive instruction about the program and any equipment to be used, about proper form and technique, attire, exercise facility etiquette and symptoms of fatigue and injury, and exercise expectations will be gradually increased with the goal of reaching intervention-specific activity goals by the end of week 6 (i.e., 70-80% HRR for the AX group; ≤35% HRR for the SBR group). Training will also focus on participants' self-efficacy to begin to develop a strong foundation that will support adherence to the interventions over long periods of time. Participants will be asked to limit travel as much as possible, and will not be permitted to engage in alternate exercise activities (classes, outside walking). For all participants, the first 8 sessions of exercise will be supervised by a study-certified YMCA Trainer.

- In **Phase 2** (months 4-6), the goal will be for participants to begin to develop mastery of the intervention-specific activities. In Phase 2, participants will be allowed to engage in 2 alternate activities per month (e.g., aerobic or easy yoga classes). Alternate activities must receive prior approval by the Trainer and the Y-PM to ensure that the activity is consistent with participant-specific intervention goals. Continued participation in 2 alternate activities per month in Phase 2 will also be subject to approval by the Intervention Oversight Team who will regularly review the participant's adherence record. The opportunity for participants to engage in alternate activities serves as a reward and thus an adherence-promoting strategy.

- In **Phase 3** (months 7-9), participants' target HR will be re-calculated using the most recent resting HR measurement obtained during the Month 6 clinic assessment. This reassessment will ensure that the intervention-specific HR training goals are appropriately adjusted for current fitness levels (using surrogate measure of resting HR). In Phase 3, participants will continue their prescribed activities using the same supervision schedule, and will have the opportunity to engage in 1 pre-approved alternate activity per week (e.g., aerobic or easy yoga class). As in Phase 2, continued participation in alternate activities will be subject to approval by the Intervention Oversight Team who will regularly review participant adherence.

- In **Phase 4** (months 10-12), participants will adhere to the same exercise regimen (4 times per week) and supervision schedule (twice per week), and will continue to have the opportunity to participate in 1 pre-approved alternate activity per week, subject to approval by the Intervention Oversight Team. The supervised sessions will include training and discussions to prepare participants for independent exercise.

- In **Phase 5** (months 13-18), participants will transition from supervised to unsupervised exercise following the Month 12 clinic assessment. During this time, participants will continue to complete their assigned exercise interventions as prescribed but without supervision other than what is normally provided by YMCA staff to all YMCA members. Attendance will be tracked using the YMCA membership card electronic scan records and participant-recorded entries in Physical Activity Logs.

6.3.1 Aerobic Training Intervention

The aerobic exercise (AX) will consist of treadmill walking supplemented with use of the elliptical trainer or stationary cycle at moderate/high to high levels of cardiorespiratory intensity, defined as 70-80% of HRR (HRR =220-age-resting HR) for 30 min, 4 days per week. Each session will include an additional 10 minutes of warm-up and an additional 5 min for cool-down. In study Phases 2-5, participants will be permitted to attend pre-approved aerobic exercise classes at the YMCA to encourage continued participation over the duration of the 18-month trial. High levels of retention (>80%) and adherence (>90%) have been achieved in previous exercise trials in healthy and memory-impaired older adults.[16,20,82,84,85]

The following algorithm – previously used in studies of older, sedentary adults[16,20,79] – will be used to establish AX HR targets and exercise durations during the initial ramp-up period and for the remainder of the 18-month program:

Exercise Goals	Week	Warm-Up, Cool-Down Time	RPE	Effort, Time in Training HR Zone
**Target HR = 50-60% HRR; Total Duration = 15-25 minutes**	1	3 min, 2 min	1 – 2	50%, 10 min
	2	5 min, 5 min	3 – 4	60%, 15 min
**Target HR = 65-70% HRR; Total Duration = 30-45 minutes**	3	5 min, 5 min	4 – 5	65%, 20 min
	4	5 min, 5 min	5 – 6	65%, 25 min
	5	5 min, 5 min	5 – 7	65%, 30 min
	6	10 min, 5 min	6 – 8	70%, 30 min
**Target HR = 70-80% HRR; Total Duration = 45 minutes**	7+	10 min, 5 min	6 – 8	75%, 30 min

Although the AX algorithm will identify appropriate training HR targets for the majority of older adults, the targets may require slight adjustments by the Trainers to meet specific needs of participants. Adjustments might include increasing duration of the ramp-up period beyond 6 weeks if participants have difficulty meeting the exercise intensity and duration goals due to physical limitations (e.g., musculoskeletal issues, obesity), or lowering of training HR targets for a period of time to permit participants to adjust to the program or recover from injury or unanticipated prolonged inactivity. Adjustments to the AX algorithm will be regularly reviewed by the Intervention Oversight Team to ensure that study goals are met.

6.3.2 Stretching, Balance, and Range of Motion Intervention

The stretching, balance, and range of motion (SBR) intervention will include a rotating and varied routine of stretching exercises for large and small muscle groups, and activities to improve balance and range of motion. SBR activities will be tailored so that participants are able to maintain HR at or below 35% HRR during each session, which will be completed 4 days per week for 45 min per session.

The SBR algorithm described below will be used to establish HR targets and exercise duration during the ramp-up period and for the remainder of the 18-month program. The algorithm serves as a guideline and can be adjusted by the Trainer when study goals are not met. For example, adjustments to the SBR regimen may be required if achieved HR consistently exceeds the maximum allowable target. If HR continues to rise even under very low exercise demands (e.g., cardiac response to gentle stretches may be greater for obese or severely deconditioned adults), new maximum HR targets may be set, subject to revision as participants become accustomed to physical activity. Adjustments to the SBR algorithm will be regularly reviewed by the Intervention Oversight Team to ensure that study goals are met.

Exercise Goals	Week	Warm-Up, Cool-Down Time	RPE	Total Exercise Duration
Target HR ≤ 35% HRR	1	3 min, 2 min	1 – 2	15 min
	2	5 min, 5 min	1 – 3	20 min
	3	5 min, 5 min	1 – 3	25 min
	4	5 min, 5 min	1 – 3	30 min
	5	5 min, 5 min	1 – 3	35 min
	6	5 min, 5 min	1 – 3	40 min
	7+	5 min, 5 min	1 – 3	45 min

6.4 Adherence

Adherence will be assessed using several strategies, including Physical Activity Log entries, and adherence outcomes obtained during the supervised sessions that include attendance, type of activity completed, exercise duration, time in target HR zone, subjective ratings of perceived effort, and achieved HR.

The Intervention Oversight Team has extensive experience implementing behavioral management strategies to support a positive exercise environment. The goal in EXERT will be for participants to complete at least 80% of the prescribed exercise sessions. Participants who miss a supervised exercise session will be contacted by the Trainer to confirm that the participant will attend the next scheduled supervised session. Participants who miss 2 consecutive supervised sessions for any reason will be contacted by the Trainer or Y-PM to problem-solve issues that obstruct attendance, provide encouragement, and schedule the next supervised session. For these cases, the Intervention Oversight Team may contact the Y-PM and Clinic YMCA Liaison to discuss strategies to increase adherence. Participants who do not

attend at least 10 supervised or unsupervised sessions per month will be counseled to improve attendance, and motivational strategies to increase adherence and limit obstacles to study participation will be implemented if necessary (e.g., goal setting, reinforcers identified and scheduled, etc.). In the first 12 months of the intervention, participants without an excused leave of absence who miss 48 exercise sessions within a 3-month period will be dropped from the intervention due to non-compliance.

If a participant misses a supervised exercise session for reasons that are unplanned (excludes missed visit for holiday closure, weather, and other anticipated reasons), a Missed Visit CRF will be completed by the Trainer and uploaded to the EDC by the Y-PM to permit tracking of these events.

For planned travel, total time away must be no more than a total of 2 months over the course of the study, and no more than 1 month at any one time to be considered for study entry. Participants must be willing to continue the assigned exercise unsupervised if travelling out of the area for more than 1 week. For all planned absences, the Trainer will meet with the participant in advance to develop an acceptable program for continued exercise.

Although high levels of adherence will be stressed at all times, adherence will be particularly important in the 4 weeks preceding the Month 6 and Month 12 assessment clinic visits. At the Intervention Oversight Team's discretion and in consultation with the site Principal Investigator (PI), the acceptable window for completion of these visits may be expanded by up to 6 weeks if this shift would permit participants to resume exercise at the targeted dose for at least 3 weeks following a temporary discontinuation and prior to assessment of primary study outcomes. Assessment window expansions will not impact the overall duration of the 18-month trial.

6.5 Safety During Exercise

While completing the intervention, participants will be instructed as to best-practice exercise guidelines including warm-up and cool-down activities, positioning and shifting of body weight, proper form, safety around exercise equipment, exercise quantity (intensity, duration frequency) and attire to minimize risk of discomfort and injury. These discussions between the Trainer and participant will be initiated on Day 1 of the intervention, and will continue throughout the 12 months of supervised exercise. Physical activities will be gradually increased in intensity and duration in the initial stages of the intervention period to reduce likelihood of injury and to promote self-efficacy regarding completion of the assigned activities. Regular oversight by Trainers will provide numerous opportunities for encouragement, minor adjustments to the routine or program as needed to meet study goals and to address participant challenges, education about the importance of exercise and adherence to study protocols, and early identification of practices that could ultimately lead to injury if not corrected. Frequent contact between the participant and the Trainer also affords numerous opportunities to query AEs, opportunities that would not otherwise be available for participants in clinical trials that include minimal staff contact between assessment visits.

During supervised sessions, the Trainer will monitor HR, breathing rate, and other signs of physical effort. Sessions will be terminated and clinic staff immediately notified if any of the following symptoms become apparent:

- Resting HR is <40 beats per minute (bpm) or >120 bpm
- Unusual or severe shortness of breath
- Chest pain, including chest discomfort or pressure, left arm pain, report of indigestion or stomach discomfort
- Palpitations
- Fainting, light headedness, dizziness

A supervised session may also be terminated if participants complain of musculoskeletal pain that contraindicates continued safe exercise.

6.6 Temporary Intervention Discontinuation

There may be situations that arise that will warrant temporary discontinuation of the intervention for a participant. When these events occur, the Intervention Oversight Team, Y-PM, Trainer, and Clinic YMCA Liaison will develop a plan during twice per month meetings to keep the participant engaged in the study while 'on hold' and to reengage the participant once the situation has resolved. The decision to remove a participant from the study in the event of a prolonged Temporary Intervention Discontinuation will be made by the Intervention Oversight Team, in consultation with the site PI and the ADCS, on a case-by-case basis.

A participant will be classified as being medically 'on hold' if 4 or more consecutive exercise sessions are missed due to self-reported hospitalization, injury or other health issue (vacations excluded), or is withdrawn from physical activity as per primary care provider (PCP) orders. If a participant is on hold, the Trainer, Y-PM, or Clinic YMCA Liaison will contact the participant weekly to obtain updates about status and expected schedule when exercise can be resumed.

Restart following soft tissue injuries without surgery can occur when the participant self-reports an ability to continue the intervention. Restart following other medical complications will require prior approval by the Study Clinician. The site PI may choose to obtain the participant's PCP approval for re-start as well. Communication with participants on medical leave regarding their status in the study should always emphasize that they are 'on hold' rather than 'temporarily suspended' or 'on leave' to encourage continued psychological/emotional commitment to the study, which will likely expedite restart when appropriate to do so.

6.7 Reasons for Temporary Discontinuation

Reason(s) for temporary intervention discontinuation will be captured in the appropriate CRF and coded as follows:

- **Adverse experience**: The participant has experienced an AE that, in the opinion of the

investigator, requires temporary intervention discontinuation; this may include abnormal laboratory values.

- **Safety risk**: Any participant who is deemed a safety risk by the investigator due to a temporary condition.
- **Study temporarily suspended** by the ADCS.
- **Investigator judgment** that it is in the participant's or study's best interest to temporarily discontinue the intervention until ongoing personal or medical issues no longer interfere with participation, including unanticipated prolonged travel or medical issues (e.g., surgery, accidents, development of new medical conditions), and family matters.
- **Intervention Oversight Team judgment** that it is in the participant's or study's best interest to temporarily discontinue the intervention until ongoing personal or medical issues no longer interfere with participation, including unanticipated prolonged travel or medical issues (e.g., surgery, accidents, development of new medical conditions), and family matters.

6.8 Timing of Temporary Discontinuation Relative to Assessment of Primary and Secondary Outcomes

If a temporary intervention discontinuation occurs near the time of the scheduled Month 6 or Month 12 clinic assessment, the acceptable window within which to complete this visit may be expanded by up to 6 weeks if this would allow participants to resume the intervention at the prescribed dose for at least 3 weeks prior to the assessment. If longer delays are required to meet this goal, the assessment visit will be completed according to the original schedule. The decision to postpone an assessment visit will be made by the Intervention Oversight Team, in consultation with the site PI and the ADCS, on a case-by-case basis.

6.9 Blinding

The Study Clinician and the Clinic YMCA Liaison will be unblinded to intervention group assignment; all other clinic staff will remain blinded. The Study Clinician will be responsible for reviewing and following up on all AEs. The Clinic YMCA Liaison serves as the primary interface between the participating YMCA and the clinic, and thus will be exposed to intervention-specific details for participants. The description of TEAEs may relate to physical activities completed as part of the assigned intervention. Disclosure of randomization assignment will occur in the clinic by either the Clinic YMCA Liaison or the Study Clinician.

It will be critically important that the clinic staff member(s) who administer the cognitive and functional assessment instruments to participants (referred to as 'Raters') remain blinded. Unblinded staff will not discuss any details about a participant's intervention or progress with the Rater, and the Rater will not have access to any intervention-related data, including AEs, supervised exercise adherence data, or participant Physical Activity Logs. Participants will be instructed by the Clinic YMCA Liaison at the start of each clinic visit to refrain from sharing any information about their assigned intervention with the Rater or other clinic staff (other than the Study Clinician). Signs will be clearly posted in all testing rooms to remind participants to not

share information about their assigned exercise program, and Raters will also remind participants to keep this information to themselves at frequent intervals. These procedures to ensure that the Rater remains blinded to intervention assignment have been used with success in other randomized clinical trials of aerobic exercise in non-demented older adults.[16,86]

The Wake Forest team and the Y-USA will remain unblinded so that they may assist the Trainers, Y-PMs, and Clinic YMCA Liaisons with intervention-specific challenges as they arise. The Project Directors will remain blinded to primary and secondary outcomes by intervention group assignment.

6.10 Breaking the Blind

Decisions regarding breaking the blind for clinic personnel other than the Clinic YMCA Liaison and Study Clinician must be made in consultation with the PDs and the ADCS Medical Director. If the blind is broken for other clinic staff members in any instance, the site PI must document the following information on a Blind Break Notification Form for the participant: date, personnel exposed, and why/how blind was broken. An alternate plan for assessment of primary outcomes at future clinic visits may be requested by the PDs and the ADCS.

6.11 Concomitant AD Medications

Stable use of memantine or cholinesterase inhibitors at study entry will be permitted (3 or more months at a consistent dose with no plans to modify). Participants who start AD medications after enrollment will be permitted to continue in the study.

7.0 STUDY POPULATION

A total of 300 adults diagnosed with test scores and clinical ratings consistent with amnestic MCI will be enrolled into EXERT. To determine eligibility, all participants will undergo cognitive assessment, physical and neurological examination, electrocardiogram (ECG), clinical/safety laboratory assessment, and interviews of the participant and study partner.

7.1 Inclusion Criteria

1. Age between 65 and 89 years old, inclusive
2. MMSE: ≥24 for participants with 13 or more years of education; ≥22 for participants with 12 or fewer years of education
3. Global CDR score of 0.5 with a memory score of at least 0.5
4. Profile of test scores and clinical ratings is consistent with amnestic mild cognitive impairment
5. Speaks English fluently
6. Visual and auditory acuity adequate for cognitive testing
7. Completed at least 6 years of formal education or work history sufficient to exclude mental retardation

8. Has an informant who knows the participant well, has regular contact, and is available to accompany the participant to clinic visits or complete study partner assessments remotely.
9. Sedentary or underactive, determined by responses to the staff-administered EXERT Telephone Assessment of Physical Activity (TAPA) survey
10. Willing to be randomized to either intervention group and to complete the assigned activities as specified for 18 months
11. Willing and able to reliably travel to the identified YMCA, 4 times per week for 18 months
12. Ability to safely participate in either intervention and complete the 400 m Walk Test within 15 min without sitting or use of any assistance
13. Plans to reside in the area for at least 18 months
14. For planned travel, total time away must be no more than 2 months over the course of the study, and no more than 1 month at any one time; participants must be willing to continue the assigned exercise program if travelling out of the area for more than 1 week
15. In overall good general health with no disease or planned surgery that could interfere with study participation
16. Modified Hachinski ≤4
17. Stable use of cholinesterase inhibitors, memantine, vitamin E, estrogens, aspirin (81-300 mg daily), beta-blockers, or cholesterol-lowering agents for 12 weeks prior to screening (important for biomarker analyses)
18. Stable use of antidepressants lacking significant anticholinergic side effects for 4 weeks prior to screening as long as the participant does not meet DSM V criteria for major depression currently or in the last 12 months; GDS scores are to be used to inform clinical decisions but there is no specified cut-off score for inclusion
19. When applicable, willing to complete 4-week washout of psychoactive medications, including disallowed antidepressants, neuroleptics, chronic anxiolytics or sedative hypnotics, and willing to avoid these medications for the duration of the trial
20. Able to complete all baseline assessments

7.2 Exclusion Criteria

1. Any significant neurologic disease, other than MCI, including any form of dementia, Parkinson’s disease, Huntington’s disease, normal pressure hydrocephalus, brain tumor, progressive supranuclear palsy, seizure disorder, subdural hematoma, multiple sclerosis, or history of significant head trauma with persistent neurologic sequelae or known structural brain abnormalities
2. Sensory or musculoskeletal impairment sufficient to preclude successful and safe completion of the intervention or assessment protocols; must be able to walk safely and unassisted on a treadmill
3. Contraindications for MRI studies, including claustrophobia, metal (ferromagnetic) implants, or cardiac pacemaker
4. Brain MRI at screening shows evidence of infection, infarction, or other clinically significant focal lesions, including multiple lacunes in prefrontal or critical memory regions; inconclusive findings may be subject to review by the ADCS Imaging Core

5. History of major depression or bipolar disorder (DSM V criteria), psychotic features, agitation or behavioral problems within the last 12 months
6. History of schizophrenia, as per DSM V criteria
7. History of alcohol or substance abuse or dependence within the past 2 years, as per DSM V criteria
8. Currently consumes more than 3 alcoholic drinks per day
9. Clinically significant or unstable medical condition, including uncontrolled hypertension or significant cardiac, pulmonary, hematologic, renal, hepatic, gastrointestinal, endocrine, metabolic or other systemic disease in the opinion of clinic medical personnel that may put the participant at increased risk, influence the results or compromise the participant's ability to participate in the study (treated atrial fibrillation for more than 1 year or occasional premature ventricular contractions on ECG are not exclusions)
10. History in the last 6 months of myocardial infarction, coronary artery angioplasty, bypass grafting, or STENT placement
11. History in the last 3 months of transient ischemic attack or small vessel stroke (if more than 3 months, small vessel stroke with no residual effects are permitted)
12. Expected joint replacement surgery within the next 18 months
13. History within the last 5 years of a primary or recurrent malignant disease with the exception of non-melanoma skin cancers, resected cutaneous squamous cell carcinoma in situ, basal cell carcinoma, cervical carcinoma in situ, or in situ prostate cancer with normal prostate-specific antigen posttreatment
14. Hemoglobin A1c >7.0
15. Clinically significant abnormalities in screening laboratory blood tests: low B12 is exclusionary, unless follow-up labs (homocysteine [HCY] and methylmalonic acid [MMA]) indicate that it is not physiologically significant
16. Current or past use of insulin to treat type 2 diabetes (other diabetes medications are acceptable if hemoglobin A1c ≤7)
17. Current use (within 60 days of screening) of psychoactive medications including tricyclic antidepressants, antipsychotics, mood-stabilizing psychotropic agents (e.g. lithium salts), psychostimulants, opiate analgesics, antiparkinsonian medications, anticonvulsant medications (except gabapentin and pregabalin for non-seizure indications), systemic corticosteroids, or medications with significant central anticholinergic activity. Limited use of antipsychotics (quetiapine ≤ 50mg/day or risperidone ≤ 0.5mg/day), and non-chronic use of opiate analgesics on an as needed basis is permitted; such medications must be avoided for 8 hours before clinic assessments
18. Chronic use of anxiolytics or sedative hypnotics except as follows: use of benzodiazepines for treatment on an as-needed basis for insomnia or daily dosing of anxiolytics is permitted; medications must be avoided for 8 hours before clinic assessments
19. Previous or current treatment involving active immunization against amyloid
20. Previous treatment with approved or investigational agents with anti-amyloid properties or passive immunization against amyloid are prohibited 12 months prior to screening and for the duration of the trial; treatment with other investigational agents are prohibited 3 months prior to screening and for the duration of the trial
21. For LP, current use of anticoagulants such as Coumadin, Plavix, or high dose Vitamin E

22. For LP, current blood clotting or bleeding disorder, or significantly abnormal prothrombin time (PT) or partial thromboplastin time (PTT) at screening
23. For LP, presence of physical distortions due to spinal surgery, severe degenerative joint disease or deformity, or obesity that could interfere with CSF collection (as per investigator judgment)
24. Participants whom the PI deems otherwise ineligible

7.3 Recruitment and Retention Strategies

A recruitment plan will be developed in coordination with the ADCS and its study partners. The plan will include national media releases and EXERT-specific study materials that can be used by the clinics to recruit participants upon receipt of local IRB approval to do so. Multiple strategies will be used to promote high rates of retention, including twice weekly meetings with the Trainer, opportunities to participate in group exercise activities, and by providing tangible reinforcers as participants advance through the study (e.g., quilts, T-shirts, exercise accomplishment reports).

7.4 Inclusion of Women and Minorities

No subject will be excluded for reasons of sex, race, or ethnic group. There are currently no studies that definitively support or negate differences in response to aerobic exercise within these subgroups. Consistent with the aims of the ADCS Minority Recruitment Core, EXERT will target a minimum of 20% racial/ethnic minority representation in the sample of 300 older adults.

8.0 STUDY TIMELINE

Study participation will include up to 6 weeks between Screening II (Visit 1) and Baseline (Visit 2), 12 months of supervised exercise at a local YMCA facility, and 6 months of unsupervised exercise at the YMCA.

9.0 DESCRIPTION OF STUDY VISITS

The schedule of events is provided in **Appendix 1**.

9.1 Screening I (Visit 0)

Prior to the in-person screening evaluation, the investigator will identify potential participants through routine clinical contact or an additional screening process. Prospective participants will have a subjective memory complaint and/or demonstrate memory impairments during screening, will be considered ‘sedentary’ as assessed by the EXERT TAPA, will meet all other inclusion/exclusion criteria, and will agree to be randomized to either the SBR or AX group. Study candidates may provide verbal consent over the telephone to complete the EXERT TAPA and other screening questions regarding demographics, medical history, and medications to assess eligibility for continued screening, if permitted by the local IRB. Study candidates and

their collaterals will not be considered as EXERT participants until the core study consent form is signed at an in-person screening visit.

9.2 Screening II (Visit 1)

This assessment will determine eligibility to enroll in EXERT, and may be conducted over multiple days. Potential participants and their study partners must sign an informed consent form (ICF) and Health Insurance Portability and Accountability Act (HIPAA) authorization prior to administration of any procedures for this assessment. Screening II procedures will include the following:

- ICF/HIPAA: participant and study partner
- Review of inclusion/exclusion criteria (includes review of EXERT TAPA responses)
- Demographics review
- Medical history
- Medication review
- Brief physical examination, vital signs, weight, height
- Brief neurological examination
- Modified Hachinski
- Blood collection for ApoE genotyping, and DNA banking
- Blood collection for clinical labs
- 12-lead resting ECG
- Cognitive assessment
    - 3MSE/MMSE
    - Logical Memory I and II
    - Auditory Verbal Learning Test (AVLT)
- Behavioral/functional assessment
    - CDR
    - Cognitive Change Index (self-administered)
    - GDS (self-administered)
- 400 m Walk Test
- Brain MRI
    - For participants meeting all other eligibility criteria (as determined by site investigator)
    - Participants may be excluded from enrollment if safety concerns are identified
    - At approved sites, protocol will include EXERT fMRI sequences
- Dispense ActiGraph Link for actigraphy data collection
    - Dispense following completion of screening procedures to participants who meet eligibility criteria and thus are at high probability of randomization
    - Device should be placed on the participant's non-dominant wrist at the clinic to initiate the recording period
    - Device should continuously remain on the participant's wrist for the duration of the 10-day recording period

  - Retrieve ActiGraph Link device 10 days later during a baseline visit or by mail (with a reminder call on Day 10), and upload data
  - Data collection should be completed prior to randomization

The participant will be permitted to proceed to the baseline visit if all inclusion/exclusion criteria are met, results from all screening procedures are reviewed, and eligibility have been approved by the site investigator. The PDs, in consultation with the ADCS Medical Director, must approve any exceptions or questions regarding possible exclusionary medications, medical conditions, or laboratory tests. In addition, the PDs must approve any exceptions to cognitive eligibility criteria in cases where there is compelling evidence for MCI but not all cognitive eligibility criteria are met.

The baseline visit should be scheduled within 6 weeks of Screening II. A re-screen is allowed in the event of a screen failure, but must occur more than 90 days later. If the baseline visit is scheduled more than 6 weeks from Screening II (i.e., out-of-window), approval to proceed to the baseline visit must be obtained from the ADCS and a rescreen may be required.

9.3 Baseline (Visit 2)

Baseline procedures may be completed over multiple days, but all assessments should be completed within 14 days, with the condition that cognitive and behavioral assessments should not be administered while participants are fasting. Cognitive testing conducted after an LP must be administered at least 48 hours following the procedure. All baseline assessments must be completed before randomization and start of exercise, and include:

- Review of inclusion and exclusion criteria
- Medication review
- Vital signs, weight
- Fasting blood collection for banking
- AE query
- Cognitive assessment
  - Primary outcomes assessment (administer the following tests before administering other cognitive/behavioral instruments)
    - ✓ ADAS-Cog13
    - ✓ Trail-Making Test
    - ✓ DSST
    - ✓ Category and Letter Word Fluency
  - Secondary outcomes assessment (iPad computer tests)
    - ✓ Cogstate: Detection task (DET), Identification task (IDN), One Card Learning (OCL), One Back task (OBK), Groton Maze Learning Test (GMLT), Face Name Associative Memory (FNAME), Behavioral Pattern Separation of Objects (BPSO)
    - ✓ NIH Toolbox: Flanker, Dimensional Change Card Sort (DCCS)
- Behavioral/functional and other outcomes assessments

- Staff-administered questionnaires
    - ✓ ADCS-ADL-MCI
    - ✓ BRIEF-A
    - ✓ NPI
- Self-administered questionnaires
    - ✓ By participant
        - ➢ SF-36
        - ➢ EQ5D
        - ➢ Research Satisfaction Survey
    - ✓ By study partner
        - ➢ Study Partner Self-Assessment Questionnaire (SF-36, Pittsburgh Sleep Quality Index, Insomnia Severity Index, questions regarding healthcare utilization)
        - ➢ EQ5D (as it relates to participant)

- LP
    - For participants who agree to the LP
    - Follow-up telephone call by staff within 24 hours to query AEs
- Randomization
    - Eligible participants will be randomized to either the SBR or AX group centrally by the ADCS using a real-time web-based randomization system
    - Designated unblinded clinic staff member will disclose this assignment to the participant, in person, when all baseline assessments have been completed
    - Participants will be provided with a packet of materials describing their assigned intervention, their responsibilities to the study, and what the study will provide for them. At a YMCA appointment, Trainers will provide participants with a HR monitor, the first Physical Activity Log, and the appropriate Activity Resource Guide.
    - Participants will be instructed to maintain their regular diet and other activities for the duration of the study
    - At time of disclosure, and once the provided materials have been carefully reviewed with the participant, the participant will be asked to sign a document indicating his/her willingness to complete the assigned programs as instructed, for the duration of the 18-month trial
    - Participants will be provided with the name and contact information of the site Y-PM who will follow-up within 3 weekdays to schedule the first appointment at the YMCA, which should be completed within 2 weeks of the last baseline visit
    - Participants will be instructed to withhold all information about intervention group assignment from all other clinic staff members throughout study participation; the importance of keeping this ‘secret’ will be emphasized and discussed
- At Site PI discretion, an optional letter can be provided to notify the participant’s primary care physician of participant enrollment in the study.

Beginning in Month 1, Clinic YMCA Liaison will complete a monthly telephone call to query AEs, obtain medication updates, provide encouragement, and problem-solve challenges to continued participation.

9.4 Month 6 (Visit 3)

The Month 6 assessment should be completed 6 months (+/- 2 weeks) from the participant's first appointment at the YMCA that initiated the intervention. Participants will be asked to bring their HR monitor to the appointment and to refrain from exercise for 24 hours prior to cognitive testing, which should not be completed while participants are fasting. Month 6 procedures include:

- Fasting blood collection for banking
- Medication review
- Brief physical exam, vital signs, weight
- Brief neurological exam
- AE query
- Briefly review adherence with participant using report provided through EDC and entries in the Physical Activity Logs (adherence will be regularly monitored by the YMCA Trainer, the Y-PM and the Intervention Oversight Team as well)
- Cognitive assessment
    - Primary outcomes assessment (administer the following tests before administering other cognitive/behavioral instruments)
        - ✓ ADAS-Cog13
        - ✓ Trail-Making Test
        - ✓ DSST
        - ✓ Category and Letter Word Fluency
    - Secondary outcomes assessment (iPad computer tests)
        - ✓ Cogstate: DET, IDN, OCL, OBK, GMLT, FNAME, BPSO
        - ✓ NIH Toolbox: Flanker, DCCS
- Behavioral/functional and other outcomes assessments
    - Staff-administered questionnaires
        - ✓ CDR
        - ✓ ADCS-ADL-MCI
        - ✓ BRIEF-A
        - ✓ NPI
    - Self-administered questionnaires
        - ✓ By participant
            - ➢ Cognitive Change Index
            - ➢ SF-36
            - ➢ EQ5D
            - ➢ Research Satisfaction Survey
        - ✓ By study partner

- Study Partner Self-Assessment Questionnaire (SF-36, Pittsburgh Sleep Quality Index, Insomnia Severity Index)
- EQ5D (as it relates to participant)

- 400 m Walk Test
- Dispense ActiGraph Link for actigraphy data collection
    - Place device on the participant's non-dominant wrist to initiate recording period
    - Device should continuously remain on the participant's wrist for the duration of the 10-day recording period
    - Retrieve device from participant 10 days later by return mail (with a reminder call on Day 10), and upload data when device is received
- Replace battery in HR monitor
- Provide participant with 6-month supply of Physical Activity Logs

The Clinic YMCA Liaison will continue to complete monthly telephone calls to query AEs, obtain medication updates, provide encouragement, and problem-solve challenges to continued participation.

9.5 Month 12 (Visit 4) / or Early Termination

The Month 12 assessment will provide the primary endpoints to evaluate trial efficacy, and should be completed 12 months (+/- 2 weeks) from the participant's first appointment at the YMCA that initiated the intervention. The following procedures may be completed over multiple days, but within a 7-day window. Participants will be instructed to bring their HR monitor to the appointment and to refrain from exercise for 24 hours prior to cognitive testing, fMRI (for approved sites), and the LP. Cognitive testing should not be completed while participants are fasting, and if conducted after an LP, testing must be administered at least 48 hours following the procedure. Month 12 procedures include:

- Fasting blood collection for clinical labs
- Fasting blood collection for banking
- Medication review
- Brief physical exam, vital signs, weight
- Brief neurological exam
- AE query
- Briefly review adherence with participant using report provided through EDC and entries in the Physical Activity Logs (adherence will be regularly monitored by the YMCA Trainer, the Y-PM and the Intervention Oversight Team as well)
- Cognitive assessment
    - Primary outcomes assessment (administer the following tests before administering other cognitive/behavioral instruments)
        - ✓ ADAS-Cog13
        - ✓ Trail-Making Test
        - ✓ DSST
        - ✓ Category and Letter Word Fluency

- Secondary outcomes assessment
  - ✓ 3MSE/MMSE
  - ✓ iPad computer tests
    - ➢ Cogstate: DET, IDN, OCL, OBK, GMLT, FNAME, BPSO
    - ➢ NIH Toolbox: Flanker, DCCS

- Behavioral/functional and other outcomes assessments
  - Staff-administered questionnaires
    - ✓ CDR
    - ✓ ADCS-ADL-MCI
    - ✓ BRIEF-A
    - ✓ NPI
  - Self-administered questionnaires
    - ✓ By participant
      - ➢ Cognitive Change Index
      - ➢ SF-36
      - ➢ EQ5D
      - ➢ Research Satisfaction Survey
    - ✓ By study partner
      - ➢ Study Partner Self-Assessment Questionnaire (SF-36, Pittsburgh Sleep Quality Index, Insomnia Severity Index)
      - ➢ EQ5D (as it relates to participant)
- 400 m Walk Test
- Brain MRI
  - At approved sites, protocol will include fMRI sequences
- LP
  - CSF may be collected at this visit, even if not successfully collected at Baseline
  - Follow-up telephone call by staff within 24 hours to query AEs
- Dispense ActiGraph Link for actigraphy data collection
  - Place on the participant's non-dominant wrist to initiate recording period
  - Device should continuously remain on the participant's wrist for the duration of the 10-day recording period
  - Retrieve device from participant 10 days later by return mail (with a reminder call on Day 10), and upload data when device is received
- Replace battery in HR monitor
- Provide participant with 6-month supply of Physical Activity Logs
- Site PI and Raters to complete Treatment Blinding Questionnaire

All participants will transition to unsupervised exercise during the last 6 months of the study. Participants will be instructed to continue their assigned intervention at the specified frequency, duration, and intensity, and to continue to track their exercise using their Physical Activity Log. During this phase of the study, the Clinic YMCA Liaison will continue to contact participants by telephone, once per month to query AEs, obtain medication updates, provide encouragement, and problem-solve challenges to continued participation.

9.6 Month 18 (Visit 5)

The Month 18 assessment should be completed 18 months (+/- 2 weeks) from the participant's first appointment at the YMCA that initiated the intervention. Participants will be instructed to bring their HR monitor to the appointment and to refrain from exercise for 24 hours prior to cognitive testing, which should not be completed while fasting. Participants should also be asked to return all Physical Activity Logs for review and data entry, as this will be their final EXERT assessment. Month 18 procedures include:

- Fasting blood collection for clinical labs
- Fasting blood collection for banking
- Medication review
- Brief physical exam, vital signs, weight
- Brief neurological exam
- AE query
- Review returned Physical Activity Logs to ensure that they were completed and accurately reflect YMCA attendance records (provided in advance) and the participant's self-report
- Dispense ActiGraph Link for actigraphy data collection
    - Send device and instructions to participants 14 days before the Month 18 assessment; confirm with participant that the device was received and in place with a phone call at the start of the recording period
    - At the clinic visit, remove the device from the participant's wrist and upload data
- Cognitive assessment
    - Primary outcomes assessment (administer the following tests before administering other cognitive/behavioral instruments)
        - ✓ ADAS-Cog13
        - ✓ Trail-Making Test
        - ✓ DSST
        - ✓ Category and Letter Word Fluency
    - Secondary outcomes assessment (iPad computer tests)
        - ✓ Cogstate: DET, IDN, OCL, OBK, GMLT, FNAME, BPSO
        - ✓ NIH Toolbox: Flanker, DCCS
- Behavioral/functional and other outcomes assessment
    - Staff-administered questionnaires
        - ✓ CDR
        - ✓ ADCS-ADL-MCI
        - ✓ BRIEF-A
        - ✓ NPI
    - Self-administered questionnaires
        - ✓ By participant
            - ➢ Cognitive Change Index
            - ➢ SF-36
            - ➢ EQ5D

        - Research Satisfaction Survey
    - By study partner
        - Study Partner Self-Assessment Questionnaire (SF-36, Pittsburgh Sleep Quality Index, Insomnia Severity Index)
        - EQ5D (as it relates to participant)
- 400 m Walk Test
- Provide study close-out materials
    - Summary of progress in intervention
    - Fasting clinical blood test results from all visits
    - Color printout of single brain MRI image if available
    - Study completion certificate
    - ‘Thank-you’ gift or payment for participation
    - Participant’s HR monitor (change battery)
    - Contact information for a YMCA staff member who will provide a personal coaching session at the YMCA, at no cost
- Site PI and Raters to complete Treatment Blinding Questionnaire

10.0 EARLY INTERVENTION DISCONTINUATION

Investigators at each site will make every effort to maximize participant retention. If a participant expresses a desire to stop the intervention, the Intervention Oversight Team will be notified and will hold a conference call with the Clinic YMCA Liaison and/or site investigator, and Y-PM and/or Trainer to discuss the participant’s existing challenges and possible approaches to reduce burden to encourage continued participation if at all possible (e.g., adapt exercise routine to reduce pain or increase interest, problem-solve regarding transportation issues, etc.).

If a participant continues to decline further participation, or if an investigator discontinues the intervention, an Early Discontinuation Visit should be completed as soon as possible just prior to or immediately following intervention discontinuation. The Early Discontinuation Visit will include all Month 12 assessments to permit collection of primary and secondary outcome measures. If this Early Discontinuation Visit occurs before the start of Month 4, then the MRI and LP (when applicable) should not be completed. Alternatively, if an in-person Early Discontinuation Visit cannot be completed, site personnel should complete, by telephone, as many of the Month 12 procedures as possible (e.g., medication review, AE query, request return of Physical Activity Logs for review and data entry, CDR, Cognitive Change Index, SF-36, ADCS-ADL-MCI, BRIEF-A, NPI, Research Satisfaction Survey, EQ5D, Study Partner Self-Assessment Questionnaire).

All early intervention discontinuation participants will be strongly encouraged to complete not only the Early Discontinuation Visit, but also all regularly scheduled clinic assessments through Month 18.

10.1 Reasons for Early Discontinuation

Reason(s) for intervention discontinuation will be coded as follows:

- **Perceived lack of efficacy** by participant
- **Adverse experience by participant** that, in the opinion of the investigator, requires early termination; this may include abnormal laboratory values
- **Participant becomes a safety risk**
- **Death**
- **Participant is unwilling or unable to participate**
- **Participant is not able to adhere to protocol requirements** as per judgment of the Intervention Oversight Team or site investigator
- **Study partner unwilling or unable to participate, and new appropriate study partner cannot be identified**
- **Lost to follow up**: Participant could not be recalled to the clinic for follow-up assessments
- **Participant enrolls in another intervention trial**
- **EXERT is terminated**
- **Investigator judgment** that it is in the participant’s best interest to discontinue participation
- **ADCS request**: the ADCS determines that it is in the participant’s best interest to discontinue participation in the study
- **Intervention Oversight Team request**: the Intervention Oversight Team determines that it is in the participant’s best interest to discontinue participation in the study

11.0 STUDY-SPECIFIC INSTRUMENTS

11.1 EXERT Telephone Assessment of Physical Activity

The TAPA is a brief, validated, and commonly administered telephone-based measurement tool to assess physical activity in older adults.[87] This protocol uses a modified version, adapted for EXERT with permission of the original authors.

11.2 Cognitive Evaluations

11.2.1 Modified Mental State Examination

The 3MSE, an expanded version of the MMSE, has been widely used in epidemiologic cohort studies to assess global cognitive function.[88] The 3MSE consists of items assessing 8 domains of cognitive function (remote memory, recent memory, mental control, time and space orientation, abstract thinking, verbal abilities, verbal comprehension and spatial cognition) and is scored on a 100-point scale with higher scores indicating better performance. The MMSE[89] is a

brief, frequently used screening instrument for AD drug studies and includes several shared items with the expanded version.

11.2.2 Logical Memory

Logical Memory I and II (immediate and delayed recall) is a modification of the episodic memory subtest from the Wechsler Memory Scale-Revised (WMS-R).[90] In this version, free recall of one short story consisting of 25 bits of information will be elicited immediately after it is read aloud to the participant and again after a 20-30 minute delay. Total bits of information from the story that are recalled immediately (maximum score = 25) and after the delay (maximum score = 25) are recorded.

11.2.3 Auditory Verbal Learning Test

The AVLT is a list learning task with multiple outcomes to assess encoding, consolidation, storage, and retrieval of verbal information.[91] On each of 5 learning trials, 15 unrelated nouns are presented orally at the rate of one word per second and immediate free recall of the words is elicited. Number of correctly recalled words on each trial is recorded. A distractor list is then presented, after which participants are asked to recall words from the first list (Trial 6). Following a 20-30minute delay filled with unrelated testing, free recall of the first 15-item word list (Trial 7) is again elicited.

11.2.4 ADAS-Cog-Exec Global Composite

Select subscales of ADAS-Cog13, tests of executive function, CDR sum of select box scores.[49]

ADAS-Cog13. The ADAS-Cog[92] is a structured scale that evaluates memory (word recall, word recognition), reasoning (following commands), language (naming, comprehension), orientation, ideational praxis (placing letter in envelope) and constructional praxis (copying geometric designs). Ratings of spoken language, language comprehension, word finding difficulty, and ability to remember test instructions are also obtained. The test is scored in terms of errors, with higher scores reflecting poorer performance and greater impairment. Scores can range from 0 (best) to 70 (worse) and then number of items not recalled ranging from 0-10 is added for a maximum score of 80. The 13-item version of the ADAS-Cog will be administered in EXERT. Different Raters should administer the ADAS-Cog13 and the CDR. The ADAS-Cog-Exec global composite includes ADAS-Cog13 Word Recall, Delayed Word Recall, Orientation, and Number Cancellation.

Trail-Making Test (Trails A and B). Trails A[89] consists of 25 circles numbered 1 through 25 distributed over a white sheet of paper. The participant is instructed to draw a line to connect the circles in ascending numerical order as quickly as possible (150 second maximum). Trails B consists of 25 circles containing either numbers (1 through 13) or letters (A through L) that are randomly distributed across the page, and participants are instructed to connect the circles in alternating and ascending order (e.g., 1 to A; 2 to B). Time to complete Trails B (300 second maximum), adjusted for the time taken to complete Trails A to control for sensorimotor demands

of the task, is a sensitive measure of executive function and working memory, and the effects of aerobic exercise.[16,20] The ADAS-Cog-Exec global composite includes time to completion for parts A and B of the Trail Making Test.

Digit Symbol Substitution Test. The DSST[89] is a subset from the Wechsler Adult Scale of Intelligence-*Revised* (WAIS-R). The test consists of 110 small blank squares presented in 7 rows with 1 of 9 numbers (1-9) randomly printed directly above each blank square. A legend is printed above the rows of blank squares, at the top of the page. The legend pairs each of the numbers with an abstract symbol. Following a few practice trials, the participant is asked to use the legend to fill in the blank squares in consecutive order, working from left to right across the rows, with the symbol that is paired with each number and as quickly as possible for 90 seconds. Number correct is recorded (maximum score =110). This test engages multiple abilities including attention, psychomotor speed, visual scanning and tracking, and working memory. The ADAS-Cog-Exec global composite scale includes the total score for the Digit Symbol Substitution Test.

Word Fluency. This 2-part task assesses word fluency by letter and by category, and thus involves planning, organization, and cognitive flexibility. Participants are asked to generate as many words as possible in 60 seconds that begin with different letters of the alphabet (f and L), in 2 separate trials. Participants are then asked to generate as many words as possible in 60 seconds that belong to a given semantic category (animals, vegetables). Reduced word fluency is associated with AD onset and progression,[93] and was sensitive to the effects of aerobic exercise in preliminary studies.[16,20] The ADAS-Cog-Exec global composite includes the score for Category Fluency (mean of Animal and Vegetable Fluency).

Clinical Dementia Rating Scale – Sum of Boxes. The CDR[89] is a clinical scale that rates the severity of dementia as absent, questionable, mild, moderate, or severe (CDR score of 0, 0.5, 1, 2, or 3, respectively). The score is based on interviews with the participant and study partner, using a structured interview that assesses 6 domains: memory, orientation, judgment and problem solving, community affairs, home and hobbies, and personal care. The ratings of degree of impairment for each of the 6 domains are synthesized into one global rating of dementia (ranging from 0 to 3), with more refined measure of change available with the Sum of Boxes. At screening, the CDR global and memory scores will be used to assess eligibility. For all other administrations, scores across the 6 domains will be summed to obtain the Sum of Boxes score. Different Raters should administer the CDR and the ADAS-Cog13. The ADAS-Cog-Exec global composite includes the box scores for CDR Memory, Orientation, and Judgement and Problem Solving.

11.2.5 Executive Function Composite

The Executive Function Composite will combine 8 measures of executive control using performance outcomes on paper-and-pencil tests (described above: Trails B, DSST, Word Fluency, Number Cancellation), and iPad tests from the NIH Toolbox (Flanker, DCCS), and

Cogstate (GMLT, OBK). NIH Toolbox and Cogstate tests will be loaded on the same iPad and accessed through different icons on the screen.

NIH Toolbox Flanker.[94] This speeded test measures attention and inhibitory control. The participant is asked to focus on one stimulus (arrow in the center of the screen) while inhibiting attention other stimuli (flanking arrows). Sometimes the central stimulus is pointing in the same direction as the flankers (congruent) and sometimes in the opposite direction (incongruent). Scoring is based on a combination of reaction time and accuracy. The test takes approximately 3 minutes to administer.

NIH Toolbox Dimensional Change Card Sort.[94] The DCCS has been validated in older adults, is sensitive to the effects of aging, and performance is highly correlated with scores on standardized tests of cognitive flexibility (i.e., Color-Word Interference Inhibition of the Delis-Kaplan Executive Function Scales). In this speeded test, 2 target pictures are presented that vary along 2 dimensions (i.e., shape, color). Participants are asked to match a series of bivalent test pictures (e.g., yellow balls, blue trucks) to the target pictures, first according to one dimension (e.g., color) and then, after a number of trials, according to the other dimension (e.g., shape). “Switch” trials are also included, in which the participant must change the dimension being matched. For example, after 4 trials matching on shape, the participant may be asked to match on color for the next trial and then match on shape again for subsequent trials. Scoring is based on a combination of reaction time and accuracy. The test takes approximately 4 minutes to administer.

Cogstate Groton Maze Learning Test (Pediatric Version).[95,96] The GMLT is a test of executive function assessing visual/spatial working memory, learning, and error monitoring. The test consists of an 8 x 8 grid of grey tiles presented on an iPad screen. To find the hidden path from one corner of the grid to another, the participant touches one tile at a time starting in the top left corner, and responds to feedback about whether the touched tile belongs to the path, which is indicated by a ‘▯’ if correct, and an ‘**X**’ if incorrect. If the move is correct, the ▯remains visible on the tile and the participant touches another adjacent square. If the touch is incorrect, the **X** on the tile disappears and participants must touch the last correct tile in the path before continuing. The trial ends once the participant successfully touches all tiles in the hidden path that leads to the flag in the bottom right corner of the grid. Total time for test administration is approximately 7 minutes.

Cogstate One Back.[57] The OBK is a test of attention and working memory. In this task, a playing card is presented face up in the center of the screen and starting with trial 2, the participant must decide whether it is identical to the card presented in the previous trial by responding with a finger touch. Performance on the Cogstate DET and IDN tasks will be used to adjust OBK performance for simple and choice reaction time. Time to administer all 3 tasks is 6 minutes (2 minutes per task).

11.2.6 Episodic Memory Composite

The Episodic Memory Composite combines several measures of short-term memory including Delayed Word Recall from the ADAS-Cog13 and four Cogstate tasks administered via iPad (FNAME, BPSO, OCL, GMLT-Delayed Recall).
Cogstate Face-Name Associative Memory Exam. FNAME pairs 180 pictures of unfamiliar faces with common first names (5 lists of 36 trials), and engages memory networks, including the hippocampus and default network regions as per fMRI findings.[97-99] During encoding, participants are instructed to remember each face-name pair presented on the screen and to rate how well the name ‘fits’ the face using a Likert scale. During retrieval, participants are asked to recall the correct name associated with each face, and subsequently, to select the correct name per face from options presented on the screen. For both tasks, accuracy is recorded. Test performance is sensitive to physical activity in older adults.[100]

Cogstate Behavioral Pattern Separation of Objects.[101] This visual recognition memory test displays pictures on the iPad screen of common objects interspersed with highly similar “lures” that vary in levels of mnemonic and visual similarity with target items. During encoding, participants indicate whether pictures were of “indoor” or “outdoor” objects by touching a ‘button’ on the screen. During the test phase, participants view objects that include exact replicas of previously viewed objects, similar but not identical objects, and new objects, and are asked to judge whether each object is “old,” “similar,” or “new” using a ‘button’ press. Higher accuracy reflects better separation of the visual patterns, and thus better memory. The BPSO robustly activates the hippocampus in fMRI studies, and is sensitive to early dysfunction in the hippocampus in aging,[102-104] and to therapeutic effects in adults with amnestic MCI.[105,106]

Cogstate One Card Learning.[57] The OCL is a test of short-term memory that is sensitive to early changes associated with AD.[107,108] In this task, a playing card is presented in the center of the iPad and starting with Trial 2, the participant must decide whether or not the same card was seen before in this task by responding with a finger touch. Administration time is 5 minutes. Performance on DET and IDN will be used to adjust OCL performance for simple and choice reaction time.

Cogstate GMLT-Delayed Recall. Free recall of the previously learned hidden path will be assessed following a delay of approximately 15 minutes.

11.3 Behavioral and Functional Evaluations

11.3.1 Clinical Dementia Rating Scale – Sum of Boxes (described above)

11.3.2 ADCS-Activities of Daily Living-MCI

The ADCS-ADL-MCI scale is a questionnaire assessing ability to perform everyday tasks, and is used to detect functional decline in adults with MCI.[109] It was adapted from an inventory developed by the ADCS to assess functional performance in subjects with AD.[110] In a structured

interview, study partners are queried as to whether participants attempted each item in the inventory during the prior 4 weeks and at what 'level' of performance. The questions focus predominantly on instrumental activities of daily living (e.g. shopping, preparing meals, using household appliances, keeping appointments, reading).

11.3.3 Behavior Rating Inventory of Executive Function – Adult Version

The BRIEF-A is a standardized and validated questionnaire that captures subjective report of an adult's executive function or self-regulation in an everyday environment. The questionnaire can be administered to obtain self- or informant-report.

11.3.4 Neuropsychiatric Inventory

The NPI is a well-validated, reliable, multi-item instrument to assess psychopathology in MCI and AD dementia based on the results of an interview with the study partner.[111] The NPI evaluates both the frequency and severity of 10 neuropsychiatric features, including delusions, hallucinations, agitation/aggression, dysphoria, anxiety, euphoria, apathy, disinhibition irritability and lability, and aberrant motor behavior, as well as evaluates sleep and appetite/eating disorders. Frequency assessments range from 1 (occasionally, less than once per week) to 4 (very frequently, once or more per day or continuously). Severity assessments range from 1 (mild) to 3 (severe). The score for each subscale is the product of severity and frequency and the total score is the sum of all subscales.

11.3.5 Cognitive Change Index

The Cognitive Change Index, adapted from Saykin et al.,[112] is a self-report questionnaire listing common experiences (e.g., "recalling conversations a few days later", "remembering names and faces of new people I meet") and participants indicate amount of perceived change over the past 5 years using a Likert scale. Only the first 12 questions that address subjective memory concerns will be administered. The same 12-item subscale is used in the large multicenter ADNI-2 study.

11.3.6 Geriatric Depression Scale

The GDS is a well-validated, self-administered questionnaire used to assess mood in geriatric populations.[113]

11.3.7 SF-36

The Short Form Health Survey is a well-validated, commonly used self-administered survey of health status that includes questions about mood, pain, physical functioning, and overall health.[114]

11.3.8 EQ5D

The EQ5D is a brief questionnaire about health-related quality of life that queries 5 dimensions of everyday experience (mobility, self-care, usual activities, pain/discomfort, anxiety/depression) that are each rated using a 5-point scale (no problems/slight problems/moderate problems/severe problems/extreme problems). The EQ5D is a well-validated instrument that provides a single index for overall health status.[115]

11.3.9 Study Partner Self-Assessment Questionnaire

This self-administered questionnaire assesses the study partner's mood, physical functioning, sleep, and overall health status, and includes the SF-36 (described above) and 2 well-validated brief assessments of sleep quality (Pittsburgh Sleep Quality Index) and duration (Insomnia Severity Index[116]).

11.4 Modified Hachinski

This brief questionnaire, conducted by a clinician, incorporates information regarding medical history, cognitive symptoms and features of stroke, reported by a study partner as well as the neurological examination, and neuroimaging studies.[117]

11.5 Research Satisfaction Survey

A Research Satisfaction Survey will be administered to the participant and study partner to evaluate satisfaction with the study. The survey may reveal specific aspects of the study that participants dislike, which can inform efforts to improve their experiences when participating in future studies. Past studies show that participant input and feedback is important for retention.[118,119]

11.6 Treatment Blinding Questionnaire

Following the Month 12 and 18 clinic assessments, the site PI and Raters will complete a Treatment Blinding Questionnaire to document knowledge of intervention group assignment per participant.

11.7 Heart Rate Monitor

A HR monitor that includes a watch-like device and a chest strap will be provided to all participants to wear during exercise, which will permit HR monitoring to assess effort and exercise intensity.

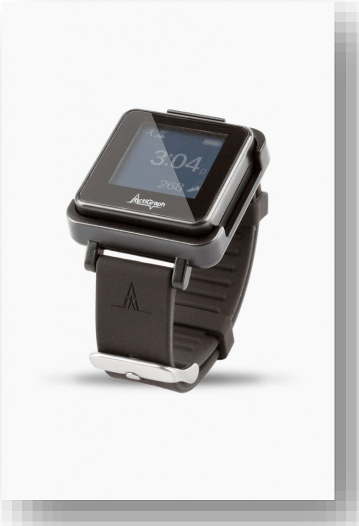

11.8 ActiGraph Link

The ActiGraph Link is a validated 3-axis accelerometer and watch-like device (ActiGraph™) with a low profile case and high-resolution liquid crystal display window (see picture on right). The device contains a gyroscope, magnetometer, and secondary accelerometer to record movement, rotation, and body position data. The ActiGraph Link will be configured to display only the time, not activity information.

11.9 Instruments Administered by Trainers at the YMCA

11.9.1 Rating of Perceived Exertion

The RPE modified Borg scale is a validated and accepted supplemental method for assessing exercise intensity in older adults. The rating scale ranges from 1 to 10 (1 =very mild, to 10 =very, very hard), and participants are trained to self-assess their cardiorespiratory effort using this tool.

11.9.2 Adverse Event Checklist

The AE Checklist will be administered once per week by the Trainer prior to a supervised exercise session. The checklist will query change in health or physical status in the last week, and will include questions to identify SAEs (death, life-threatening, hospitalization, disability or permanent damage).

12.0 STUDY–SPECIFIC PROCEDURES

12.1 Safety Assessments

Physical and neurological examinations will occur every 6 months while participants are enrolled in the study. An MRI will be conducted during screening and repeated at Month 12 for safety assessment and study outcomes. Participants receiving an LP will receive a follow-up telephone call 24 hours later from the clinic to confirm the participant's well-being and to query AEs, which will be reported to the ADCS DSMB. Safety reports will be prepared by the Medical and Safety Core and submitted to the DSMB for periodic review.

Participants will exercise at YMCA facilities staffed by trained personnel who ensure the safety of all YMCA members. Twice per week, a study-certified YMCA Trainer will supervise the participant while exercising to ensure safe practices are consistently used.

12.1.1 Physical and Neurological Examination

A brief physical examination will be performed by a medically qualified professional every 6 months. A review of the major body systems will be performed and vital signs will be assessed. A brief neurological examination will include assessment of motor strength, sensory, deep tendon, tremor, cerebellar, cranial, and mental status.

12.1.2 Electrocardiogram

A standard 12-lead resting ECG will be performed during screening. The ECG report will be reviewed, signed, and dated by the investigator. Those with clinically significant ECG findings will be referred for follow-up as deemed appropriate by the investigator, and may be excluded from the study.

12.1.3 Clinical Laboratory Evaluations

All routine laboratory samples will be analyzed by a central laboratory, which will provide a procedures manual and supplies. Lab reports will be reviewed, signed and dated by the Study Clinician. If a value is outside of the laboratory’s normal range, the Study Clinician will indicate if it is clinically significant. If clinically significant, lab tests may need to be repeated and/or follow-up with the participant’s PCP may be required.

Clinical laboratory assessments include:

- Chemistry Panel (Screening II, Month 12)
- Fasting glucose and hemoglobin A1c
- TSH (Screening II)
- CBC | Differential (Screening II, Month 12)
- Vitamin B12 (Screening II); Note: HCY & MMA reflex tests will be run only if the B12 level is ≤211 pg/ml; additional blood draw will not be required for reflex testing
- PT, PTT Coagulation Panel (Screening II, Month 12)

12.2 Biofluids

Biofluid collection schedule, quantities, and assessments are summarized in the table below.

Biofluids	Screening II (Visit 1)	Baseline (Visit 2)	Month 6 (Visit 3)	Month 12 (Visit 4)	Month 18 (Visit 5)
**ApoE Genotyping for Randomization**	X 20ml EDTA blood				
**Banked Plasma: Aβ and other AD biomarkers**		X 30ml EDTA blood	X 30ml EDTA blood	X 30ml EDTA blood	X 30ml EDTA blood
**Banked PBMCs**		X 10ml EDTA blood	X 10ml EDTA blood	X 10ml EDTA blood	X 10ml EDTA blood
**Banked CSF: total tau, Aβ42, Aβ40, and phosphor-tau181, isoprostane 8,12-iso-iPF2alpha-VI**		X 20ml CSF		X 20ml CSF	

12.2.1 Genetic Samples, Storage and Future Use

DNA will be extracted from participant blood samples to determine ApoE genotypes, which will be examined with regard to their roles on cognitive and biomarker response to the interventions. Participants will be asked to consent to optional DNA banking for future research studies. ApoE genotyping will be performed and DNA will be banked using established ADCS Biomarker Core protocols.

12.2.2 Blood Collection for Biomarker Analyses and Banking

Blood will be collected to extract plasma and peripheral blood mononuclear cells (PBMCs) in the morning after a minimum 10-hour overnight fast, and shipped to the ADCS Biomarker Core at UCSD for banking and analysis.

12.2.3 CSF Collection for Biomarker Analyses and Banking

It is expected that most participants will receive LPs pre- and post-treatment. Coagulation and platelets will be examined in blood before an LP is performed. All CSF samples will be collected after an overnight fast. Participants who are taking anticoagulants, warfarin (Coumadin) and dabigatran (Pradaxa) should not be screened for an LP, as these are prohibited medications. Based on clinician judgment and depending on the clinical indication, it may be suitable to discontinue participants from their anti-platelet agent (e.g., aspirin, Plavix, NSAIDs) for 5-7 days prior to the LP and until at least 24 hours after the procedure. It is not required that participants be discontinued from their anti-platelet agent in order to screen and enroll in EXERT.

The baseline LP must be conducted prior to randomization. At Month 12, the LP should be performed after the MRI or at least 72 hours before the MRI, and at least 24 hours after the last bout of exercise.

A total of 20 ml of CSF will be collected during each LP. To clear any blood from minor trauma associated with needle insertion, the first 1-2 mL of CSF are discarded (or more if needed). Collected CSF will be aliquoted into sterile microtubes. Approximately 2 mL of CSF or volume per local laboratory requirements will be sent at ambient temperature to the local laboratory for protein, glucose and cell count. The remaining CSF will be shipped to the ADCS Biomarker Core at UCSD for storage and analysis.

12.3 Magnetic Resonance Imaging

12.3.1 Site Qualification

Structural MRI. All sMRI procedures will be overseen by the ADCS Imaging Core. Each site's scanner will have an identifying number, which will be appended to the image header of all MRI data. Each scanner will be subjected to a qualifying process that includes an evaluation for excessive vibration or other image artifact revealed through submitted magnetic resonance (MR) images obtained through a standard protocol of an American College of Radiology phantom. In addition, 3D T1 MPRAGE or IR-FSPGR images of a human brain obtained through a standardized imaging protocol of a volunteer at each site will be submitted to the ADCS Imaging Core for automated segmentation, and the resulting segmentations will be examined by ADCS Imaging Core for anatomical accuracy. Once the phantom and volunteer scan pass quality control by the ADCS Imaging Core, the scanner will be considered certified for EXERT. Because longitudinal analysis of brain volumetry will be used in this study, sites will be required to use the same scanner for both scans (Baseline, Month 12) of a particular participant.

Functional MRI. Arterial Spin Labeling (ASL) is a contrast-free, resting-state fMRI approach to quantify regional blood flow in the brain. Sites that are able to perform ASL will be asked to work with the Imaging Core and collaborators at Wake Forest to establish a qualified ASL protocol, which can be appended to the sMRI protocol. ASL will be evaluated and approved centrally in a manner similar to the sMRI qualification procedures described above. Unlike sMRI qualification for safety and volumetric outcomes, ASL qualification will not be required for initiating study procedures.

12.3.2 Data Acquisition

The same MRI protocols will be administered at screening and Month 12, which will include a localizer scan, followed by a high-resolution 3D T1 structural series (MPRAGE or IR-SPGR), a T2-weighted series (FLAIR), a diffusion weighted scan, and a gradient recalled echo scan. Sites with access to approved 3.0 Tesla (3T) scanners will be asked to include a resting-state ASL fMRI series that will be appended to the standard ADCS protocol.

12.3.3 Data Management and Quality Control

Images will be checked for image quality and adherence to scanning protocols. 3D T1-weighted datasets passing quality checks will be corrected for spatial distortion and for intensity variation. Baseline and follow-up datasets for each participant will be spatially registered to one another using rigid-body registration followed by nonlinear registration and neuroanatomic parcellation to quantify whole-brain and subregional volumetric change on a participant-by-participant basis.

Sites will use a scanner expected to be available for the duration of the study. Scanners used successfully in prior ADCS studies, or are likely to have convenience of scheduling and participant experience, are preferred, regardless of field strength for sites collecting only sMRI data. For approved sites that will also collect fMRI, 3T machines must be used. Scanners must pass the study's qualification procedures, and participants must be scanned by the same scanner at Baseline and Month 12. The analysis procedure will include corrections for gradient nonlinearities and intensity non-uniformity.[120-122]

Sites will be required to procure a clinical read of each participant's MRI to confirm eligibility based on image-derived inclusion and exclusion criteria. Inconclusive findings will be subject to review by the ADCS Imaging Core. All imaging data will be de-identified through a numerical coding system and submitted to the ADCS EDC.

12.4 400 m Walk Test

The Walk Test is a low-cost and safe means of assessing walking speed and endurance that correlates with peak oxygen consumption in older adults,[123] and provides community-based translational relevance. The timed walk covers 10 laps totaling 400 m. The will be administered by trained personnel using a standardized protocol that has been validated in a large trials of older adults (e.g., LIFE).[78,86] In EXERT, participants will not undergo exercise stress testing. The American Heart Association and the ACSM joint position statement advised that "apparently healthy persons of all ages and asymptomatic persons at increased risk may participate in moderate-intensity exercise without first undergoing a medical examination or a medically supervised, symptom-limited exercise test".[124] Exercise stress testing, although clinically useful to assess symptomatic coronary heart disease, does not add predictive value for asymptomatic community-dwelling older adults before the start of a supervised and individualized program, particularly when exercise intensity and duration are gradually ramped up over time.[11] Older persons have a high prevalence of ECG abnormalities,[125] which diminish the diagnostic accuracy of treadmill exercise testing.[126] Participants with potential cardiac contraindications for participation in either intervention will be excluded during screening.

12.5 Actigraphy Recordings

Accelerometry data will be collected over a 10-day period at Baseline, and at Months 6, 12, and 18. Participants will continuously wear the ActiGraph Link, a watch-like device, for the entire recording period – even while showering and bathing. It is also important that participants wear the device while sleeping.

During Screening II after the MRI and when the majority of eligibility requirements have been confirmed, a clinic staff member will initialize the device and place it on the participant's non-dominant wrist to start the 10-day recording period. Ten days later (at a minimum), the device should be retrieved from the participant during a baseline visit (preferable) or by mail (with a reminder call on Day 10), and the data uploaded using the provided ActiGraph hub. The baseline actigraphy recording is to be completed prior to randomization (highly preferable), but most certainly before intervention initiation at the YMCA.

For the Month 6 and 12 assessments, the ActiGraph Link should once again be placed on the participant's non-dominant wrist during the clinic visit to initiate the recording. Participants should be reminded to continuously wear the device, even in the shower and while sleeping, for the entire 10-day period. The device should be retrieved from the participant 10 days later (at a minimum) by return mail, with a reminder call on Day 10, and the data uploaded using the provided ActiGraph hub.

For the Month 18 assessment, the device should be sent to the participant 14 days before the visit, with instructions to place on the non-dominant wrist and wear continuously until their scheduled clinic appointment. A staff member should call the participant to confirm that the ActiGraph Link was received and is in place at the start of the recording period. At the Month 18 visit, the device will be removed from the participant's wrist and the data uploaded using the ActiGraph hub.

13.0 PERSONNEL REQUIREMENTS

Personnel roles and requirements to carry out the protocol are listed below. Additional details are provided in the procedures manual.

13.1 Clinic Personnel

At a minimum, 4 clinic staff members (Site PI/Study Clinician, Study Coordinator/Clinic YMCA Liaison, Rater 1, Rater 2) and will be required to carry out the protocol at each site. Additional functions are outlined below that may be covered one staff member at some sites, or multiple staff at other sites.

- **Site Protocol Principal Investigator:** The Protocol PI is responsible for the overall conduct of the study at the site. The PI will perform or supervise clinical evaluation of all participants and ensure protocol adherence. The PI will supervise project personnel and

ensure that the Raters maintain a high level of skill and accuracy in conducting assessments. If the PI does not also serve as the Study Clinician (unblinded), the PI may complete certain rating scales.

- **Study Clinician:** The Study Clinician will be responsible for conducting and supervising the medical evaluation (physical and neurological examinations), ensuring medical eligibility for the study, reviewing AEs, interpreting laboratory results, and supervising study-related clinical care provided to the participant. This person will also perform, or supervise trained medical staff to perform, the LPs. The Study Clinician may be a physician, or if consistent with local practice and regulations, a nurse practitioner or physician's assistant. The Study Clinician will be reviewing AEs and thus will be unblinded to intervention group assignment.

- **Study Coordinator:** This person will be responsible for managing the day-to-day conduct of the trial. Duties may include tracking recruitment, ensuring accurate administration of all instruments at the site, maintaining CRFs, processing of laboratory samples, serving as liaison with the ADCS clinical monitor, coordinating clinic visits, administering cognitive assessments, and data entry of outcomes other than those associated with intervention adherence. If the Study Coordinator also serves as the Clinic YMCA Liaison (unblinded), this person may not be involved in outcomes collection or data entry at any time during the study.

- **Clinic YMCA Liaison:** This person will serve as the primary liaison between the clinic and the participating local YMCA to facilitate bi-directional information exchange regarding study and participant progress, and to assist in resolving participant issues as they arise. Thus, this person will be unblinded to intervention group assignment. Duties will include monthly visits with the YMCA study team; monthly telephone calls to participants to query progress, AEs, and new medications; and attendance on twice per month calls with the Intervention Oversight Team. This person must not have access to cognitive or functional data, and thus may not serve as a Rater or be involved in data entry of these outcomes at any time during the study.

- **Raters 1 and 2:** Raters will be blinded to intervention group assignment, and will be responsible for administering the cognitive and functional assessments, and maintaining all appropriate certifications. The same individual should not administer both the ADAS-Cog13 and the CDR. The Raters must be certified to administer the 400 m Walk Test if the Study Coordinator is unblinded and thus unable to collect study outcomes.

13.2 YMCA Study Team (see **Section 6.2.1**)

14.0 ADVERSE EVENTS

Potential AEs for study-related activities and interventions are described to each participant by trained clinic staff during the informed consent process. Participants are instructed to report the occurrence of an AE to the Clinic YMCA Liaison or Study Clinician. Participants will also be instructed to report AEs to the YMCA Trainers during supervised exercise sessions.

Trainers will complete the AE Checklist with participants once per week during a supervised exercise session. The AE Checklist will be faxed to the clinic to permit review and data entry of Study Clinician-verified AEs within 24 hours of reporting, and AE medical management as needed. AEs will also be queried at each clinic assessment visit, and during the monthly telephone call by the Clinic YMCA Liaison.

14.1 Definition

An AE is defined as per the Code of Federal Regulation Title 21 Part 312, http://www.accessdata.fda.gov/scripts/cdrh/cfdocs/cfcfr/cfrsearch.cfm?fr=312.32, and refers to any health-related unfavorable or unintended medical event that occurs after the informed consent is signed. An AE includes but is not limited to: (1) worsening or change in nature, severity, or frequency of conditions or symptoms present at the start of the study; (2) participant deterioration due to primary illness; (3) intercurrent illness; and (4) physical incapacity to carry out the intervention. An abnormal laboratory value will only be reported as an AE if the investigator considers it to be an AE, or if it leads to the discontinuation or termination of the intervention.

Minor AEs are defined as conditions that may be unpleasant and bothersome to the participant such as sore muscles, but do not require intervention discontinuation. Examples of minor AEs include but are not limited to the following: anxiety, fatigue, decreased appetite, insomnia, dizziness, muscle or joint stiffness, muscle strain or soreness, ankle or knee pain, foot pain, and other minor symptoms that may have restricted the participant’s usual activities for at least ½ day like a head cold, flu or allergy problems. Minor AEs should be reported on an annual basis to the site IRB.

The investigator should attempt to establish a diagnosis of the event based on signs, symptoms, and or other clinical information. In such cases, the diagnosis should be documented as the AE and not the individual signs or symptoms. Symptoms and conditions present at the beginning of the study will be characterized, so that AEs can be defined as any new symptom, or any increase in frequency or severity of an existing symptom. Adverse events should be described with medical terminology so that the event can be matched against a medical coding dictionary, such as MedDRA.

14.2 Follow-Up of AEs

The Study Clinician will be responsible for review of all AEs. Following questioning and evaluation, all AEs, whether determined to be related or unrelated to the intervention by the Study Clinician, must be documented in the participant's medical record in accordance with the investigator's normal clinical practice, and on the AE Case Report Form (e-CRF). Adverse events will be rated as mild, moderate or severe, and for duration, severity, seriousness and causal relationship to the intervention. This will also pertain to abnormal laboratory values deemed clinically significant by the Study Clinician.

The Study Clinician is obliged to follow participants with AEs until the events have subsided, the conditions are considered medically stable, or the participants are no longer available for follow up. Participants who discontinue the study due to adverse experiences will be treated and followed according to established medical practice. All AEs will be reported to the ADCS DSMB.

14.3 Reporting of Unanticipated Problems, AEs, Protocol Deviations

Any unanticipated problems, serious and unexpected AEs, deviations or protocol changes will be promptly reported by the PI or a designated member of the research team to the site IRB, the ADCS DSMB, and the sponsor (NIA) if appropriate.

15.0 SERIOUS ADVERSE EVENTS

15.1 Definition

A SAE is defined as per the Code of Federal Regulation Title 21 Part 312, http://www.accessdata.fda.gov/scripts/cdrh/cfdocs/cfcfr/cfrsearch.cfm?fr=312.32, and includes: death, a life-threatening adverse experience, inpatient hospitalization or prolongation of existing hospitalization, a persistent or significant disability/incapacity, or a clinically significant laboratory or clinical test result. Important medical events that may not result in death, be life-threatening, or require hospitalization may be considered as SAEs if they have the potential to jeopardize participant safety or to require medical or surgical intervention to prevent a more serious outcome listed above, such as an injurious fall resulting in a fracture that occurred while completing one of the exercise interventions.

15.2 Reporting SAEs

Any SAE due to any cause that occurs during the course of the investigation (i.e., anytime after informed consent, regardless of study intervention exposure) must be reported to the clinic and the ADCS within 24 hours of learning of the event. All SAEs will be reported to the DSMB, with the blind maintained and in summary format (i.e., Group A, Group B).

16.0 DATA AND SAFETY MONITORING BOARD

The ADCS DSMB reviews the safety of all participants enrolled in trials on an ongoing basis. The initial task of the DSMB will be to review the protocol to identify any necessary modifications. If modifications are necessary, revisions will be reviewed by the DSMB prior to its recommendation on initiation of the project. The DSMB, based on its review of the protocol, will work with the Medical and Safety Core personnel to identify the study-specific data parameters and format of the information to be regularly reported. The DSMB will initially be provided with data blinded to treatment status, but they may request unblinded data if there is a safety concern. The DSMB and NIA representative will meet in person or by conference call on a quarterly basis.

Additionally, the DSMB will be informed of the occurrence of any SAEs within 7 days of being reported to the ADCS. The DSMB may at any time request additional information from the ADCS.

Based on the review of safety data, the DSMB will make recommendations regarding the conduct of the study. These may include amending safety monitoring procedures, modifying the protocol or consent, terminating the study or continuing the study as designed. Using the ADCS Safety Review Process and the DSMB, there is substantial oversight and case review to alert the investigators, in a timely manner, to any safety issues that may arise. For further details please refer to the DSMB charter.

17.0 RECORDING AND COLLECTION OF DATA

17.1 Case Report Form

The site PI or designee will record all data collected (either written or electronic record of data). Written or electronic data of record must be entered on the electronic version of the CRF (e-CRF) provided for that purpose. In some instances no prior written or electronic record of data may exist and data recorded directly on the e-CRF is considered source data. The site will be suitably trained on the use of the e-CRF and appropriate site personnel will be authorized to provide electronic signatures. The PI is responsible to verify the integrity of the data and acknowledge as such by signature.

All site entries will be made in a secured web site and the PI or designee will review the record for completeness. If corrections are necessary to the e-CRFs, the PI or designee will update the e-CRF and document the reason for change.

Completed e-CRFs will be submitted according to ADCS protocols, and reviewed by the ADCS to determine their acceptability. If necessary, data correction requests will be generated for resolution by the study site.

17.2 Audio / Visual Recording

It is the site PI's responsibility to ensure that any audio or visual recording conducted as part of the protocol are completed according to locally applicable laws and regulations.

17.3 Study Files and Patient Source Documents

Participant confidentiality is strictly held in trust by the investigators, research staff, and the ADCS and/or sponsoring institution and their agents. This confidentiality is extended to cover testing of biological samples and genetic tests, and participant clinical information.

The study protocol, documentation, data and all other information generated will be held in strict confidence. No information concerning the study or the data will be released to any unauthorized third party, without prior written approval of the sponsoring institution. Authorized representatives of the sponsoring institution may inspect all required documents and records maintained by the investigator, including but not limited to, medical records (office, clinic or hospital) and pharmacy records for participants. The study site will permit access to such records. Any data, specimens, forms, reports, video/audio recordings, and other records that leave the site will be identified only by a participant identification number (PID) to maintain confidentiality. All records will be kept in a locked file cabinet. All computer data entry will include only PIDs. Information will not be released without written permission of the participant, except as necessary for monitoring by the IRB, the Intervention Oversight Team, the Food and Drug Administration (FDA), the NIA, and the Office for Human Research Protections (OHRP).

18.0 ETHICS AND REGULATORY CONSIDERATIONS

18.1 Good Clinical Practice

This study will be conducted in accordance with Good Clinical Practice (GCP) guidelines, as defined by the International Conference on Harmonisation (ICH) Guideline, Topic E6, the United States Code of Federal Regulations, Title 21, Part 50 (21CFR50) – Protection of Human Subjects and Part 56 – IRBs, HIPAA, State and Federal regulations and all other applicable local regulatory requirements and laws.

Study personnel involved in conducting this study will be qualified by education, training and experience to perform their respective task(s) in accordance with GCP.

No study document shall be destroyed without prior written agreement between the ADCS and the investigator. Should the investigator wish to assign study records to another party or move them to another location, he/she may do so only with the prior written consent of the ADCS.

18.2 Institutional Review Board

Each participating institution must provide for the review and approval of this protocol and the associated informed consent documents and recruitment material by an appropriate IRB registered with the OHRP. Any amendments to the protocol or consent materials must also be approved before they are placed into use. In the United States, only institutions holding a current US Federal-wide Assurance issued by OHRP may participate. Refer to: http://www.hhs.gov/ohrp/assurances/.

The investigator must obtain approval from the IRB for all subsequent ADCS protocol amendments and, when warranted, changes to the informed consent document. Protocol and informed consent form amendments can be made only with the prior approval of the ADCS. The investigator may not implement any protocol deviation without prior notification to the ADCS and prior review and documented approval of the IRB, except where necessary to eliminate an immediate hazard to study participants, or when change(s) involve only logistical or administrative aspects of the trial (ICH 4.5.4). The investigator shall notify the IRB of deviations from the protocol or SAEs occurring at the site, in accordance with local procedures.

18.3 Informed Consent and HIPAA Compliance

In obtaining and documenting informed consent, the investigator should comply with the applicable regulatory requirement(s), and should adhere to GCP and to the ethical principles that have their origin in the Declaration of Helsinki. Prior to the beginning of the trial, the investigator should have IRB approval of the written ICF and any other written information to be provided to participants. Participants, their relatives, guardians, or authorized representatives and study partners will be given ample opportunity to inquire about the details of the study. Prior to a participant's participation in the trial, the written ICF should be signed and personally dated by the participant and by the person who conducted the informed consent discussion. Participants should be provided a copy of the signed ICF.

The informed consent will not only cover consent for the trial itself, but also for the genetic research, biomarker studies, biological sample storage and brain imaging. The consent for storage will include consent to access stored data, biological samples, and imaging data for secondary analyses. Consent forms will specify that DNA and biomarker samples are for research purposes only; the tests on the DNA and biomarker samples are not diagnostic in nature and participants will never receive results. MRI scan findings of clinical significance, determined by the site radiologist, can be shared with participants per Study Clinician discretion.

Consent forms will be developed by the ADCS Regulatory Affairs in collaboration with the PDs. The sample consent form provided to sites will include all of the required elements of informed consent required by the FDA. The sample consent form will be sent to participating sites where it should be tailored to include site-specific information to meet local IRB regulations. Each site PI, under the guidance of the local IRB, is responsible for ensuring that all applicable state laws are met with regards to judgment of competency and the consent form process. Each

participating site must submit a letter of IRB-approval and the approved consent form to Regulatory Affairs at the ADCS, along with other required regulatory records and essential documents in order to be approved to enroll participants into EXERT. During the first monitoring visit following participant enrollment, the ADCS clinical monitors will review the consent forms and verify that the proper signatures have been obtained.

18.3.1 Participant Confidentiality | HIPAA

Information about study participants will be kept confidential and managed according to the requirements of HIPAA. HIPAA regulations require a signed HIPAA Authorization informing the participant of the following:

- What protected health information (PHI) will be collected from participants in this study
- Who will have access to that information and why
- Who will use or disclose that information
- The rights of a research participant to revoke their authorization for use of their PHI

In the event that a participant revokes authorization to collect or use PHI, the investigator, by regulation, retains the ability to use all information collected prior to the revocation of participant authorization. Each site PI, under the guidance of the local IRB, is responsible for ensuring that all applicable HIPAA regulations and State laws are met.

18.4 Genetic Research and Storage of Genetic Material

The DNA is banked in locked freezers in the ADCS Biomarker laboratory at UCSD. Sample tubes are bar-coded and linked to PID only and banked without personal identifiers. The presence of the sample is recorded into a computerized inventory database that is managed by ADCS Informatics and is encrypted and password-protected.

Only DNA from consenting participants will be banked and used to facilitate future research on aging and dementia, particularly in the discovery of genetic polymorphisms that may influence risk of developing AD. Collection of DNA will permit ADCS investigators to probe candidate genetic polymorphisms as predictors of outcome in future studies. The samples will be stored by the ADCS as long as funding is available from the NIH. If funding should lapse completely, the UCSD ADRC will provide responsible custodianship of the ADCS biospecimen bank.

18.5 Storage of Biospecimen Samples

All biospecimens banked for future AD biomarker research will be shipped to and stored by the ADCS Biomarker Core at UCSD. Sample tubes are bar-coded and linked to PID only and banked without personal identifiers. The presence of the sample is recorded into a computerized inventory database that is managed by ADCS Informatics and is encrypted and password-protected.

18.6 MRI Data Storage

MRI scans will be labeled according to each site's imaging machine capabilities using PID and scanner specific series descriptions as detailed in the Technical Manual. All imaging data will be de-identified using participant identifiers as detailed in the Technical Manual and checked by the ADCS Imaging Core to confirm the absence of participant identifying information.

18.7 Inclusion of Children as Participants in Research Involving Human Subjects

Children will not be included.

18.8 Study Monitoring

The ADCS clinical monitors are responsible for inspecting the e-CRFs and source documents at regular intervals throughout the study to verify adherence to the protocol, completeness and accuracy of the data, and adherence to local regulations on the conduct of clinical research. An unblinded monitor will inspect documents pertaining to randomization, intervention implementation, and AEs. A blinded monitor will inspect all other source documents.

The monitoring visits must be conducted according to the applicable ICH and GCP guidelines to ensure protocol adherence, quality of data, drug accountability, compliance with regulatory requirements and continued adequacy of the investigational site and its facilities. The site investigator will cooperate in the monitoring process by ensuring the availability of the e-CRFs, source and other necessary documents at the time of the monitoring visits. The site PI will promptly address any matters brought to his/her attention by the monitor, and may be asked to meet in-person with the site monitor during monitoring visits.

19.0 AUDIT

In accordance with ICH GCP, representatives of the ADCS and/or sponsor and/or regulatory agency may select this study for audit. The site PI and study staff are responsible for maintaining the site master file containing all study-related regulatory documentation as outlined by ADCS Regulatory Affairs that will be suitable for inspection at any time by ADCS, sponsor, its designees, and/or regulatory agencies. Inspection of site facilities (e.g., pharmacy, laboratories, YMCAs) to evaluate the trial conduct and compliance with the protocol may also occur.

20.0 RECORD RETENTION

Essential documents and study records must be retained for a minimum of 7 years following primary publication of study results. The ADCS will notify sites when retention of such documents is no longer required.

21.0 PUBLICATION POLICY

The results of this study will be published. To coordinate dissemination of data from this study, a publication committee will be formed. The committee will consist of the PDs, the Protocol Committee, interested site PIs, and appropriate ADCS personnel. The publication committee will solicit input and assistance from other investigators as appropriate and adhere to all ADCS Publications Policies.

22.0 SHARING OF FINAL RESEARCH DATA

Data from this research will be shared with other researchers pursuant to the 02/26/2003 “Final NIH Statement on Sharing Research Data.” The ADCS grant contains a data sharing policy consistent with the goals of the NIH that also respects the rights of commercial partners. The NIH policy can be found at: **http://grants.nih.gov/grants/guide/notice-files/NOT-OD-03-032.html**.

The NIH endorses the sharing of final research data to serve these and other important scientific goals. To protect participants’ rights and confidentiality, identifiers will be removed from the data before they are shared.

23.0 LITERATURE CITED

1. Broe GA, Creasey H, Jorm AF, Bennett HP, Casey B, Waite LM, et al. Health habits and risk of cognitive impairment and dementia in old age: a prospective study on the effects of exercise, smoking and alcohol consumption. *Aust N Z J Public Health.* 1998;22(5):621-623.
2. Rolland Y, Abellan van Kan G, Vellas B. Healthy brain aging: role of exercise and physical activity. *Clin Geriatr Med.* 2010;26(1):75-87.
3. Laurin D, Verreault R, Lindsay J, MacPherson K, Rockwood K. Physical activity and risk of cognitive impairment and dementia in elderly persons. *Arch Neurol.* 2001;58:498-504.
4. Yoshitake T, Kiyohara Y, Kato I, Ohmura T, Iwamoto H, Nakayama K, et al. Incidence and risk factors of vascular dementia and Alzheimer's disease in a defined elderly Japanese population: the Hisayama Study. *Neurology.* 1995;45(6):1161-1168.
5. Podewils LJ, Guallar E, Kuller LH, Fried LP, Lopez OL, Carlson M, et al. Physical activity, APOE genotype, and dementia risk: findings from the Cardiovascular Health Cognition Study. *Am J Epidemiol.* 2005;161(7):639-651.
6. Albert MS, Jones K, Savage CR, Berkman L, Seeman T, Blazer D, et al. Predictors of cognitive change in older persons: MacArthur studies of successful aging. *Psychol Aging.* 1995;10(4):578-589.
7. Geda YE, Roberts RO, Knopman DS, Christianson TJ, Pankratz VS, Ivnik RJ, et al. Physical exercise, aging, and mild cognitive impairment: a population-based study. *Arch Neurol.* 2010;67(1):80-86.
8. Sumic A, Michael YL, Carlson NE, Howieson DB, Kaye JA. Physical activity and the risk of dementia in oldest old. *J Aging Health.* 2007;19(2):242-259.
9. Smith PJ, Blumenthal JA, Hoffman BM, Cooper H, Strauman TA, Welsh-Bohmer K, et al. Aerobic exercise and neurocognitive performance: a meta-analytic review of randomized controlled trials. *Psychosom Med.* 2010;72(3):239-252.
10. Colcombe SJ, Erickson KI, Scalf PE, Kim JS, Prakash R, McAuley E, et al. Aerobic exercise training increases brain volume in aging humans. *J Gerontol A Biol Sci Med Sci.* 2006;61(11):1166-1170.
11. Voss MW, Prakash RS, Erickson KI, Basak C, Chaddock L, Kim JS, et al. Plasticity of brain networks in a randomized intervention trial of exercise training in older adults. *Front Aging Neurosci.* 2010;2.
12. Burdette JH, Laurienti PJ, Espeland MA, Morgan A, Telesford Q, Vechlekar CD, et al. Using network science to evaluate exercise-associated brain changes in older adults. *Front Aging Neurosci.* 2010;2:23.
13. Colcombe S, Erickson K, Raz N, Webb A, Cohen N, McAuley E, et al. Aerobic fitness reduces brain tissue loss in aging humans. *J of Gerontol: Med Sci.* 2003;58A(2):176-180.
14. Burns JM, Cronk BB, Anderson HS, Donnelly JE, Thomas GP, Harsha A, et al. Cardiorespiratory fitness and brain atrophy in early Alzheimer disease. *Neurology.* 2008;71(3):210-216.
15. Makizako H, Shimada H, Doi T, Yoshida D, Ito K, Kato T, et al. The association between decline in physical functioning and atrophy of medial temporal areas in community-dwelling older adults with amnestic and nonamnestic mild cognitive impairment. *Arch Phys Med Rehabil.* 2011;92(12):1992-1999.

16. Baker LD, Frank LL, Foster-Schubert K, Green PS, Wilkinson CW, McTiernan A, et al. Effects of aerobic exercise on mild cognitive impairment: A controlled trial. *Arch Neurol.* 2010;67(1):1-9.
17. Lautenschlager NT, Cox KL, Flicker L, Foster JK, van Bockxmeer FM, Xiao J, et al. Effect of physical activity on cognitive function in older adults at risk for Alzheimer disease: a randomized trial. *Jama.* 2008;300(9):1027-1037.
18. Baker LD, Skinner J, Saucedo-Hernandez H, Sink KM, Wilson V, Craft S, et al. Therapeutic effects of high intensity aerobic exercise in adults with two strikes against them: MCI and prediabetes. In: Wake Forest University Health Sciences; submitted.
19. Garber CE, Blissmer B, Deschenes MR, Franklin BA, Lamonte MJ, Lee IM, et al. American College of Sports Medicine position stand. Quantity and quality of exercise for developing and maintaining cardiorespiratory, musculoskeletal, and neuromotor fitness in apparently healthy adults: guidance for prescribing exercise. *Med Sci Sports Exerc.* 2011;43(7):1334-1359.
20. Baker LD, Frank LL, Foster-Schubert K, Green PS, Wilkinson CW, McTiernan A, et al. Aerobic exercise improves cognition for older adults with glucose intolerance, a risk factor for Alzheimer's disease. *J Alzheimers Dis.* 2010;22(2):569-579.
21. Tolppanen AM, Solomon A, Kulmala J, Kareholt I, Ngandu T, Rusanen M, et al. Leisure-time physical activity from mid- to late life, body mass index, and risk of dementia. *Alzheimers Dement.* 2014.
22. Liang KY, Mintun MA, Fagan AM, Goate AM, Bugg JM, Holtzman DM, et al. Exercise and Alzheimer's disease biomarkers in cognitively normal older adults. *Ann Neurol.* 2010;68(3):311-318.
23. Pescatello LS. Physical activity, cardiometabolic health and older adults: recent findings. *Sports Med.* 1999;28(5):315-323.
24. Holland D, Brewer JB, Hagler DJ, Fennema-Notestine C, Dale AM. Subregional neuroanatomical change as a biomarker for Alzheimer's disease. *Proc Natl Acad Sci U S A.* 2009;106(49):20954-20959.
25. Jack CR, Jr., Vemuri P, Wiste HJ, Weigand SD, Aisen PS, Trojanowski JQ, et al. Evidence for Ordering of Alzheimer Disease Biomarkers. *Arch Neurol.* 2011.
26. Okonkwo OC, Mielke MM, Griffith HR, Moghekar AR, O'Brien RJ, Shaw LM, et al. Cerebrospinal fluid profiles and prospective course and outcome in patients with amnestic mild cognitive impairment. *Arch Neurol.* 2011;68(1):113-119.
27. Shaw LM, Vanderstichele H, Knapik-Czajka M, Figurski M, Coart E, Blennow K, et al. Qualification of the analytical and clinical performance of CSF biomarker analyses in ADNI. *Acta Neuropathol.* 2011;121(5):597-609.
28. Snider BJ, Fagan AM, Roe C, Shah AR, Grant EA, Xiong C, et al. Cerebrospinal fluid biomarkers and rate of cognitive decline in very mild dementia of the Alzheimer type. *Arch Neurol.* 2009;66(5):638-645.
29. Jack CR, Jr., Knopman DS, Jagust WJ, Shaw LM, Aisen PS, Weiner MW, et al. Hypothetical model of dynamic biomarkers of the Alzheimer's pathological cascade. *Lancet Neurol.* 2010;9(1):119-128.
30. Fagan AM, Head D, Shah AR, Marcus D, Mintun M, Morris JC, et al. Decreased cerebrospinal fluid Abeta(42) correlates with brain atrophy in cognitively normal elderly. *Ann Neurol.* 2009;65(2):176-183.
31. Erickson KI, Voss MW, Prakash RS, Basak C, Szabo A, Chaddock L, et al. Exercise training increases size of hippocampus and improves memory. *Proc Natl Acad Sci U S A.* 2011;108(7):3017-3022.

32. Olson L, Humpel C. Growth factors and cytokines/chemokines as surrogate biomarkers in cerebrospinal fluid and blood for diagnosing Alzheimer's disease and mild cognitive impairment. *Exp Gerontol.* 2010;45(1):41-46.
33. Tarawneh R, D'Angelo G, Macy E, Xiong C, Carter D, Cairns NJ, et al. Visinin-like protein-1: diagnostic and prognostic biomarker in Alzheimer disease. *Ann Neurol.* 2011;70(2):274-285.
34. Li G, Peskind ER, Millard SP, Chi P, Sokal I, Yu CE, et al. Cerebrospinal fluid concentration of brain-derived neurotrophic factor and cognitive function in non-demented subjects. *PLoS One.* 2009;4(5):e5424.
35. Leverenz JB, Watson GS, Shofer J, Zabetian CP, Zhang J, Montine TJ. Cerebrospinal fluid biomarkers and cognitive performance in non-demented patients with Parkinson's disease. *Parkinsonism Relat Disord.* 2011;17(1):61-64.
36. Nichol KE, Poon WW, Parachikova AI, Cribbs DH, Glabe CG, Cotman CW. Exercise alters the immune profile in Tg2576 Alzheimer mice toward a response coincident with improved cognitive performance and decreased amyloid. *J Neuroinflammation.* 2008;5:13.
37. Garcia-Mesa Y, Pareja-Galeano H, Bonet-Costa V, Revilla S, Gomez-Cabrera MC, Gambini J, et al. Physical exercise neuroprotects ovariectomized 3xTg-AD mice through BDNF mechanisms. *Psychoneuroendocrinology.* 2014;45:154-166.
38. Shen L, Kim S, Qi Y, Inlow M, Swaminathan S, Nho K, et al. Identifying Neuroimaging and Proteomic Biomarkers for MCI and AD via the Elastic Net. *Lect Notes Comput Sci.* 2011;7012:27-34.
39. Thambisetty M, Tripaldi R, Riddoch-Contreras J, Hye A, An Y, Campbell J, et al. Proteome-based plasma markers of brain amyloid-beta deposition in non-demented older individuals. *J Alzheimers Dis.* 2010;22(4):1099-1109.
40. Craig-Schapiro R, Perrin RJ, Roe CM, Xiong C, Carter D, Cairns NJ, et al. YKL-40: a novel prognostic fluid biomarker for preclinical Alzheimer's disease. *Biol Psychiatry.* 2010;68(10):903-912.
41. Perrin RJ, Craig-Schapiro R, Malone JP, Shah AR, Gilmore P, Davis AE, et al. Identification and validation of novel cerebrospinal fluid biomarkers for staging early Alzheimer's disease. *PLoS One.* 2011;6(1):e16032.
42. Bartres-Faz D, Junque C, Clemente IC, Lopez-Alomar A, Valveny N, Lopez-Guillen A, et al. Angiotensin I converting enzyme polymorphism in humans with age-associated memory impairment: relationship with cognitive performance. *Neurosci Lett.* 2000;290(3):177-180.
43. Richard F, Berr C, Amant C, Helbecque N, Amouyel P, Alperovitch A. Effect of the angiotensin I-converting enzyme I/D polymorphism on cognitive decline. The EVA Study Group. *Neurobiol Aging.* 2000;21(1):75-80.
44. Bu G. Apolipoprotein E and its receptors in Alzheimer's disease: pathways, pathogenesis and therapy. *Nat Rev Neurosci.* 2009;10(5):333-344.
45. Miners JS, Ashby E, Van Helmond Z, Chalmers KA, Palmer LE, Love S, et al. Angiotensin-converting enzyme (ACE) levels and activity in Alzheimer's disease, and relationship of perivascular ACE-1 to cerebral amyloid angiopathy. *Neuropathol Appl Neurobiol.* 2008;34(2):181-193.
46. Zhang Z, Deng L, Bai F, Shi Y, Yu H, Yuan Y, et al. ACE I/D polymorphism affects cognitive function and gray-matter volume in amnestic mild cognitive impairment. *Behav Brain Res.* 2011;218(1):114-120.
47. Hajjar I, Kritchevsky S, Newman AB, Li R, Yaffe K, Simonsick EM, et al. Renin angiotensin system gene polymorphisms modify angiotensin-converting enzyme

inhibitors' effect on cognitive function: the health, aging and body composition study. *J Am Geriatr Soc.* 2010;58(6):1035-1042.
48. Bour AM, Rasquin SM, Baars L, van Boxtel MP, Visser PJ, Limburg M, et al. The effect of the APOE-epsilon4 allele and ACE-I/D polymorphism on cognition during a two-year follow-up in first-ever stroke patients. *Dement Geriatr Cogn Disord.* 2010;29(6):534-542.
49. Jacobs DM, Thomas RG, Salmon DP, Jin S, Feldman HH, Cotman CW, et al. Development of a novel cognitive composite outcome to assess therapeutic effects of exercise in the EXERT trial for adults with MCI: The ADAS-Cog-Exec. *Alzheimers Dement (N Y).* 2020;6(1):e12059.
50. Colcombe S, Kramer A. Fitness effects on the cognitive function of older adults: a meta-analytic study. *Psychological Science.* 2003;14(2):125-130.
51. Kramer A, Hahn S, Cohen N, Banich M, McAuley E, Harrison C, et al. Ageing, fitness and neurocognitive function. *Nature.* 1999;400(29):418-419.
52. O'Brien JL, O'Keefe KM, LaViolette PS, DeLuca AN, Blacker D, Dickerson BC, et al. Longitudinal fMRI in elderly reveals loss of hippocampal activation with clinical decline. *Neurology.* 2010;74(24):1969-1976.
53. Vemuri P, Wiste HJ, Weigand SD, Shaw LM, Trojanowski JQ, Weiner MW, et al. MRI and CSF biomarkers in normal, MCI, and AD subjects: predicting future clinical change. *Neurology.* 2009;73(4):294-301.
54. Jack CR, Jr., Slomkowski M, Gracon S, Hoover TM, Felmlee JP, Stewart K, et al. MRI as a biomarker of disease progression in a therapeutic trial of milameline for AD. *Neurology.* 2003;60(2):253-260.
55. Holland D, Brewer JB, Hagler DJ, Fennema-Notestine C, Dale AM. Subregional neuroanatomical change as a biomarker for Alzheimer's disease. *Proceedings of the National Academy of Sciences of the United States of America.* 2009;106(49):20954-20959.
56. Spira AP, Gamaldo AA, An Y, Wu MN, Simonsick EM, Bilgel M, et al. Self-reported Sleep and beta-Amyloid Deposition in Community-Dwelling Older Adults. *JAMA neurology.* 2013.
57. Fredrickson J, Maruff P, Woodward M, Moore L, Fredrickson A, Sach J, et al. Evaluation of the usability of a brief computerized cognitive screening test in older people for epidemiological studies. *Neuroepidemiology.* 2010;34(2):65-75.
58. McDowell I, Kristjansson B, Hill GB, Hebert R. Community screening for dementia: the Mini Mental State Exam (MMSE) and Modified Mini-Mental State Exam (3MS) compared. *J Clin Epidemiol.* 1997;50(4):377-383.
59. Yaffe K, Falvey CM, Hoang T. Connections between sleep and cognition in older adults. *Lancet Neurol.* 2014;13(10):1017-1028.
60. Rolls A, Colas D, Adamantidis A, Carter M, Lanre-Amos T, Heller HC, et al. Optogenetic disruption of sleep continuity impairs memory consolidation. *Proc Natl Acad Sci U S A.* 2011;108(32):13305-13310.
61. Mansikkamaki K, Raitanen J, Nygard CH, Heinonen R, Mikkola T, EijaTomas, et al. Sleep quality and aerobic training among menopausal women--a randomized controlled trial. *Maturitas.* 2012;72(4):339-345.
62. Reid KJ, Baron KG, Lu B, Naylor E, Wolfe L, Zee PC. Aerobic exercise improves self-reported sleep and quality of life in older adults with insomnia. *Sleep medicine.* 2010;11(9):934-940.
63. Santos RV, Viana VA, Boscolo RA, Marques VG, Santana MG, Lira FS, et al. Moderate exercise training modulates cytokine profile and sleep in elderly people. *Cytokine.* 2012;60(3):731-735.

64. Buchman AS, Boyle PA, Yu L, Shah RC, Wilson RS, Bennett DA. Total daily physical activity and the risk of AD and cognitive decline in older adults. *Neurology.* 2012;78(17):1323-1329.
65. Garland T, Jr., Schutz H, Chappell MA, Keeney BK, Meek TH, Copes LE, et al. The biological control of voluntary exercise, spontaneous physical activity and daily energy expenditure in relation to obesity: human and rodent perspectives. *The Journal of experimental biology.* 2011;214(Pt 2):206-229.
66. Turner RS. Biomarkers of Alzheimer's disease and mild cognitive impairment: are we there yet? *Experimental neurology.* 2003;183(1):7-10.
67. Grossman M, Farmer J, Leight S, Work M, Moore P, Van Deerlin V, et al. Cerebrospinal fluid profile in frontotemporal dementia and Alzheimer's disease. *Ann Neurol.* 2005;57(5):721-729.
68. Smith JC, Nielson KA, Woodard JL, Seidenberg M, Durgerian S, Hazlett KE, et al. Physical activity reduces hippocampal atrophy in elders at genetic risk for Alzheimer's disease. *Front Aging Neurosci.* 2014;6:61.
69. US Department of Health and Human Services. Physical Activity Guidelines Advisory Committee Report, 2008 [Internet]. In. Washington DC2008.
70. Siscovick DS, Weiss NS, Fletcher RH, Lasky T. The incidence of primary cardiac arrest during vigorous exercise. *N Engl J Med.* 1984;311(14):874-877.
71. Pollock ML, Wilmore JH. *Exercise in health and disease: evaluation and prescription for prevention and rehabilitation.* 2nd ed. Philadelphia: W. B. Saunders; 1990.
72. Pollack ML. Prescribing exercise for fitness and adherence. In: Dishman RK, ed. *Exercise adherence: its impact on public health.* Champaign, IL: Human Kinetics Books; 1998:259-277.
73. Haskell WL, Lee IM, Pate RR, Powell KE, Blair SN, Franklin BA, et al. Physical activity and public health: updated recommendation for adults from the American College of Sports Medicine and the American Heart Association. *Med Sci Sports Exerc.* 2007;39(8):1423-1434.
74. Pollock ML, Carroll JF, Graves JE, Leggett SH, Braith RW, Limacher M, et al. Injuries and adherence to walk/jog and resistance training programs in the elderly. *Med Sci Sports Exerc.* 1991;23(10):1194-1200.
75. Pollock ML, Gettman LR, Milesis CA, Bah MD, Durstine L, Johnson RB. Effects of frequency and duration of training on attrition and incidence of injury. *Med Sci Sports.* 1977;9(1):31-36.
76. Gill TM, DiPietro L, Krumholz HM. Role of exercise stress testing and safety monitoring for older persons starting an exercise program. *Jama.* 2000;284(3):342-349.
77. Fielding RA, Rejeski WJ, Blair S, Church T, Espeland MA, Gill TM, et al. The Lifestyle Interventions and Independence for Elders Study: design and methods. *J Gerontol A Biol Sci Med Sci.* 2011;66(11):1226-1237.
78. Pahor M, Blair SN, Espeland M, Fielding R, Gill TM, Guralnik JM, et al. Effects of a physical activity intervention on measures of physical performance: Results of the lifestyle interventions and independence for Elders Pilot (LIFE-P) study. *J Gerontol A Biol Sci Med Sci.* 2006;61(11):1157-1165.
79. Amariglio RE, Frishe K, Olson LE, Wadsworth LP, Lorius N, Sperling RA, et al. Validation of the Face Name Associative Memory Exam in cognitively normal older individuals. *Journal of clinical and experimental neuropsychology.* 2012;34(6):580-587.
80. Peskind ER, Riekse R, Quinn JF, Kaye J, Clark CM, Farlow MR, et al. Safety and acceptability of the research lumbar puncture. *Alzheimer Dis Assoc Disord.* 2005;19(4):220-225.

81. Armitage P, Berry G. *Statistical methods in medical research.* 3rd ed: Blackwell Scientific Publications; 1994.
82. Nicklas BJ, Brinkley TE. Exercise training as a treatment for chronic inflammation in the elderly. *Exerc Sport Sci Rev.* 2009;37(4):165-170.
83. Williamson JD, Espeland M, Kritchevsky SB, Newman AB, King AC, Pahor M, et al. Changes in cognitive function in a randomized trial of physical activity: results of the lifestyle interventions and independence for elders pilot study. *J Gerontol A Biol Sci Med Sci.* 2009;64(6):688-694.
84. Baker L. Effects of aerobic exercise on cognition for memory-impaired older adults with prediabetes. In. Veterans Affairs Puget Sound Healthcare System: American Diabetes Association; 2010.
85. Nicklas BJ, Hsu FC, Brinkley TJ, Church T, Goodpaster BH, Kritchevsky SB, et al. Exercise training and plasma C-reactive protein and interleukin-6 in elderly people. *J Am Geriatr Soc.* 2008;56(11):2045-2052.
86. Pahor M, Guralnik JM, Ambrosius WT, Blair S, Bonds DE, Church TS, et al. Effect of structured physical activity on prevention of major mobility disability in older adults: the LIFE study randomized clinical trial. *Jama.* 2014;311(23):2387-2396.
87. Mayer CJ, Steinman L, Williams B, Topolski TD, LoGerfo J. Developing a Telephone Assessment of Physical Activity (TAPA) questionnaire for older adults. *Preventing chronic disease.* 2008;5(1):A24.
88. Teng EL, Chui HC. The Modified Mini-Mental State (3MS) examination. *The Journal of clinical psychiatry.* 1987;48(8):314-318.
89. Cahn DA, Salmon DP, Monsch AU, Butters N, Wiederholt WC, Corey-Bloom J, et al. Screening for dementia of the Alzheimer type in the community: The utility of the Clock Drawing Test. *Arch Clin Neuropsychol.* 1996;11(6):529-539.
90. Wechsler D. *WMS-R Wechsler Memory Scale - Revised Manual.* New York: The Psychological Corporation, Harcourt Brace Jovanovich, Inc.; 1987.
91. Rey A. *L'examen clinique en psychologie.* Paris: Presses Universitaires de France; 1964.
92. Rosen WG, Mohs RC, Davis KL. A new rating scale for Alzheimer's disease. *American Journal of Psychiatry.* 1984;141(11):1356-1364.
93. Hamel R, Kohler S, Sistermans N, Koene T, Pijnenburg Y, van der Flier W, et al. The trajectory of cognitive decline in the pre-dementia phase in memory clinic visitors: findings from the 4C-MCI study. *Psychological medicine.* 2014:1-11.
94. Zelazo PD, Anderson JE, Richler J, Wallner-Allen K, Beaumont JL, Conway KP, et al. NIH Toolbox Cognition Battery (CB): validation of executive function measures in adults. *Journal of the International Neuropsychological Society : JINS.* 2014;20(6):620-629.
95. Pietrzak RH, Cohen H, Snyder PJ. Spatial learning efficiency and error monitoring in normal aging: an investigation using a novel hidden maze learning test. *Archives of clinical neuropsychology : the official journal of the National Academy of Neuropsychologists.* 2007;22(2):235-245.
96. Snyder PJ, Bednar MM, Cromer JR, Maruff P. Reversal of scopolamine-induced deficits with a single dose of donepezil, an acetylcholinesterase inhibitor. *Alzheimers Dement.* 2005;1(2):126-135.
97. Celone KA, Calhoun VD, Dickerson BC, Atri A, Chua EF, Miller SL, et al. Alterations in memory networks in mild cognitive impairment and Alzheimer's disease: an independent component analysis. *Journal of Neuroscience.* 2006;26(40):10222-10231.

98. Miller SL, Celone K, DePeau K, Diamond E, Dickerson BC, Rentz D, et al. Age-related memory impairment associated with loss of parietal deactivation but preserved hippocampal activation. *ProcNatlAcadSciUSA.* 2008;105(6):2181-2186.
99. Sperling RA, Chua E, Cocchiarella A, Rand-Giovannetti E, Poldrack R, Schacter DL, et al. Putting names to faces: successful encoding of associative memories activates the anterior hippocampal formation. *Neuroimage.* 2003;20(2):1400-1410.
100. Hayes SM, Alosco ML, Hayes JP, Cadden M, Peterson KM, Allsup K, et al. Physical Activity Is Positively Associated with Episodic Memory in Aging. *Journal of the International Neuropsychological Society : JINS.* 2015;21(10):780-790.
101. Bakker A, Kirwan CB, Miller M, Stark CE. Pattern separation in the human hippocampal CA3 and dentate gyrus. *Science.* 2008;319(5870):1640-1642.
102. Stark SM, Yassa MA, Stark CE. Individual differences in spatial pattern separation performance associated with healthy aging in humans. *Learn Mem.* 2010;17(6):284-288.
103. Yassa MA, Mattfeld AT, Stark SM, Stark CE. Age-related memory deficits linked to circuit-specific disruptions in the hippocampus. *Proc Natl Acad Sci U S A.* 2011;108(21):8873-8878.
104. Yassa MA, Stark SM, Bakker A, Albert MS, Gallagher M, Stark CE. High-resolution structural and functional MRI of hippocampal CA3 and dentate gyrus in patients with amnestic Mild Cognitive Impairment. *Neuroimage.* 2010;51(3):1242-1252.
105. Bakker A, Krauss GL, Albert MS, Speck CL, Jones LR, Stark CE, et al. Reduction of hippocampal hyperactivity improves cognition in amnestic mild cognitive impairment. *Neuron.* 2012;74(3):467-474.
106. Newhouse P, Kellar K, Aisen P, White H, Wesnes K, Coderre E, et al. Nicotine treatment of mild cognitive impairment: a 6-month double-blind pilot clinical trial. *Neurology.* 2012;78(2):91-101.
107. Darby DG, Brodtmann A, Pietrzak RH, Fredrickson J, Woodward M, Villemagne VL, et al. Episodic Memory Decline Predicts Cortical Amyloid Status in Community-Dwelling Older Adults. *J Alzheimers Dis.* 2011;27(3):627-637.
108. Lim YY, Ellis KA, Pietrzak RH, Ames D, Darby D, Harrington K, et al. Stronger effect of amyloid load than APOE genotype on cognitive decline in healthy older adults. *Neurology.* 2012;79(16):1645-1652.
109. Grundman M, Petersen RC, Ferris SH, Thomas RG, Aisen PS, Bennett DA, et al. Mild cognitive impairment can be distinguished from Alzheimer disease and normal aging for clinical trials. *Arch Neurol.* 2004;61(1):59-66.
110. Galasko D, Bennett D, Sano M, Ernesto C, Thomas R, Grundman M, et al. An inventory to assess activities of daily living for clinical trials in Alzheimer's disease. The Alzheimer's Disease Cooperative Study. *Alzheimer Dis Assoc Disord.* 1997;11 Suppl 2:S33-39.
111. Cummings JL. The Neuropsychiatric Inventory: assessing psychopathology in dementia patients. *Neurology.* 1997;48(5 Suppl 6):S10-16.
112. Saykin AJ, Wishart HA, Rabin LA, Santulli RB, Flashman LA, West JD, et al. Older adults with cognitive complaints show brain atrophy similar to that of amnestic MCI. *Neurology.* 2006;67(5):834-842.
113. Snowdon J. Validity of the Geriatric Depression Scale. *J Am Geriatr Soc.* 1990;38(6):722-723.
114. McHorney CA, Ware JE, Jr., Raczek AE. The MOS 36-Item Short-Form Health Survey (SF-36): II. Psychometric and clinical tests of validity in measuring physical and mental health constructs. *Med Care.* 1993;31(3):247-263.

115. Devlin NJ, Krabbe PF. The development of new research methods for the valuation of EQ-5D-5L. *The European journal of health economics : HEPAC : health economics in prevention and care.* 2013;14 Suppl 1:S1-3.
116. Bastien CH, Vallieres A, Morin CM. Validation of the Insomnia Severity Index as an outcome measure for insomnia research. *Sleep medicine.* 2001;2(4):297-307.
117. Rosen WG, Terry RD, Fuld PA, Katzman R, Peck A. Pathological verification of ischemic score in differentiation of dementias. *Ann Neurol.* 1980;7(5):486-488.
118. Duncan BL, Miller SD, Wampold BE, Hubble MA. *The heart and soul of change: Delivering what works in therapy.* 2 ed: American Psychological Association; 2010.
119. Miller SD, Duncan BL, Sorrell R, Brown GS. The partners for change outcome management system. *Journal of clinical psychology.* 2005;61(2):199-208.
120. Arnold JB, Liow JS, Schaper KA, Stern JJ, Sled JG, Shattuck DW, et al. Qualitative and quantitative evaluation of six algorithms for correcting intensity nonuniformity effects. *Neuroimage.* 2001;13(5):931-943.
121. Jovicich J, Czanner S, Greve D, Haley E, van der Kouwe A, Gollub R, et al. Reliability in multi-site structural MRI studies: effects of gradient non-linearity correction on phantom and human data. *Neuroimage.* 2006;30(2):436-443.
122. Sled JG, Zijdenbos AP, Evans AC. A nonparametric method for automatic correction of intensity nonuniformity in MRI data. *IEEE Trans Med Imaging.* 1998;17(1):87-97.
123. Simonsick EM, Fan E, Fleg JL. Estimating cardiorespiratory fitness in well-functioning older adults: treadmill validation of the long distance corridor walk. *J Am Geriatr Soc.* 2006;54(1):127-132.
124. American College of Sports Medicine Position Stand. Exercise and physical activity for older adults. *Med Sci Sports Exerc.* 1998;30(6):992-1008.
125. Furberg CD, Manolio TA, Psaty BM, Bild DE, Borhani NO, Newman A, et al. Major electrocardiographic abnormalities in persons aged 65 years and older (the Cardiovascular Health Study). Cardiovascular Health Study Collaborative Research Group. *Am J Cardiol.* 1992;69(16):1329-1335.
126. *Exercise and the heart.* 4th ed. Philadelphia, PA: WB Saunders; 2000.

APPENDIX 1: SCHEDULE OF EVENTS

**Visit Number**	**0**	**1**	**2**	**3**	**4**	**5**
**Visit Name / Month**	**Screen I**	**Screen II**	**Baseline**	**6**	**12**[1]	**18**
**Visit Window (weeks)**				**+/- 2**	**+/- 2**	**+/- 2**
Brief Informed Consent for Screen I (screening for Screen II)	X					
Informed Consent for Enrollment, HIPAA		X				
EXERT TAPA (to confirm sedentary status)	X					
Review Inclusion & Exclusion Criteria	X	X				
Demographics	X	X				
Medical History	X	X				
Medication Review	X	X	X	X	X	X
Brief Physical Exam		X		X	X	X
Vital Signs		X	X	X	X	X
Height		X				
Weight		X	X	X	X	X
Brief Neurological Exam		X		X	X	X
Modified Hachinski		X				
Fasting Clinical Blood Labs		X			X	X
Blood Collection for ApoE Genotyping, & DNA Banking		X				
Fasting Blood Collection for Banking			X	X	X	X
AE Monitoring at Clinic			X	X	X	X
12-Lead Resting ECG		X				
3MSE/MMSE		X			X	
Logical Memory I and II		X				
Auditory Verbal Learning Test		X				
ADAS-Cog13			X	X	X	X
Trail-Making Test, DSST, Category and Letter Word Fluency			X	X	X	X
Computer Tests: NIH Toolbox (Flanker, DCCS), Cogstate (DET, IDN, OCL, OBK, GMLT, FNAME, BPSO)			X	X	X	X
CDR		X		X	X	X
ADCS-ADL-MCI, BRIEF-A, NPI (staff-administered questionnaires)			X	X	X	X
GDS (self-administered questionnaire)		X				
Cognitive Change Index (self-administered questionnaire)		X		X	X	X
SF-36, EQ5D (self-administered)			X	X	X	X
Research Satisfaction Survey (self-administered)			X	X	X	X
Study Partner Self-Assessment Questionnaire[2]			X	X	X	X
400 m Walk Test		X		X	X	X
Structural/Functional MRI[3]		X			X	
LP for CSF Collection and Banking[4]			X		X	

Visit Number	0	1	2	3	4	5
**Visit Name / Month**	**Screen I**	**Screen II**	**Baseline**	**6**	**12[1]**	**18**
**Visit Window (weeks)**				**+/- 2**	**+/- 2**	**+/- 2**
Post-LP Safety Telephone Follow-Up[4]			X		X	
Replace Battery in Heart Rate Monitor				X	X	X
Dispense Physical Activity Logs			X	X	X	
Retrieve/Review Physical Activity Logs[5]			X	X	X	X
Actigraphy Recording Initialization (dispense device)[6]		X		X	X	X
Actigraphy Device Retrieval (10 days later)[6]			X	X	X	X
Telephone Call to Participant by Clinic YMCA Liaison (query AEs, obtain medication updates)[7]			X	X	X	
Trainer-Supervised Exercise Sessions[8]			X	X	X	
Trainer-Administered Rating of Perceived Exertion[9]			X	X	X	
Trainer-Administered AE Checklist[10]			X	X	X	
Randomization			X			
Treatment Blinding Questionnaire					X	X
Provide Study Close-Out Materials						X

1 Following Month 12 assessment, participants continue with their assigned exercise program but without supervision in Months 13-18

2 The Study Partner Self-Assessment Questionnaire is comprised of the SF-36, the Pittsburgh Sleep Quality Index, the Insomnia Severity Index, the EQ5D, and questions about healthcare utilization

3 Screening MRI to be conducted following site PI confirmation that participant has met all other inclusion/exclusion criteria; fMRI sequences added to protocol for approved sites

4 To be completed for fasting participants who agree to LP at Baseline and Month 12

5 Logs reviewed by Trainers weekly (in months 1-12), and by Clinic YMCA Liaison monthly when retrieved from YMCA (months 1-18) to identify potential adherence issues

6 See Section 9.0 for visit-specific instructions

7 Telephone call to be completed monthly (months 1-18)

8 Supervision provided by study-certified YMCA Trainers, beginning after randomization through Month 12; data collected include type of exercise completed, exercise duration, HR, RPE, and other intervention-specific details (e.g., body parts stretched, treadmill speed and grade)

9 Rating scale administered as part of every supervised exercise session (months 1-12) to assess effort and estimate exercise intensity

10 Administered once per week by YMCA Trainer during supervised sessions to query change in health status in the preceding 7 days; faxed to clinic for triage and data entry within 24 hours of the session
