# Supplementary material for: Effects of exercise on cognition and Alzheimer's biomarkers in a randomized controlled trial of adults with mild cognitive impairment: The EXERT study
Source: Alzheimers Dement. 2025 Apr 24;21(4):e14586. doi: 10.1002/alz.14586 (PMC12019696; doi:10.1002/alz.14586)
Supplement: Supplementary file 5 — Supporting Information [file ALZ-21-e14586-s005.pdf]

EXERT Telephone Assessment of Physical Activity (TAPA) Questionnaire

RED: Ineligible

I am going to ask you about the types and amount of physical activity you usually do. In this questionnaire, we define physical activities as activities where you move and increase your breathing or heart rate. These are activities you do for pleasure, work, or for getting around.

I will read several statements, and for each one, please indicate whether it describes you by answering *Yes* or *No*. For example,

SAMPLE	I am over 50 years old. Does this describe you?	**Yes** □	**No** □	**Not Sure** □

Please do your best to answer my questions with a '*Yes*' or a '*No*' response. I may also ask you to list specific types of activities you do.

Here's the first statement:

1	I rarely or never do any physical activities. Does this describe you?	**Yes** □	**No** □	**Not Sure** □

The next statements are about 3 different types of activities: light, moderate, and vigorous.

Light activities are activities that make your heart beat only slightly faster than normal, and you can still easily talk or sing while doing them. Some examples of light activities are **walking leisurely, light vacuuming, light yard work, or light stretching**. Here are 2 statements about doing light activity (read statements slowly):

2	I do some **light** physical activities, but not every week. Does this describe you? (repeat examples of light activity listed above for clarification as needed)	**Yes** □	**No** □	**Not Sure** □
3	I do some **light** physical activities every week. Does this describe you? (repeat examples listed above as needed).	**Yes** □	**No** □	**Not Sure** □

Next we're going to talk about moderate activities. **Moderate activities are those that make your heart beat faster than normal**. **You can still talk but may have difficulty singing** while doing these activities. Some examples include **fast walking, aerobics classes, lifting weights, or swimming laps**. I have 3 statements about doing moderate activities. The first one is (read statements slowly):

4	I do some **moderate** physical activities, but not every week. Does this describe you? (repeat examples of moderate activities listed above for clarification as needed)	**Yes** □	**No** □	**Not Sure** □
5	I do some **moderate** physical activities every week, but for NO more than 20 minutes per day *AND* for just 1 or 2 days per week. Does this describe you? (repeat examples listed above for clarification as needed)	Yes □	**No** □	**Not Sure** □
6	I do some **moderate** physical activities every week for MORE than 20 minutes per day *OR* on more than 2 days per week. Does this describe you? (repeat examples as needed)	**Yes** □	**No** □	**Not Sure** □

If “Yes” to #5 or #6, list activities:

The next statement is about vigorous activities. **Vigorous activities are those that make your heart rate increase a lot. You typically can’t talk while doing vigorous activities, or your talking is broken up by large breaths.** Some examples of vigorous activities include **jogging, running, using an elliptical trainer, cycling classes, and vigorous games of tennis, racquetball, badminton, or pickleball**. Here’s the statement (read slowly):

7	I do some **vigorou**s physical activities <u>at least twice per month</u>. Does this describe you? (repeat examples listed above for clarification as needed)	**Yes □**	**No □**	**Not Sure □**

If “Yes” to #7, list activities:

Finally, I’m going to read a statement about muscle strengthening activities:

8	I do activities to increase muscle **strength**, such as lifting weights or calisthenics <u>at least twice per month</u>. Does this describe you?	**Yes □**	**No □**	**Not Sure □**

If “Yes” to #8, list activities:

Are there physical activities that you do that we have not captured in this questionnaire?

If “Yes”, list:

This concludes my questions. Thank you.
