# Supplementary material for: Effects of exercise on cognition and Alzheimer's biomarkers in a randomized controlled trial of adults with mild cognitive impairment: The EXERT study
Source: Alzheimers Dement. 2025 Apr 24;21(4):e14586. doi: 10.1002/alz.14586 (PMC12019696; doi:10.1002/alz.14586)
Supplement: Supplementary file 6 — Supporting Information [file ALZ-21-e14586-s003.pdf]

SIGNATURE PAGE

Protocol Title: **Therapeutic Effects of Exercise in Adults Amnestic Mild Cognitive Impairment (EXERT)**

Sponsor: ADCS/NIA

Protocol Number: ADC-041-EX

Document Version/Date: February 3, 2022

Author:
Ronald G. Thomas, Ph.D., Professor
Herbert Wertheim School of Public Health
and Human Longevity Science
University of California, San Diego

Signature: ______________________

Date: 2/3/22

Investigator Approval

By signing this document, I acknowledge that I have read the document and approve of the planned statistical analyses described herein. I agree that the planned statistical analyses are appropriate for this study, are in accordance with the study objectives, and are consistent with the statistical methodology described in the protocol.

I have discussed any questions I have regarding the contents of this document with the biostatistical author.

I also understand that any subsequent changes to the planned statistical analyses, as described herein, will be updated within any publications related to the study.

Project Director Signatories:

Laura Baker, PhD — Signature: ______________________

Date: ______________________

Carl Cotman, PhD — Signature: ______________________

Date: ______________________

ADCS Principal Investigator Signatory:

Howard Feldman, MD, Director, Alzheimer's Disease Cooperative Study — Signature: ______________________

Date: ______________________

SIGNATURE PAGE

**Protocol Title:**	**Therapeutic Effects of Exercise in Adults Amnestic Mild Cognitive Impairment (EXERT)**
**Sponsor:**	ADCS/NIA
**Protocol Number:**	ADC-041-EX
**Document Version/Date:**	February 3, 2022
**Author:**	Ronald G. Thomas, Ph.D., Professor<br>Herbert Wertheim School of Public Health<br>and Human Longevity Science<br>University of California, San Diego

Signature:____________________

Date:____________________

Investigator Approval

By signing this document, I acknowledge that I have read the document and approve of the planned statistical analyses described herein. I agree that the planned statistical analyses are appropriate for this study, are in accordance with the study objectives, and are consistent with the statistical methodology described in the protocol.

I have discussed any questions I have regarding the contents of this document with the biostatistical author.

I also understand that any subsequent changes to the planned statistical analyses, as described herein, will be updated within any publications related to the study.

Project Director Signatories:

Laura Baker, PhD	Signature:________<br>Date: 03 Feb 2022
Carl Cotman, PhD	Signature:________<br>Date:________

ADCS Principal Investigator Signatory:

Howard Feldman, MD, Director, Alzheimer's Disease Cooperative Study	Signature:________<br>Date: 03 Feb 2022

SIGNATURE PAGE

Protocol Title: **Therapeutic Effects of Exercise in Adults Amnestic Mild Cognitive Impairment (EXERT)**

Sponsor: ADCS/NIA

Protocol Number: ADC-041-EX

Document Version/Date: February 3, 2022

Author: Ronald G. Thomas, Ph.D., Professor
Herbert Wertheim School of Public Health
and Human Longevity Science
University of California, San Diego

Signature:____________________________________

Date:____

Investigator Approval

By signing this document, I acknowledge that I have read the document and approve of the planned statistical analyses described herein. I agree that the planned statistical analyses are appropriate for this study, are in accordance with the study objectives, and are consistent with the statistical methodology described in the protocol.

I have discussed any questions I have regarding the contents of this document with the biostatistical author.

I also understand that any subsequent changes to the planned statistical analyses, as described herein, will be updated within any publications related to the study.

Project Director Signatories:

Laura Baker, PhD — Signature: [signature]
Date: 03 Feb 2022

Carl Cotman, PhD — Signature: Carl W. Cotman
Date: Feb 3. 2022

ADCS Principal Investigator Signatory:

Howard Feldman, MD, Director, Alzheimer's Disease Cooperative Study — Signature:____________________________________
Date:____

ALZHEIMER'S DISEASE COOPERATIVE STUDY (ADCS)

Therapeutic Effects of Exercise in Adults with Amnestic Mild Cognitive Impairment

Statistical Analysis Plan (SAP)

February 3, 2022

**Project Principal Investigators:**	**Laura Baker, PhD, Carl Cotman, PhD**
**U-19 Principal Investigator:**	**Howard Feldman, MD**
**Analysis Team:**	**ADCS Biostatistics Core**
**Contributing Investigators (alphabetical):**	**Laura Baker, PhD**<br>**James Brewer, MD PhD**<br>**Carl Cotman, PhD**<br>**Howard Feldman, MD**<br>**Diane Jacobs, PhD**<br>**Shelia Jin, MD MPH**<br>**Youngkyoo Jung, PhD**<br>**Jeffrey Katula, PhD**<br>**Andrea LaCroix, PhD**<br>**Judy Pa, PhD**<br>**Robert Rissman**<br>**David Salmon, PhD**<br>**Aladdin Shadyab, PhD**<br>**Ron Thomas, PhD**

1. Introduction

This document outlines the Statistical Analysis Plan (SAP) for the EXERT Clinical Trial.

2. Study Design

EXERT is a multisite, randomized, single-blind trial comparing the effects of supervised moderate/high intensity aerobic exercise (70-80% Heart Rate Reserve [HRR]) versus stretching/balance (<35% HRR) in 296 adults with amnestic mild cognitive impairment (MCI).

3. Randomization

We use a randomized, single-blind study design where randomization is undertaken using a simple 1:1 schedule to either the aerobic exercise (AX) or stretching, balance and range of motion (SBR) group, and stratified by site, ApoE4 carrier status (yes or no), and sex. After 12 months of supervised exercise, participants transition to independent exercise (without supervision) and are instructed to continue their assigned exercise regimen for an additional 6 months to test the efficacy of a translational model.

4. Power and Sample Size Determination

Power calculations were based on two-sample t-tests. Sample sizes were estimated using 12-month ADAS-Cog change scores from the ADCS MCI trial, targeting power=80%, 2-sided alpha=5%, and SD=4.1. Calculations ranged over effect sizes in ADAS-Cog from 1.0–2.0, and dropout rates from between 10-25%, which are similar to other reports, including multi-center trials of physical activity interventions in older cohorts. The trial was powered to conservatively accommodate 20% attrition. Based on these considerations, and an effect size of 1.5, 291 total subjects were required. We enrolled 296 participants into the trial. The primary outcome measure is a validated composite score (ADAS-Cog-Exec)[1] derived using an optimally weighted combination of select subtest scores from the Alzheimer's Disease Assessment Scale – Cognitive Subscale, version 13 (ADAS-Cog13), supplemented with tests of executive function (Trail Making Test, Digit Symbol Substitution Test, Word Fluency), and select box scores of the Clinical Dementia Rating (CDR) scale (Memory, Orientation, Judgement and Problem-Solving). This approach that includes composite measures is expected to increase statistical power. In light of evidence showing exercise benefits on executive function, the addition of the Trail Making Test, Digit Symbol Substitution (DSST), Word Fluency, and CDR subscale scores to the ADAS-Cog-Exec composite should also increase sensitivity and power.

5. Study Aims

5.1 Primary Aim

To test the hypothesis that 12 months of supervised moderate/high intensity aerobic exercise, relative to a stretching/balance/range of motion control, improves cognitive function measured using the ADAS-Cog-Exec, in older adults with amnestic MCI.

5.2 Secondary Aims

5.2.1 **Key second aim**: to test the hypothesis that 12 months of aerobic exercise, relative to the control, improves scores on the primary outcome (ADAS-Cog-Exec) in the subset of participants who had the opportunity to complete a full 12 months of the study (i.e., 12 months of exercise and the 12 Month outcomes assessment) before the COVID-19 pandemic affected trial conduct.

5.2.2 To test whether 12 months of supervised aerobic exercise, relative to the control, improves executive function and episodic memory measured by domain-specific composite scores.

5.2.3 To test whether 12 months of aerobic exercise, relative to the control, reduces brain atrophy and increases blood flow in the hippocampus and in prefrontal (superior frontal, caudal-middle frontal, rostral-middle frontal, pars opercularis, pars triangularis) and AD signature (parahippocampus, fusiform, inferior temporal, middle temporal, inferior-parietal) composite regions, measured using volumetric and arterial spin labeling magnetic resonance imaging (MRI).

5.2.4 To test whether 12 months of aerobic exercise, relative to the control, favorably alters AD biomarkers in CSF (ab42/ab40, ab42/tau, ab42/p-tau) and blood (ab42/ab40).

5.3 Exploratory Aims

5.3.1 To examine intervention effects on secondary outcomes listed above (**Section 5.2.2-5.2.4**) in participants who had the opportunity to complete a full 12 months of the study before the pandemic affected trial conduct.

5.3.2 To test whether 12 months of aerobic exercise, relative to the control, favorably affects MRI whole brain, ventricular and entorhinal volumes; perfusion in whole brain, gray matter and white matter; and individual AD biomarkers in CSF (ab42, ab40, total tau, p-tau, BDNF) and blood (ab42, ab40).

5.3.3 To test whether 12 months of aerobic exercise, relative to the control, reduces clinical ratings of cognitive impairment as measured by the CDR – Sum of Boxes, and total score on the ADAS-Cog13.

5.3.4 To test whether 12 months of aerobic exercise, relative to the control, improves self-report measures of cognitive function and well-being, including (1) daily living skills (ADCS–Activities of Daily Living–MCI); (2) BRIEF-A: Behavior Rating Inventory of Executive Function–Adult Version); (3) mood (GDS); (4) health-related quality of life (NPI: Neuropsychiatric Inventory; SF-36: 36-Item Short Form Health Survey; EuroQol: 5-Item Health Questionnaire); (5) subjective memory concerns (CCI: Cognitive Change Index); and (6) Study Partner Self-Assessment Questionnaire.

5.3.5 To examine enduring cognitive effects (measured by ADAS-Cog-Exec, Executive Function and Episodic Memory Composites) of the intervention following a 6-month extension (through Month 18) when the prescribed

exercise is continued without supervision.

5.3.6 To explore whether sex, age, baseline AD biomarker profile in CSF (ab42/ab40, ab42/tau, ab42/p-tau) and blood (ab42/ab40), and ApoE4 genotype (e4+, e4-) predict treatment response.

6. Populations of Interest

6.1 Modified Intent-to-Treat (mITT) Population: Includes all eligible participants who (1) began the exercise intervention (Intervention Session 1) and (2) completed at least one post-baseline assessment for the primary analysis at Month 6 or Month 12. All consented randomized participants are grouped according to the treatment assigned at randomization, regardless of any protocol violations.

6.1.1 mITT Pre-Pandemic Subset: Subset of mITT participants who had the opportunity to complete a full 12 months of the study prior to the pandemic-related study pause on March 23, 2020. Individuals are included in this subset if they (1) completed the Month 12 primary outcomes assessment before the study pause, (2) completed an early termination visit before the study pause, or (3) withdrew consent, were lost to follow-up, or died before the study pause.

6.1.2 mITT Pandemic Subset: mITT participants who did not have the opportunity to complete a full 12 months of the study prior to the pandemic-related study pause on March 23, 2020. Individuals are included in this subset if they are not a member of the Pre-Pandemic Subset.

6.2 Per-Protocol (PP) Population: Includes all eligible participants meeting <u>all three</u> of the following criteria: (1) completed the 12-month assessment for the primary analysis, (2) attended at least 70% of the supervised exercise sessions, and (3) completed a mean total supervised exercise time of at least 40 minutes.

6.3 Intent-to-treat (ITT) Population: Includes all eligible and randomized individuals.

6.4 Safety Population: Includes all randomized participants who participated in at least one exercise session (Intervention Session 1).

7. Analyses Populations: Definitions

7.1 Clinical Outcome Analyses: The primary, secondary, and exploratory clinical outcome analyses will be conducted in the mITT Population, the mITT Pre-Pandemic Subset, the mITT Pandemic Subset, the ITT Population, and the Per-Protocol Population.

7.2 Safety Analyses: The primary safety analyses will include the Safety Population.

8. Enrollment and Participant Flow

8.1 Accrual of the Study

Tables will summarize accrual by study site, and figures will summarize the overall rate of accrual over calendar time. The observed rate and projected rate of accrual will be displayed in a graph (the projected rate assumes uniform accrual over time).

8.2 Study Flow CONSORT Diagram

A description of participant flow per the CONSORT guidelines[2] and checklist will be provided. The diagram will describe study status from screening to the end of the study. At each stage, reasons for persons not moving forward will be summarized by frequency and category. The diagram will include the following information:

- Number and reasons for those who screen failed
- Number of participants randomized
- Number of participants who completed the Month 12 assessments
- Number of participants who completed the Month 12 assessments before the study pause (Pre-Pandemic Subset) and after the study pause (Pandemic Subset)
- Number and reasons for participants who discontinued study before the Month 12 assessments for the entire cohort, and for the Pre-Pandemic and Pandemic Subsets

The CONSORT diagram is separated by study arm after the randomization step.

9. Early Termination

Early termination from the study includes participants who leave the study and/or discontinue treatment prior to completing the Month 12 assessments. Proportions of participants who prematurely discontinue the study prior to the Month 12 assessments will be compared between the study arms using Fisher's exact test.

10. Evaluation of Demographics and Baseline Characteristics

Tables will summarize the mITT Population, the mITT Pre-Pandemic Subset, and the mITT Pandemic Subset at baseline, and by study arm. Tables will also summarize the ITT and Per-Protocol Populations. Descriptive statistics will be presented as N, mean, standard deviation, minimum, 25th quartile, median 75th quartile and maximum for continuous variables and frequency tables (row, column percentages) for categorical variables. Statistical comparisons will be performed between randomized arms using Wilcoxon Rank Sum Test (for continuous variables) or Fisher's exact test (for categorical variables).

- Baseline Demographics: All variables collected in the Participant Demographics form
- Baseline Medical History: All variables collected in the Medical History Form
- Baseline Vital Signs: All variables collected in the Orthostatic Vital Signs Form
- Baseline Clinical Measures

11. Analyses of Study Outcomes

11.1 Analysis of the Primary Outcome

To address the aim for the primary analysis (**Aim 1**), longitudinal scores from baseline through Month 12 will be analyzed using a mixed effects model for repeated measures (MMRM) to test differences in ADAS-Cog-Exec (composite) change scores between intervention groups in the mITT Population, with type I error level set at 0.05.

The MMRM model includes terms for time, intervention assignment, and baseline ADAS-Cog-Exec plus covariates that include site, ApoE4 carrier status, sex, and additional covariates meeting the criteria below. The primary test compares group changes from baseline to the mean score obtained across the Month 6 and Month 12 assessments. When Month 6 or Month 12 data are missing for a participant, the endpoint consists of the score obtained at the available assessment visit.

When an individual test score of the ADAS-Cog-Exec composite is missing or not administered and the examiner indicated it is missing “for cognitive reasons”, the worst possible score is assigned for that individual test prior to constructing the ADAS-Cog-Exec score.

Potential confounders (baseline Walk Test time, age, education, 3MSE baseline score, and use of cholinesterase inhibitors) will be included in efficacy analyses as covariates if the following two conditions are satisfied: (1) imbalance at baseline ($p<0.1$) and, (2) association between the covariate and the response ($p<0.15$). Safety data will be analyzed using exact contingency table methods by intervention group assignment.

11.2 Analyses of Key Secondary Outcomes

11.2.1 **A key second outcome** is the intervention-related change in the ADAS-Cog-Exec for the mITT Pre-Pandemic Subset, which will be examined using the same analytic approach described for Aim 1.

11.2.2 Other secondary outcomes to be examined in the mITT Population, using a similar analytic approach as described for Aim 1, include:

11.2.2.1 Executive Function and Episodic Memory Composites (details in **Section 13**)

11.2.2.2 MRI measures of regional atrophy and blood flow in the hippocampus, and in prefrontal (superior frontal, caudal-middle frontal, rostral-middle frontal, pars opercularis, pars triangularis) and AD signature (parahippocampus, fusiform, inferior temporal, middle temporal, inferior-parietal) meta-ROI composites (details in **Section 14**)

11.2.2.3 AD biomarkers in CSF (ab42/ab40, ab42/tau, ab42/p-tau) and blood (ab42/ab40)

No adjustment to type 1 error level (α=0.05) will be made for secondary analyses.

11.3 Exploratory Analyses

Exploratory analyses will be conducted using the same approach described for Aim 1.

11.3.1 Test intervention effects in the mITT Pre-Pandemic Subset on the following outcomes:

11.3.1.1 Executive Function and Episodic Memory Composites

11.3.1.2 MRI measures of regional atrophy and blood flow in the hippocampus, and in prefrontal (superior frontal, caudal-middle frontal, rostral-middle

frontal, pars opercularis, pars triangularis) and AD signature (parahippocampus, fusiform, inferior temporal, middle temporal, inferior-parietal) meta-ROI composites

11.3.1.3 AD biomarkers in CSF (ab42/ab40, ab42/tau, ab42/p-tau) and blood (ab42/ab40)

11.3.2 Test intervention effects on MRI measures of whole brain, ventricular and entorhinal volumes, cerebral blood flow in the hippocampus, and perfusion in whole brain, gray matter and white matter in the mITT Population and Pre-Pandemic Subset.

11.3.3 Test intervention effects on individual AD biomarkers in CSF (ab42, ab40, total tau, p-tau, BDNF) and blood (ab42, ab40) in the mITT Population and Pre-Pandemic Subset.

11.3.4 Test intervention effects on additional cognitive and self-report outcomes in the mITT Population and Pre-Pandemic Subset, which include:

11.3.4.1 <u>Clinical scales/tests</u>: Clinical Dementia Rating Scale – Sum of Boxes (CDR-SB), ADAS-Cog13 total score

11.3.4.2 <u>Self-report data</u>: ADCS–ADL–MCI, BRIEF-A, GDS, NPI, SF-36, EuroQol, CCI, Study Partner Self-Assessment Questionnaire

11.3.5 Test intervention effects on cognition (ADAS-Cog-Exec, Executive Function Composite, Episodic Memory Composite) from Month 12 to Month 18 when exercise was completed without supervision, in the mITT Population, the Pre-Pandemic Subset and the Pandemic Subset.

All primary, secondary and exploratory analyses will be repeated in the ITT and Per-Protocol Populations.

12 Sensitivity Analyses

12.1 For the primary and key secondary analyses of cognitive outcomes (ADAS-Cog-Exec, Executive Function and Episodic Memory Composites), sensitivity analyses will be conducted to examine the potential effect of time (Month 6, Month 12) on intervention response. For these analyses, time will be treated as categorical, and modeled as a continuous factor. If the longitudinal patterns over time are markedly non-linear, such as might occur due to learning effects, a quadratic term for time will be included in a sensitivity analysis for the primary and secondary cognitive outcomes. In this case the intervention-by-time linear term will assess whether there is a relative difference between intervention groups that grows linearly with time.

12.2 Intervention effects on a supplemented ADAS-Cog-Exec that includes Letter Fluency (mean of F and L Fluency) will be examined in the mITT Population, the Pre-Pandemic Subset, and the Pandemic Subset using a similar approach described for Aim 1.

12.3 Intervention effects on the primary and key secondary outcomes will be examined in the Pandemic Subset using the approach described for Aim 1.

12.4 Subgroup analyses testing whether the following variables predict cognitive, vMRI, ASL MRI, and specimen biomarker response to the intervention in the mITT

Population and Pre-Pandemic Subset: sex, age, APOE genotype, baseline biomarker status (CSF: ab42/ab40, ab42/tau, ab42/p-tau; and blood: ab42/ab40).

12.5 In the Pandemic Subset, test whether pandemic-time-in-study altered intervention response on primary and key secondary outcomes. Pandemic-time-in-study coding is provided below.

- **Code 1:** At the time of study pause, participant had completed the Month 6 outcomes assessment (but not the Month 12 outcomes assessment)
- **Code 2:** At the time of study pause, participant was due for Month 6 or Month 12 outcomes assessment but did not complete until after the pause following a brief (6-8 weeks) ramp-up to prescribed intervention intensity.
- **Code 3:** At the time of study pause, participant had not yet completed an outcomes assessment.

13 Analyses of Executive Function and Episodic Memory Composites

Individual test scores included in each composite are listed below. Test scores are converted to z-scores and then averaged to create the composite score. Z-scores for longitudinal change are calculated by subtracting each participant's baseline score from the follow-up score (Month 6, Month 12) to obtain a change score, then dividing each change score by the baseline standard deviation for that measure. Composite scores are computed when 50% or more of the component test scores are available.

13.1 Executive Function Composite

1) Trails B: time (adjusted so higher values reflect better performance)
2) Digit Symbol Substitution Test (DSST): total correct
3) Category Fluency: mean of Animal and Vegetable Fluency
4) Letter Fluency: mean of F and L Fluency
5) ADAS-Cog 13 Number Cancellation: scaled score (range: 0-5; adjusted so higher values reflect better performance)
6) NIH Toolbox Flanker: total corrected score (Toolbox variable= "CSCORE"; variable takes accuracy and RT into consideration and higher scores reflect better performance)
7) NIH Toolbox Dimensional Change Card Sort (DCCS): total corrected score (toolbox variable= "CSCORE"; variable takes accuracy and RT into consideration and higher scores reflect better performance)
8) CogState One Back (ONB): arcsine square root proportion correct (CogState transformed variable= "ONB ACC")

13.2 Episodic Memory Composite

1) Immediate Word Recall (from ADAS-Cog-Exec): mean raw score of 3 trials (adjusted so higher values reflect better performance)
2) Delayed Word Recall (from ADAS-Cog-Exec): total raw score (adjusted so higher values reflect better performance)
3) CogState Face-Name Associative Memory Exam (FNAME): mean of Face Naming Learning Test Accuracy (CogState variable= "FNLT ACC") and Face Naming Matching Test accuracy (CogState variable= "FNMT ACC") (i.e., (FNLT ACC + FNMT ACC) / 2)

4) CogState Behavioral Pattern Separation of Objects (BPSO): probability of naming Distractor/Lure stimulus "Similar" minus the probability of naming a new stimulus "Similar" (CogState pattern separation variable= "BPXT METRIC"; higher scores reflect better performance)
5) CogState One Card Learning (OCL): arcsine square root proportion correct (CogState transformed variable= "OCL ACC")

14 Imaging Analyses

14.1 Volumetric MRI

The intervention effect will be assessed by examining group differences from baseline to Month 12 in mean volumetric MRI change values in three key regions: hippocampus, AD signature composite,[3] and prefrontal composite. Regions included in each meta-ROI composite are listed below (Desikan brain atlas). Composite values will reflect mean volumes across left and right brain hemispheres.

- **AD Signature Composite**

1. parahippocampus
2. fusiform
3. inferior temporal
4. middle temporal
5. inferior-parietal

- **Prefrontal Composite**

1. superior frontal
2. caudal-middle frontal
3. rostral-middle frontal
4. pars opercularis
5. pars triangularis

Additional exploratory vMRI measures to be examined include whole brain, ventricular and entorhinal volumes.

Hippocampal volume derived from MRI correlates with histological hippocampal volume and degree of neuronal loss and AD pathology appear to be an early and sensitive indicator of neurodegeneration associated with AD. Exercise effects on cortical thickness have previously been reported in prefrontal brain regions, particularly in dorsolateral areas.[4-6] Longitudinal MRI measures of regional and whole brain volumetric change provide a valuable complement to cognitive measures in that they are not influenced by temporary symptomatic improvements, and they may provide an early index of the intervention's ability to influence AD-related atrophy.

The analysis of structural change will be performed through Quarc nonlinear registration[7] comparing each participant's follow-up scan to the initial baseline scan. The Month 12 scan will be registered to the baseline scan. The procedure provides percent deformation within regions of interest (ROIs) obtained through segmentation of the baseline scan. The registration of serially acquired brain volumes yields a deformation field that represents the volumetric shift of internal structure required for optimal overlaying of the two scans. The anatomically based segmentation is overlaid on the smoothed voxel-wise deformation field and an average percent

deformation is created for each ROI. Visual QC of registration is performed and values from scans passing QC are returned to the statistical team for analysis.

The number of images that are not quantifiable due to scan alignment failure will be summarized by study arm.

14.2 ASL MRI

ASL MRI of the brain is performed to quantify cerebral blood flow (CBF). The intervention effect will be assessed by examining group differences in mean change in CBF values from baseline to Month 12. The key ASL MRI outcomes include hippocampal CBF, and perfusion in the prefrontal and AD signature meta-ROI composites that were described for the volumetric MRI analyses. Composites for these analyses, described again below, reflect mean CBF values across left and right brain hemispheres.

- **AD Signature Composite**: parahippocampus, fusiform, inferior temporal, middle temporal, inferior-parietal

- **Prefrontal Composite**: superior frontal, caudal-middle frontal, rostral-middle frontal, pars opercularis, pars triangularis

Exploratory outcomes include whole brain, gray matter, and white matter perfusion, and voxel-wise analysis of intervention-related changes in CBF.

Change in CBF appears to be an early and sensitive indicator of neurodegeneration associated with AD and may provide an early index of the intervention’s ability to alter AD-related atrophy and brain function. CBF changes in prefrontal regions have also been reported in smaller studies of aerobic exercise in adults with MCI.

All ASL MRI will undergo quality control (QC) for image artifacts, tagging error, excessive motion, registration between ASL and volumetric MRIs, and other factors. Images passing QC checks will be processed through an in-house program that Dr. Jung developed, which includes quantification of CBF into a physiological unit (ml/100g tissue/min), co-registration with T1w structural images, and partial volume correction. The baseline and follow-up CBF images will be normalized into a standard image template (i.e., MNI template) using SPM. The CBF values in prefrontal and AD signature composite regions, hippocampus, and whole brain gray and white matter will be computed and returned to the statistical team for analysis. The exploratory voxel-wise linear regression that contrasts intervention effects will be performed after site variations are harmonized and covariates (identified above for primary analysis) are adjusted. A minimum cluster size will be specified to control false positives.

The number of images that are not quantifiable due to scan alignment failure will be summarized by study arm.

15 Evaluation of Safety Measures

15.1 Event and participant count of the following will be summarized overall and by treatment group for the entire cohort, and for the pre-pandemic subset:

- AE: Overall and by MedDRA System Organ Class

- AE: MedDRA Preferred Term
- SAE: Overall and by MedDRA System Organ Class
- SAE: MedDRA Preferred Term
- SAE Definitely Related to Intervention: Overall and by System Organ Class
- Hospitalization
- Deaths

15.2 Comparisons of the number of participants with at least one AE, SAE, SAE definitely related to Intervention and Death will be examined between Intervention groups using the Fisher's Exact test.

15.3 Change in vital signs (blood pressure, weight, pulse, temperature) will be compared between treatment groups using the Wilcoxon Rank-Sum test.

16 Software

Statistical software R (version 4.1.2) will be used *http:www.r-project.org.*

17 References

1. Jacobs DM, Thomas RG, Salmon DP, et al. Development of a novel cognitive composite outcome to assess therapeutic effects of exercise in the EXERT trial for adults with MCI: The ADAS-Cog-Exec. *Alzheimers Dement (N Y).* 2020;6(1):e12059.
2. Begg C, Cho M, Eastwood S, et al. Improving the quality of reporting of randomized controlled trials. The CONSORT statement. *Jama.* 1996;276(8):637-639.
3. Schwarz CG, Gunter JL, Wiste HJ, et al. A large-scale comparison of cortical thickness and volume methods for measuring Alzheimer's disease severity. *Neuroimage Clin.* 2016;11:802-812.
4. Weinstein AM, Voss MW, Prakash RS, et al. The association between aerobic fitness and executive function is mediated by prefrontal cortex volume. *Brain Behav Immun.* 2012;26(5):811-819.
5. Jonasson LS, Nyberg L, Kramer AF, Lundquist A, Riklund K, Boraxbekk CJ. Aerobic Exercise Intervention, Cognitive Performance, and Brain Structure: Results from the Physical Influences on Brain in Aging (PHIBRA) Study. *Front Aging Neurosci.* 2016;8:336.
6. Hayes SM, Hayes JP, Cadden M, Verfaellie M. A review of cardiorespiratory fitness-related neuroplasticity in the aging brain. *Front Aging Neurosci.* 2013;5:31.
7. Holland D, Dale AM. Nonlinear registration of longitudinal images and measurement of change in regions of interest. *Med Image Anal.* 2011;15(4):489-497.

ALZHEIMER'S DISEASE COOPERATIVE STUDY (ADCS)

Therapeutic Effects of Exercise in Adults with Amnestic Mild Cognitive Impairment (EXERT)

Statistical Analysis Plan (SAP) – ERRATUM
November 25, 2024

Summary: Linear regression analyses (not MMRM) were used from the outset to examine intervention effects on EXERT outcomes as the *a priori* planned model specified use of change scores (normalized to baseline) rather than repeated measures.

Relevant erroneous text (from document version February 3, 2022):

11.1 Analysis of the Primary Outcome (page 8)

To address the aim for the primary analysis (Aim 1), longitudinal scores from baseline through Month 12 will be analyzed using a mixed effects model for repeated measures (MMRM) to test differences in ADAS-Cog-Exec (composite) change scores between intervention groups in the mITT Population, with type I error level set at 0.05.

The MMRM model includes terms for time, intervention assignment, and baseline ADAS-Cog-Exec plus covariates that include site, ApoE4 carrier status, sex, and additional covariates meeting the criteria below.

Corrected descriptions of completed analyses:

11.1 Analysis of the Primary Outcome

The ADAS-Cog-Exec is a weighted change score from baseline resulting in one score per participant.

To address the aim for the primary analysis (Aim 1), longitudinal scores from baseline through Month 12 will be analyzed using linear regression to test differences in ADAS-Cog-Exec (composite) change scores between intervention groups in the mITT Population, with type I error level set at 0.05.

The linear regression model included terms for intervention assignment plus covariates consisting of site, ApoE4 carrier status, sex, and additional covariates meeting the criteria below.
